# Supplementary material for: In vivo study of gene expression with an enhanced dual-color fluorescent transcriptional timer
Source: eLife. 2019 May 29;8:e46181. doi: 10.7554/eLife.46181 (PMC6660218; doi:10.7554/eLife.46181)
Supplement: Supplementary file 1. — (Gal4s analyzed in the main figures were not listed.) [file elife-46181-supp1.docx]

|  | **DAPI** | **GFP** | **RFP** | **Overlay** |
| --- | --- | --- | --- | --- |
| **1**  *Trx-Gal4*  *(BL40367)*  larval brain |  | 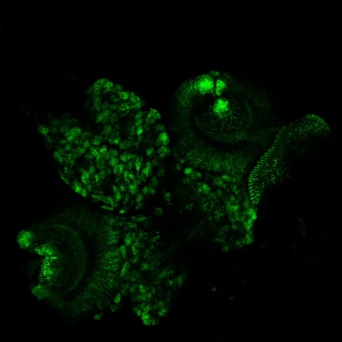 | 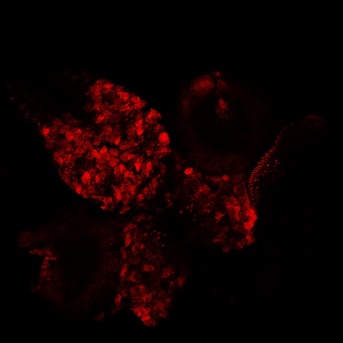 | 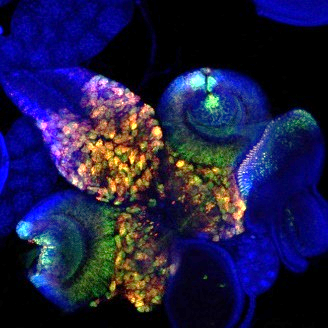 |
| **2**  *Ogre-Gal4*  *(BL49340)*  larval brain |  | 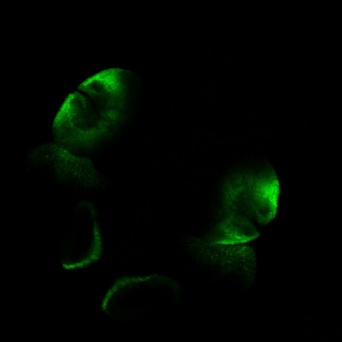 | 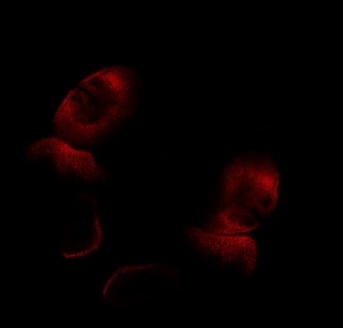 | 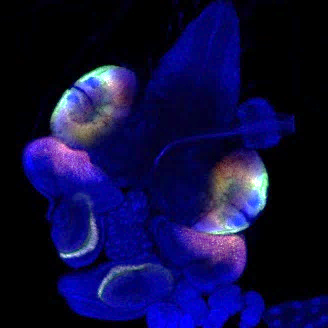 |
| **3**  *Rho-Gal4*  *(BL45254)*  larval brain | 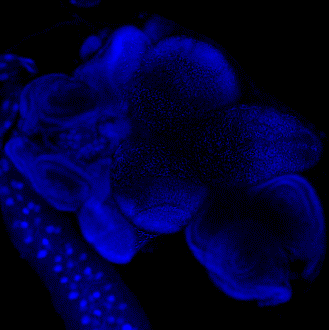 | 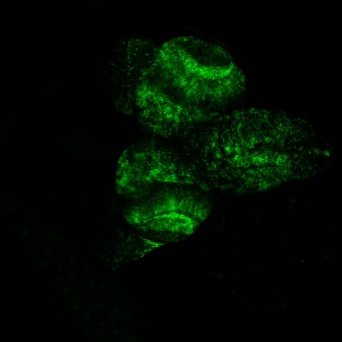 | 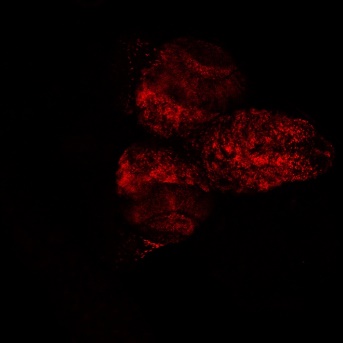 | 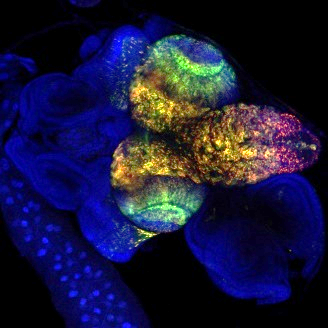 |
| **4**  *Gcm-Gal4*  *(BL35541)*  larval brain |  | 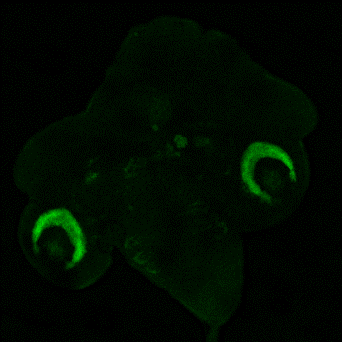 | 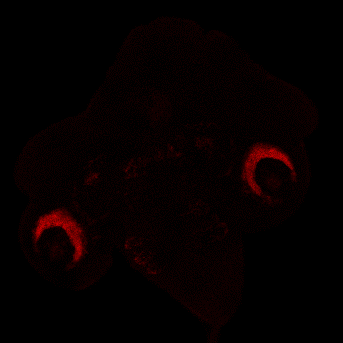 | 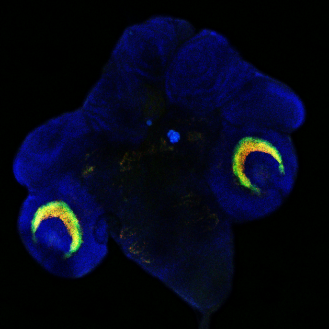 |
| **5**  *dMyc-Gal4*  *(BL47844)*  larval brain |  |  |  | 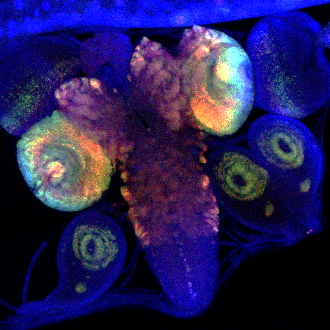 |
| **6**  *Pros-Gal4*  larval brain | 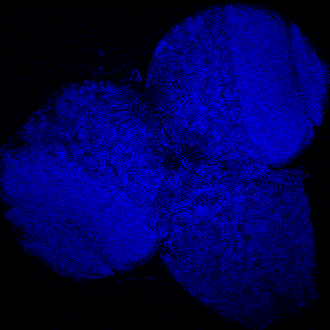 |  |  | 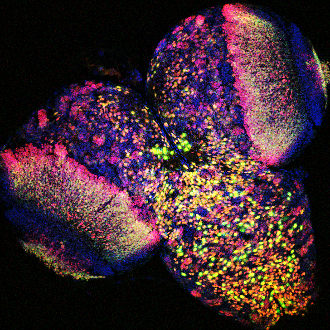 |
| **7**  *Hh-Gal4*  *(BL49437)*  larval eye disc |  | 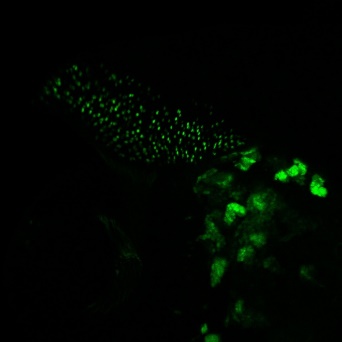 | 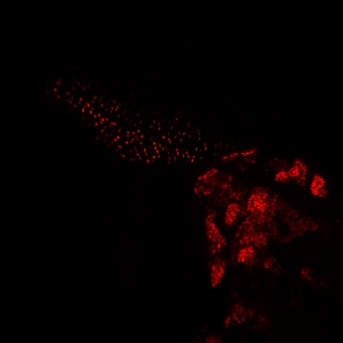 | 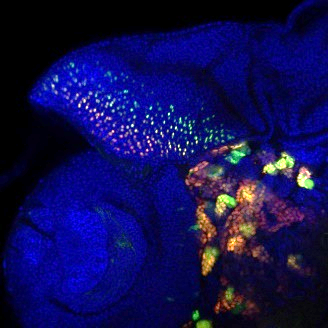 |
| **8**  *Antp-Gal4*  *(BL26817)*  larval wing disc |  | 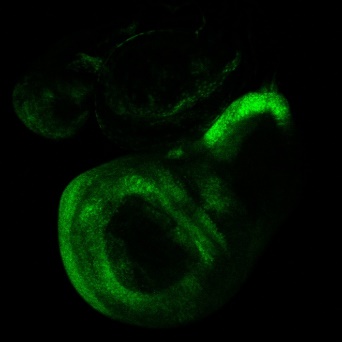 | 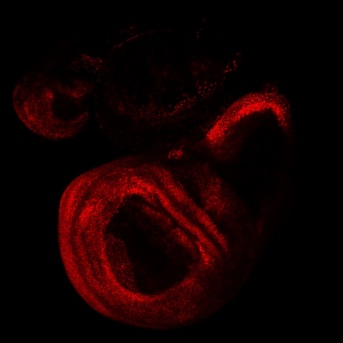 | 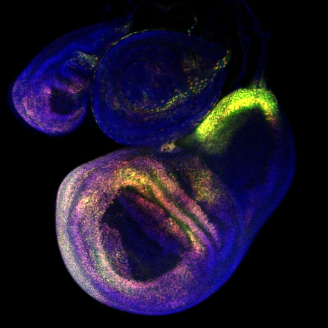 |
| **9**  *CG43980-Gal4*  *(BL66863)*  larval wing disc |  |  |  | 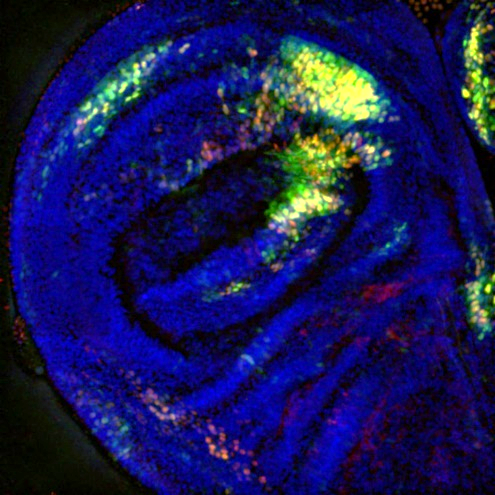 |
| **10**  *CG40006-Gal4*  *(BL76214)*  larval wing disc |  |  |  | 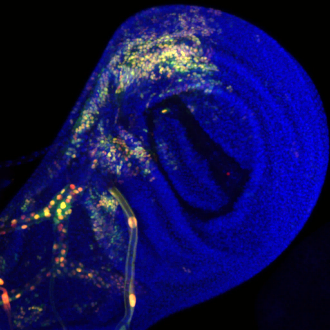 |
| **11**  *CG33964-Gal4*  *(BL76742)*  larval wing disc |  |  |  | 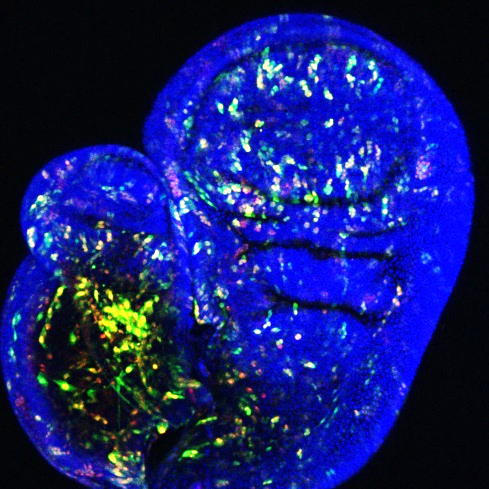 |
| **12**  *CG15270-Gal4*  *(BL76649)*  larval midgut |  |  |  | 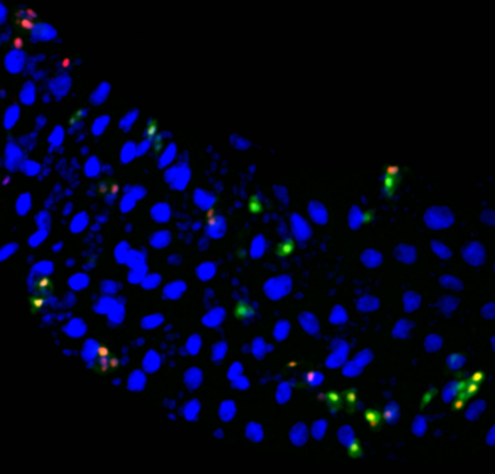 |
| **13**  *CG33964-Gal4*  *(BL76742)*  larval midgut |  |  |  | 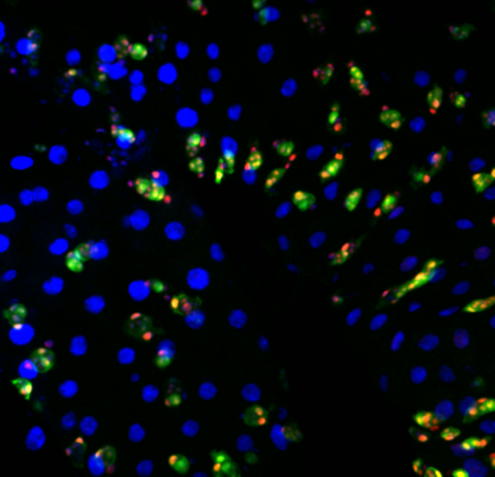 |
| **14**  *CG14995-Gal4*  *(BL76721)*  larval midgut |  |  |  | 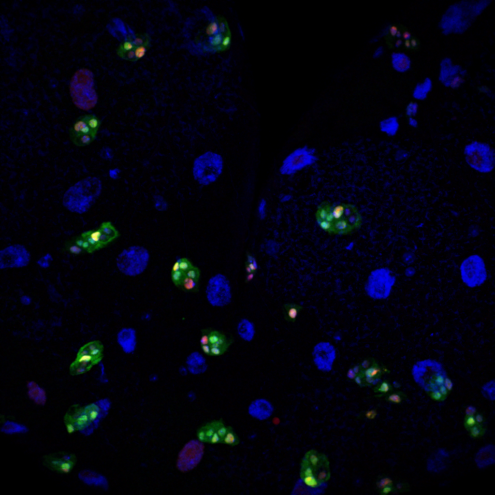 |
| **15**  *CG8270-Gal4*  *(BL77741)*  larval midgut |  |  |  | 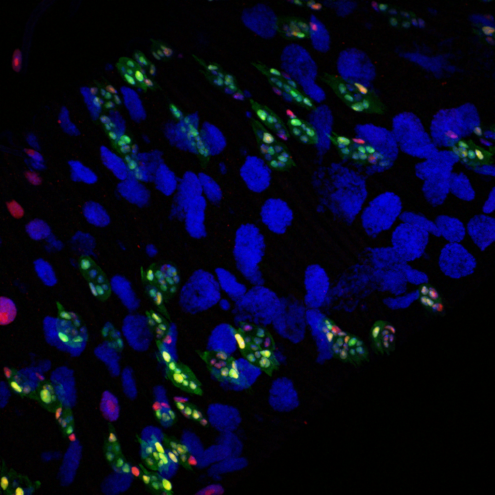 |
| **16**  *CG8177-Gal4*  *(BL77781)*  larval midgut |  |  |  | 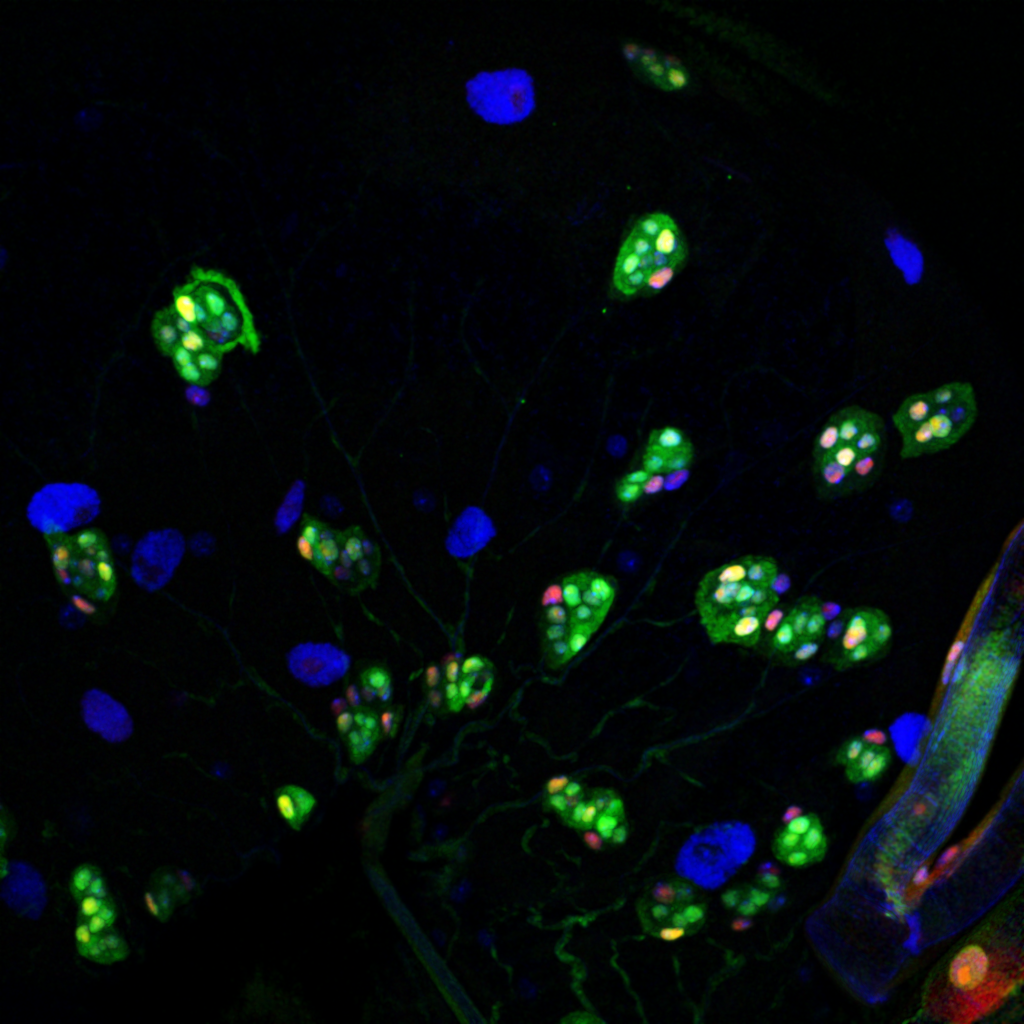 |
| **17**  *CG5521-Gal4*  *(BL76180)*  larval midgut |  |  |  | 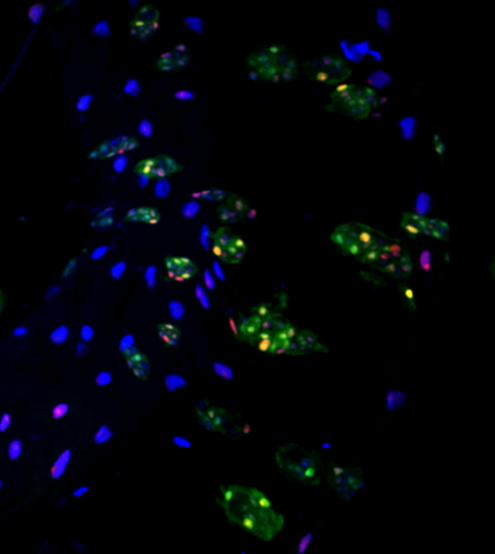 |
| **18**  *dMyc-Gal4*  *(BL47844)*  adult midgut |  | 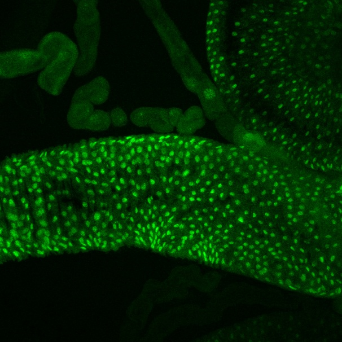 | 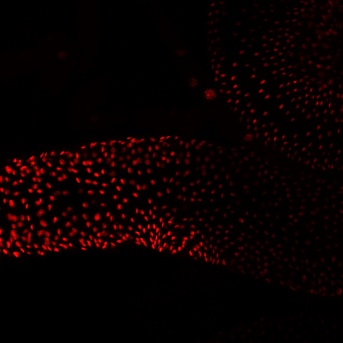 | 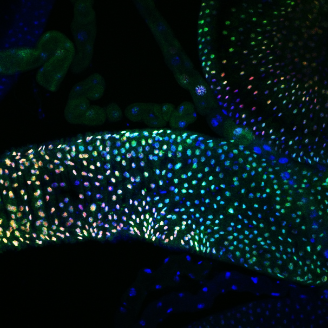 |
| **19**  *ZnT41F-Gal4*  *(BL66859)*  adult midgut |  |  |  | 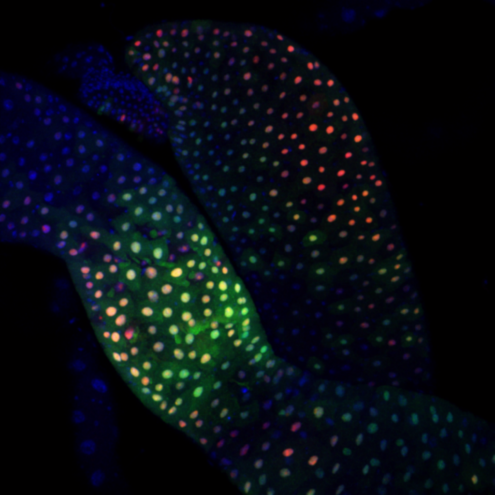 |
| **20**  *ppk-Gal4*  *(BL32078)*  adult midgut |  |  |  | 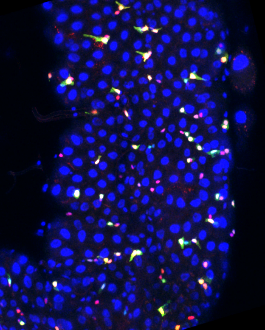 |
| **21**  *CG8177-Gal4*  *(BL77781)*  adult midgut |  |  |  | 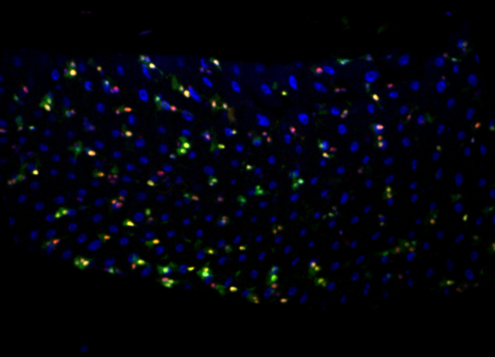 |
| **22**  *CG34347-Gal4*  *(**BL76674)*  adult midgut |  |  |  | 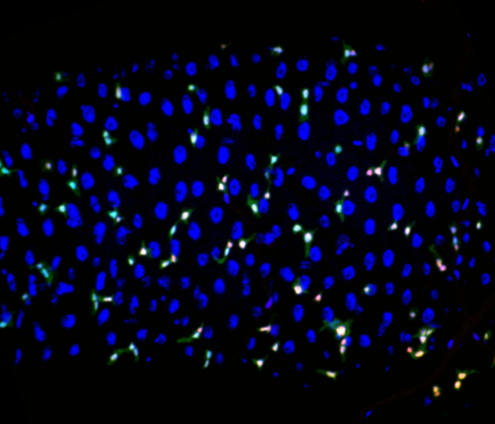 |
| **23**  *Plc21C-Gal4*  *(BL**76142)*  adult midgut |  |  |  | 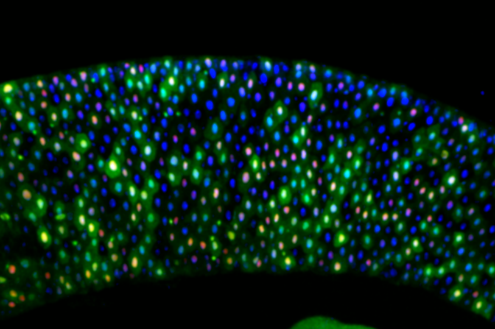 |
| **24**  *anchor-Gal4*  *(BL**66861)*  adult midgut |  |  |  | 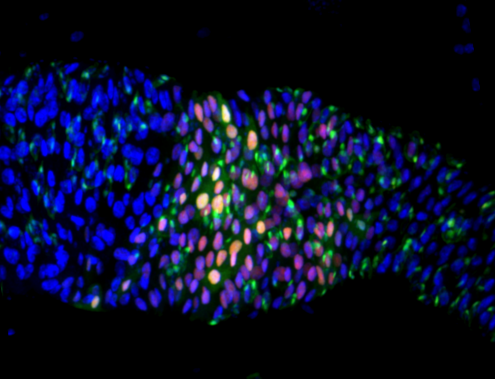 |
| **25**  *MESK2-Gal4*  *(BL67434)*  adult midgut |  |  |  | 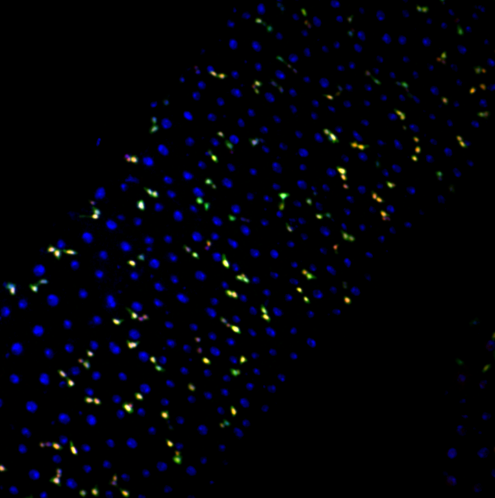 |
| **26**  *fz-Gal4*  *(**BL66817)*  adult midgut |  |  |  |  |
| **27**  *Mip-Gal4*  *(**BL51984)*  adult midgut |  |  |  |  |
| **28**  *igl-Gal4*  *(**BL76744)*  adult midgut |  |  |  |  |
| **29**  *CG4467-Gal4*  *(**BL66843)*  adult midgut |  |  |  |  |
| **30**  *CG8270-Gal4*  *(BL77741)*  adult midgut |  |  |  |  |
| **31**  *CG13175-Gal4*  *(**BL76742)*  adult midgut |  |  |  |  |
| **32**  *tutl-Gal4*  *(**BL66824)*  adult midgut |  |  |  |  |
