# Supplementary material for: In vivo study of gene expression with an enhanced dual-color fluorescent transcriptional timer
Source: eLife. 2019 May 29;8:e46181. doi: 10.7554/eLife.46181 (PMC6660218; doi:10.7554/eLife.46181)
Supplement: Supplementary file 2. [file elife-46181-supp2.docx]

|  | **DAPI** | **GFP** | **RFP** | **Overlay** |
| --- | --- | --- | --- | --- |
| **1**  *da-Gal4*  *(BL55850)*  larval salivary glands | 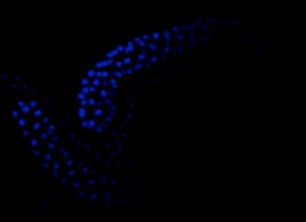 | 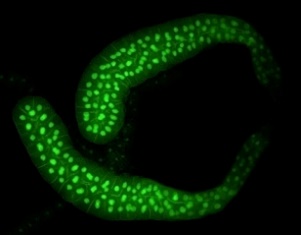 | 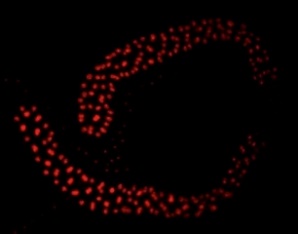 | 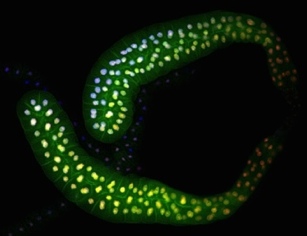 |
| **2**  *tub-Gal4*  *(BL5138)*  larval salivary glands/fat body | 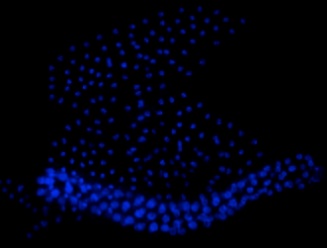 | 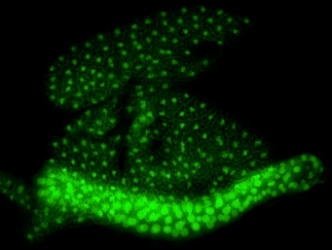 | 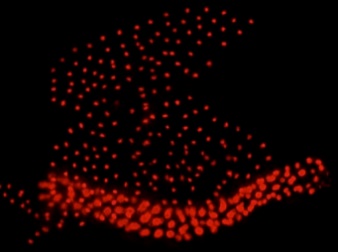 | 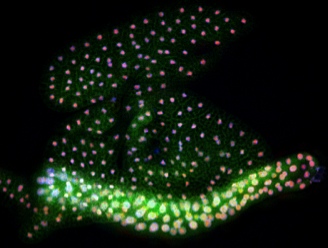 |
| **3**  *act5C-Gal4*  *(BL4414)*  larval fat body | 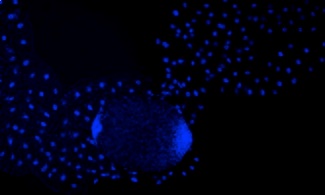 | 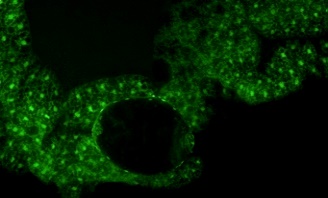 | 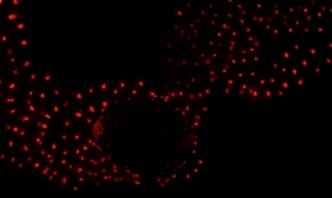 | 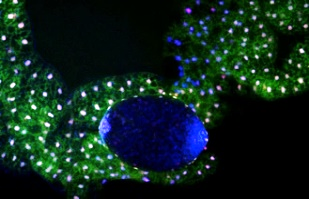 |
| **4**  *act5C-Gal4*  *(BL4414)*  larval foregut | 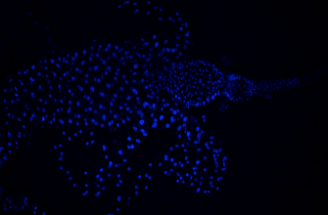 | 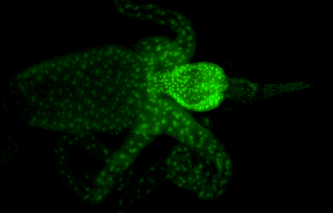 | 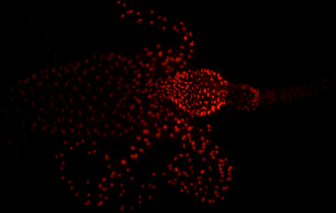 | 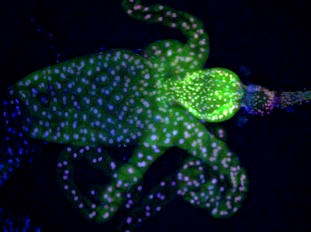 |
| **5**  *da-Gal4*  *(BL55850)*  larval hindgut | 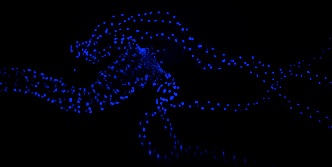 | 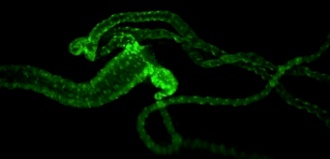 | 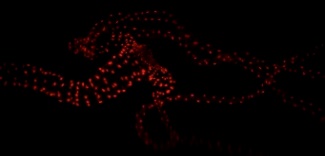 | 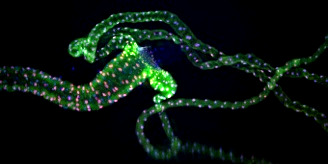 |
| **6**  *act5C-Gal4*  *(BL4414)*  larval wing disc |  |  | 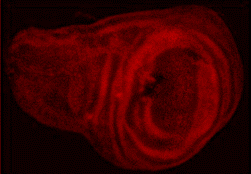 | 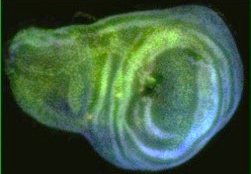 |
| **7**  *tub-Gal4*  *(BL5138)*  larval wing disc | 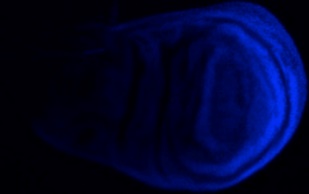 | 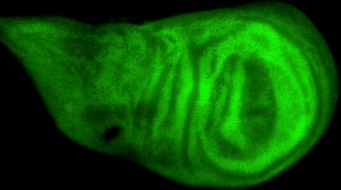 | 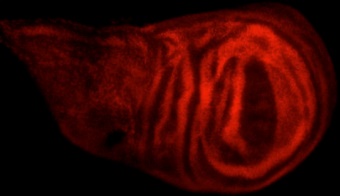 | 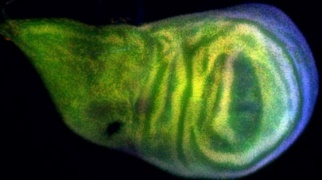 |
| **8**  *ubi-Gal4*  *(BL32551)*  larval brain | 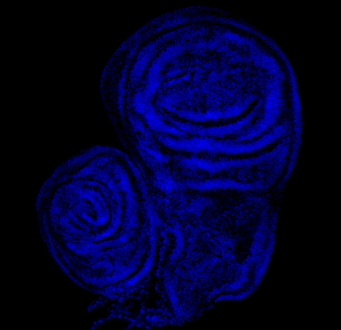 |  |  | 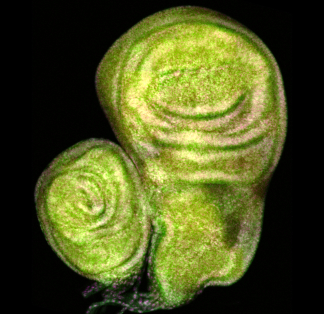 |
| **9**  *tub-Gal4*  *(BL5138)*  larval brain | 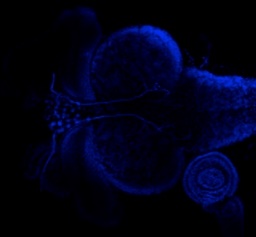 | 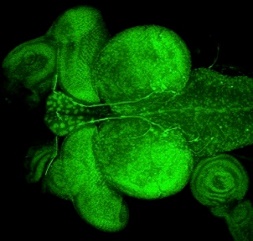 | 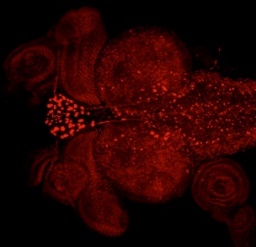 | 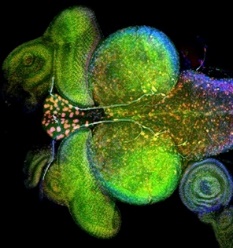 |
| **10**  *tub-Gal4*  *(BL5138)*  larval eye disc |  |  |  | 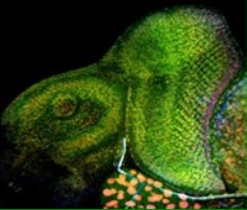 |
| **11**  *act5C-Gal4*  *(BL4414)*  larval brain |  |  |  | 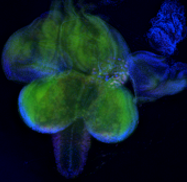 |
| **12**  *dMef-Gal4*  *(BL27390)*  larval muscle |  |  |  | 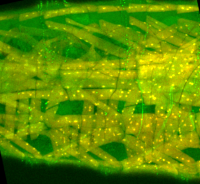 |
| **13**  *Dorothy-Gal4*  *(BL6903)*  adult midgut |  | 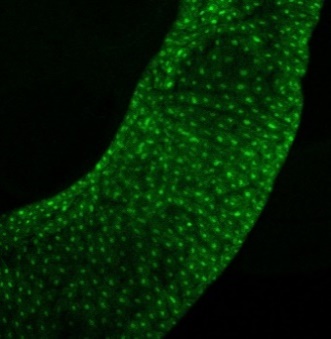 | 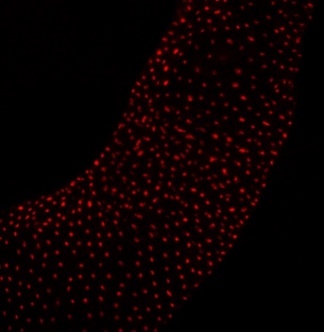 | 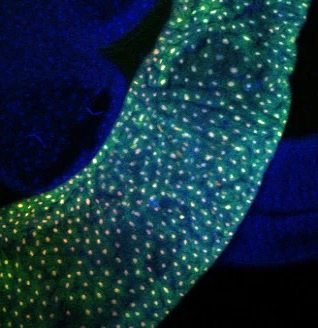 |
| **14**  *dMyc-Gal4*  *(BL47844)*  egg chamber |  |  |  | 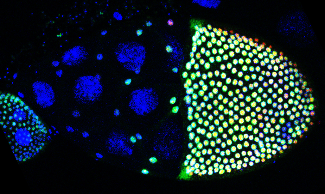 |
| **15**  *esg-Gal4*  adult midgut |  |  |  | 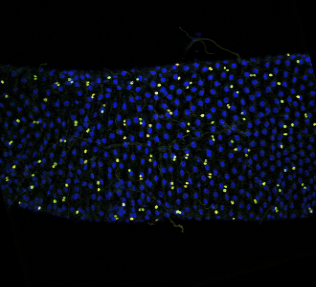 |

**Fig.3** GFP and RFP present different cellular localization. Examples in which is clearer the nuclear localization of RFP and both cytoplasmic and nuclear of GFP. Numbers indicate exposure times (in fractions of second).
