# Supplementary material for: In vivo study of gene expression with an enhanced dual-color fluorescent transcriptional timer
Source: eLife. 2019 May 29;8:e46181. doi: 10.7554/eLife.46181 (PMC6660218; doi:10.7554/eLife.46181)
Supplement: Supplementary file 3. [file elife-46181-supp3.docx]

|  | **DAPI** | **GFP** | **RFP** | **Overlay** |
| --- | --- | --- | --- | --- |
| **1**  *Atg18b^dG2AR^*  */CG8677^dG2AR^* | 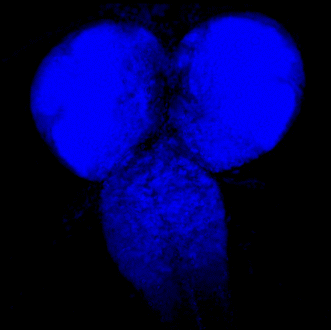 | 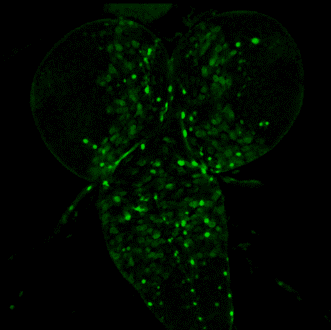 | 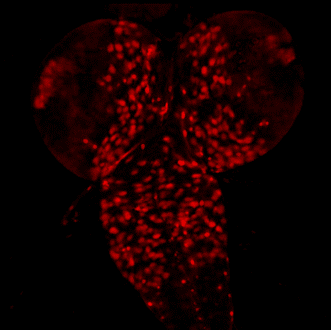 | 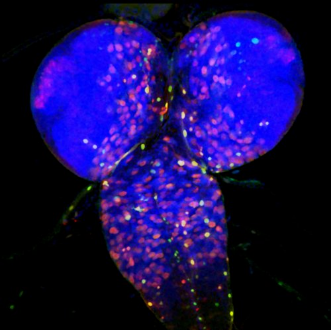 |
| **2**  *CaM^dG2AR^* | 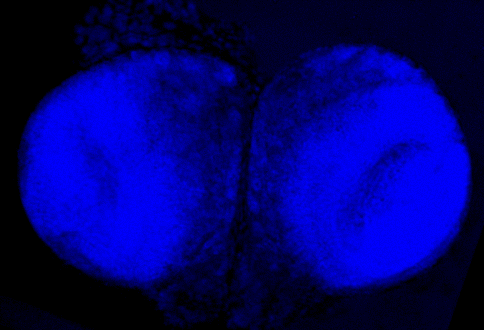 | 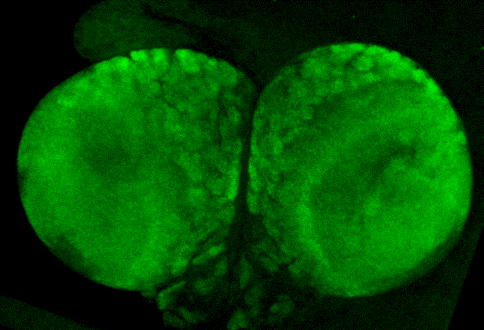 | 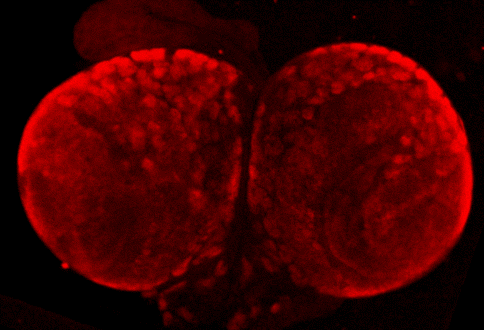 | 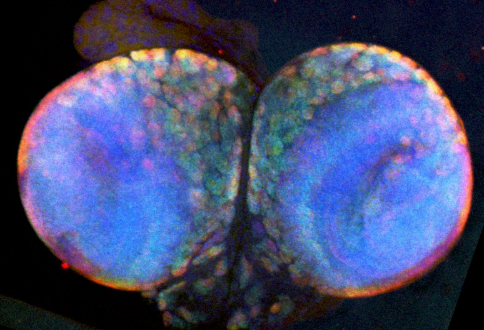 |
| **3**  *CaM^dG2AR^* | 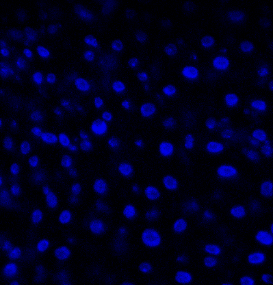 | 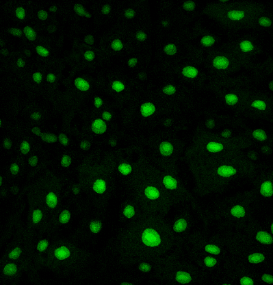 | 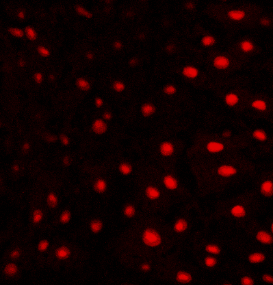 | 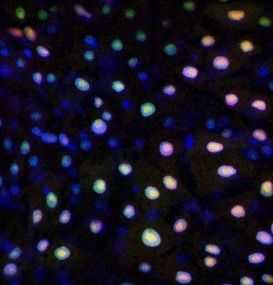 |
| **4**  *CG32795^dG2AR^* | 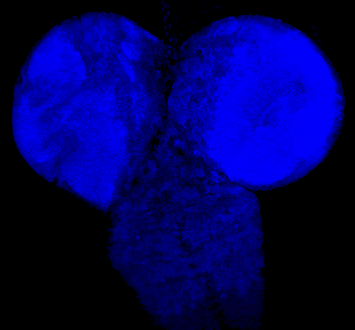 | 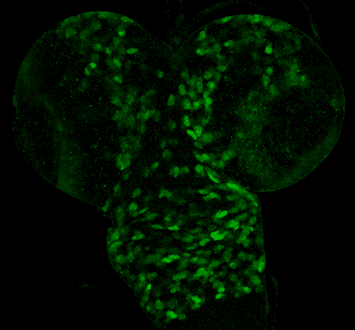 | 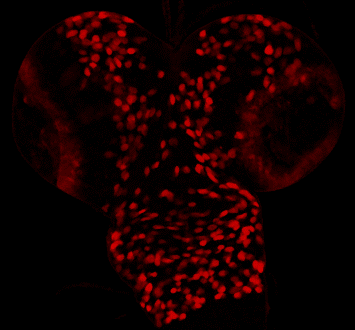 | 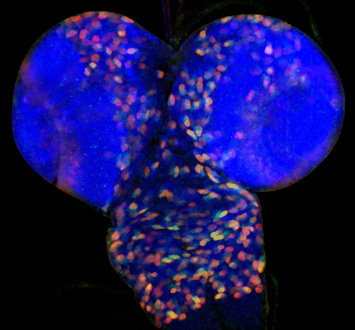 |
| **5**  *CG6650^dG2AR^*  */endos^dG2AR^* | 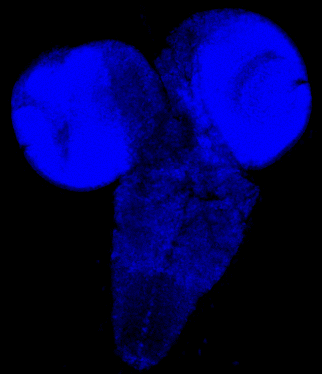 | 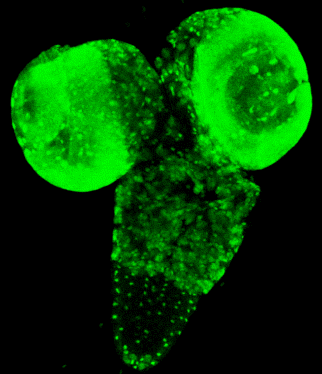 | 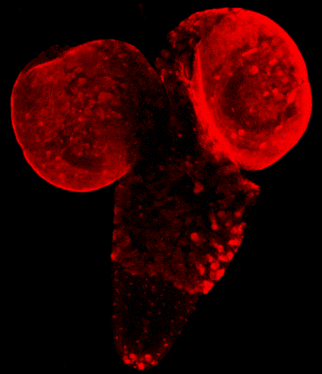 | 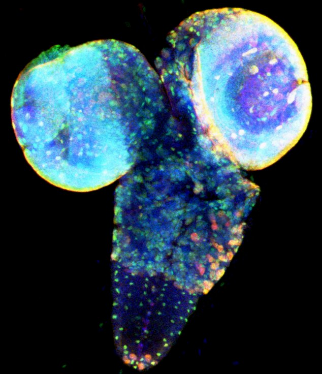 |
| **6**  *CG6650^dG2AR^*  */Endos^dG2AR^* | 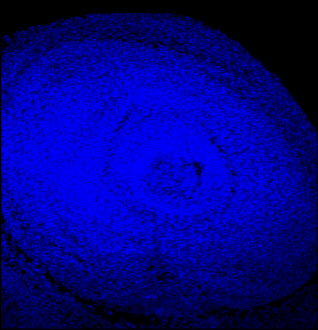 | 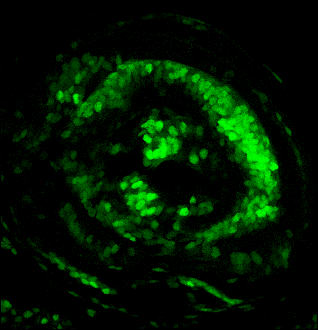 | 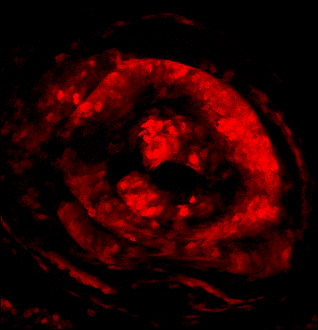 | 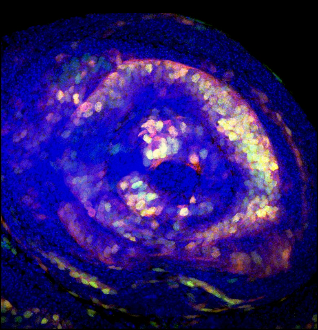 |
| **7**  *DopEcR^dG2AR^* | 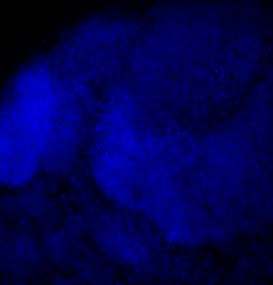 | 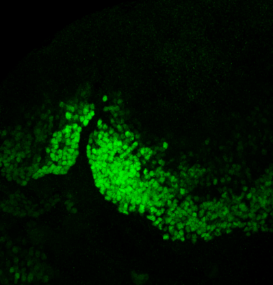 | 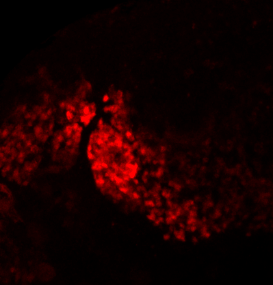 | 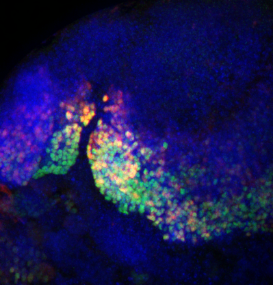 |
| **8**  *Lk6^dG2AR^* | 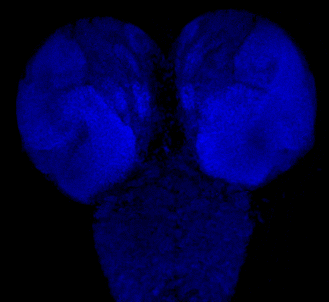 | 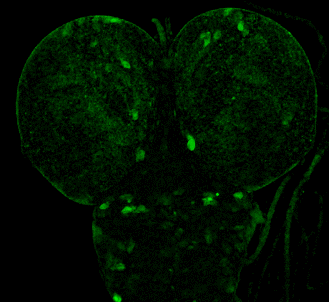 | 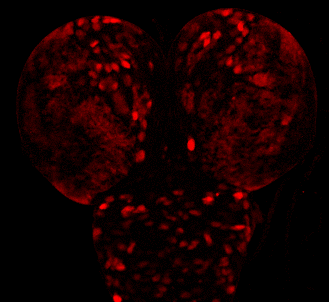 | 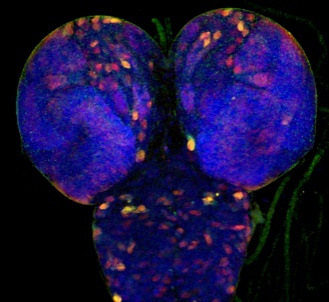 |
| **9**  *Qsm^dG2AR^* | 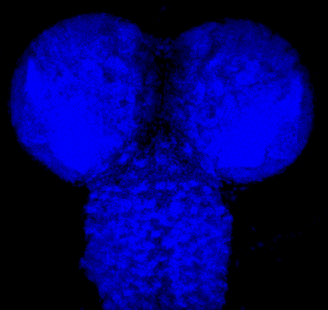 | 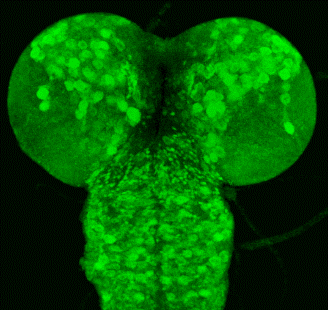 | 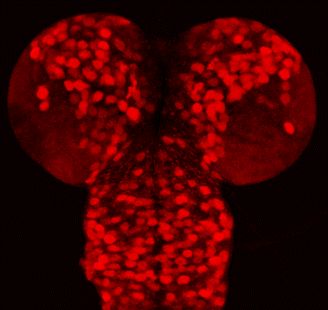 | 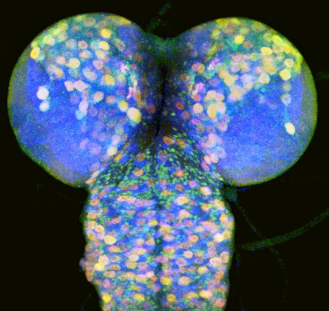 |
| **10**  ***Schi****zo^dG2AR^* | 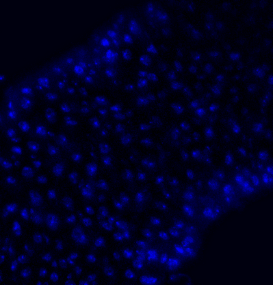 | 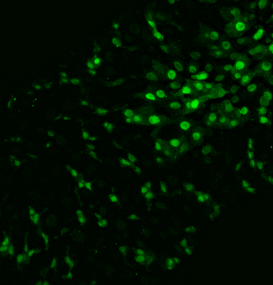 | 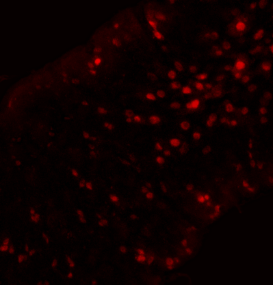 | 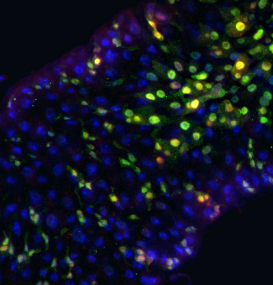 |
| **11**  *Tsp42Ea^dG2AR^/*  *CG30159^dG2AR^* | 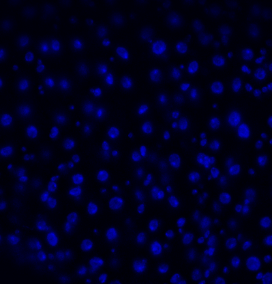 | 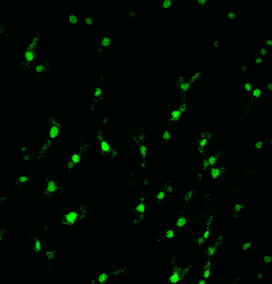 | 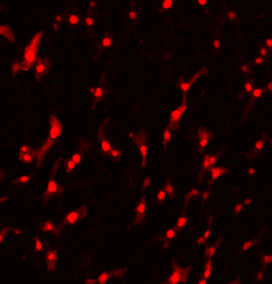 | 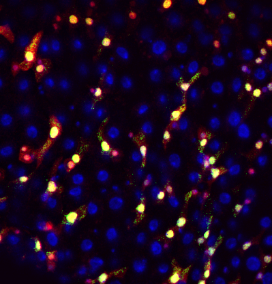 |
| **12**  *CrebA^dG2AR^* | 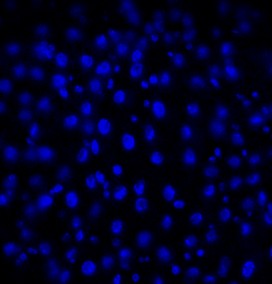 | 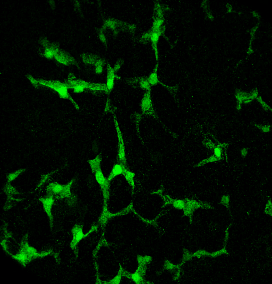 | 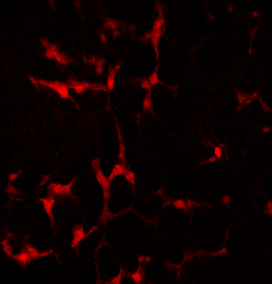 | 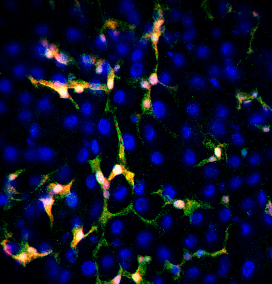 |
| **13**  *Gαo^dG2AR^* | 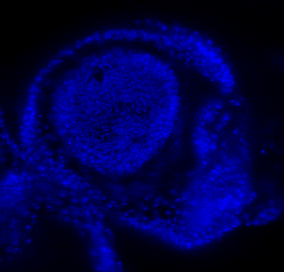 | 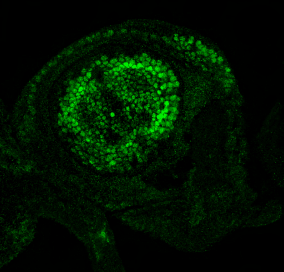 | 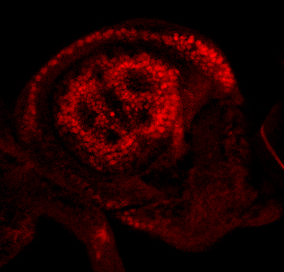 | 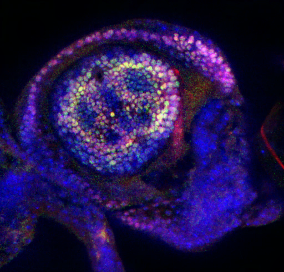 |
| **14**  *Inx2^dG2AR^* | 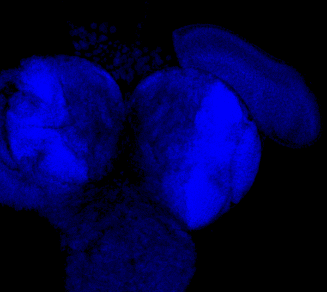 | 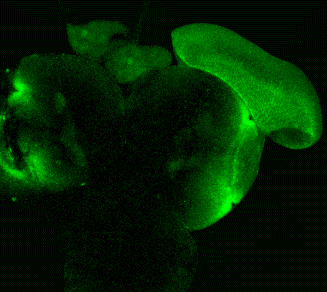 | 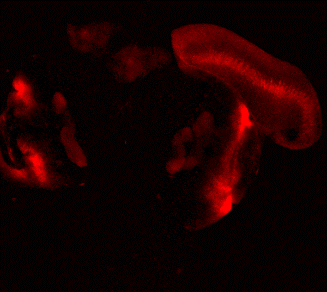 | 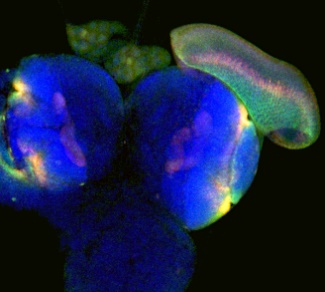 |
| **15**  *Lbk^dG2AR^*  */CG10731^dG2AR^* | 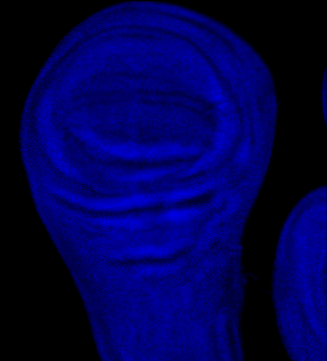 | 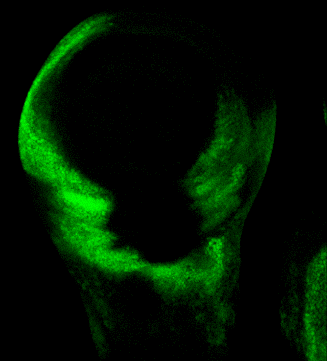 | 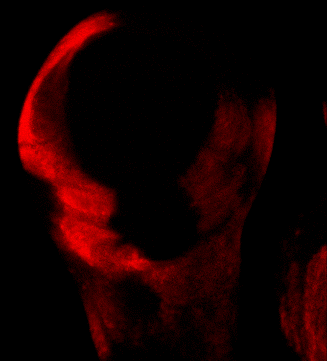 | 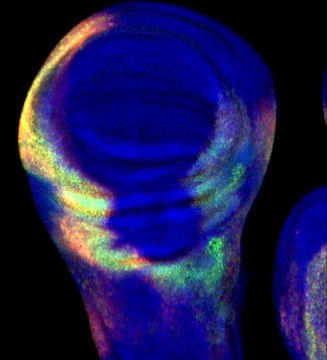 |
| **16**  *Sd^dG2AR^* | 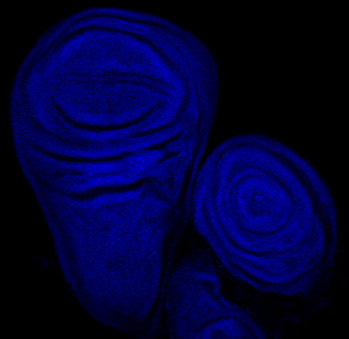 | 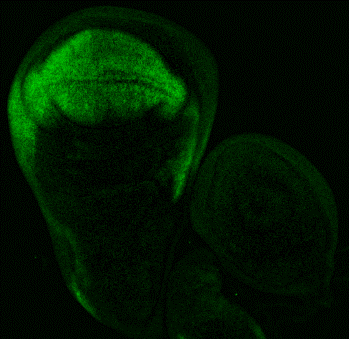 | 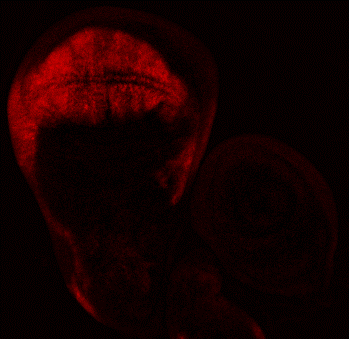 | 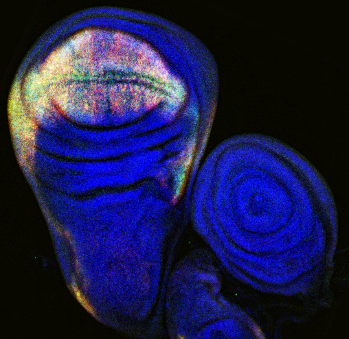 |
| **17**  *Ttk^dG2AR^* | 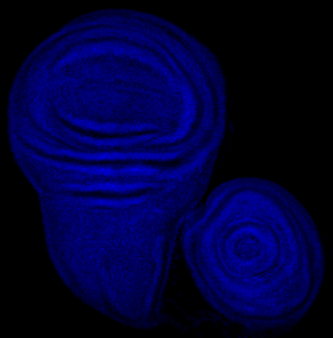 | 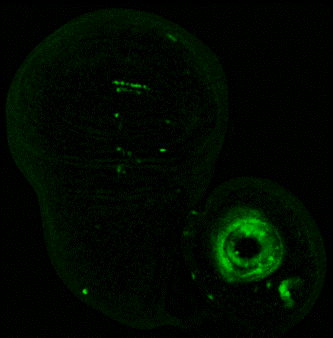 | 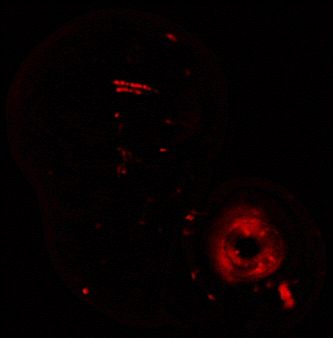 | 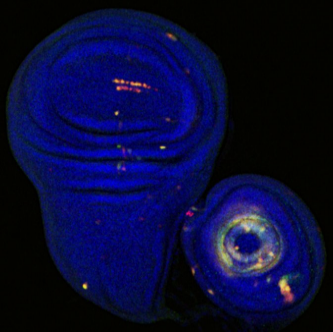 |
