# Supplementary material for: In vivo study of gene expression with an enhanced dual-color fluorescent transcriptional timer
Source: eLife. 2019 May 29;8:e46181. doi: 10.7554/eLife.46181 (PMC6660218; doi:10.7554/eLife.46181)
Supplement: Supplementary file 4. [file elife-46181-supp4.docx]

**Supplementary File 4.** **Cloning information and sequences of constructs.**

**List of plasmids generated:**


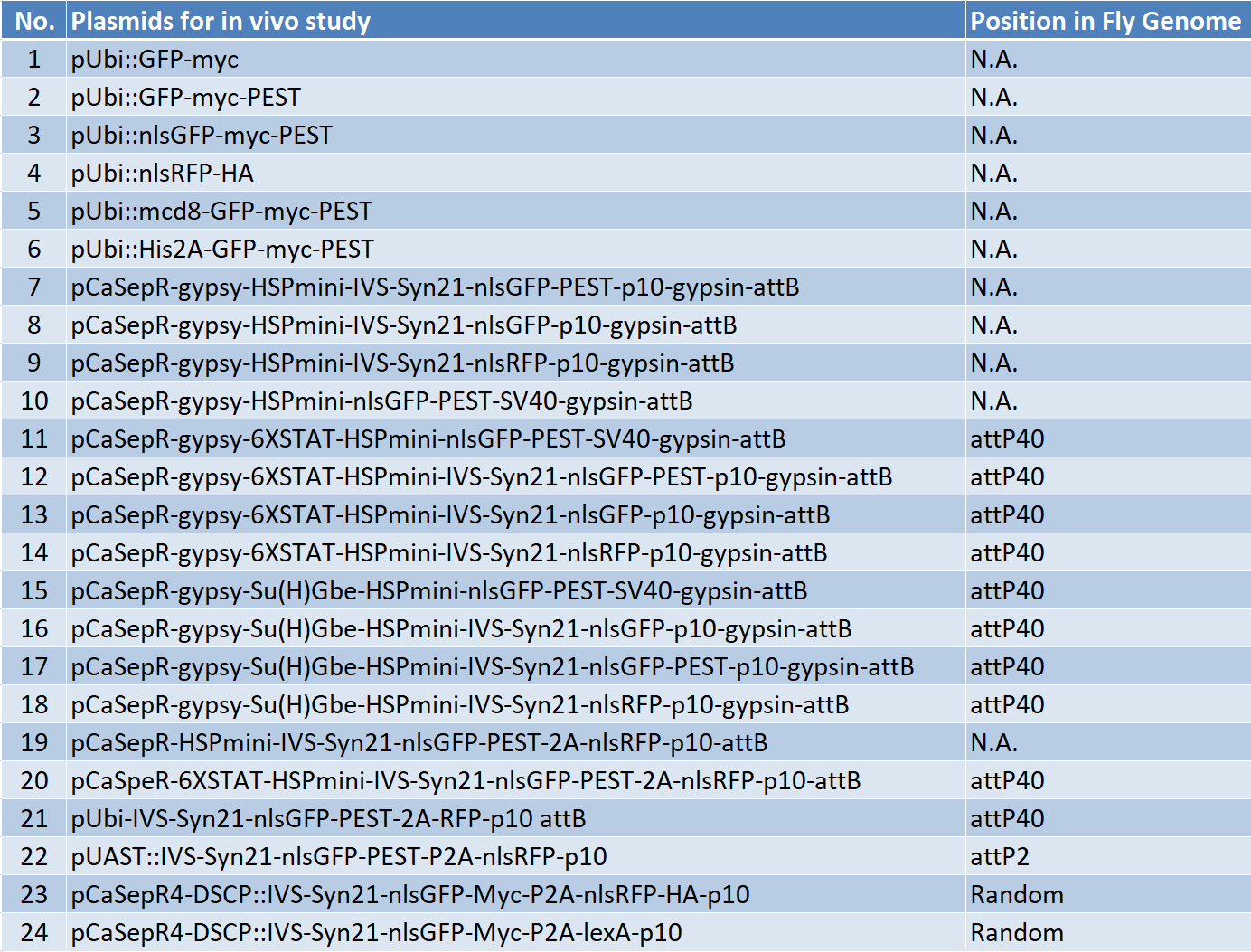


**Sequences of DNAs.**

**cytoGFP-Myc sequence:**

AAAATGGTGTCCAAGGGCGAGGAGCTGTTCACCGGCGTGGTGCCCATCCTGGTGGAGCTGGATGGCGACGTGAACGGCCACAAGTTCAGCGTGCGCGGCGAGGGCGAGGGCGACGCCACCAACGGCAAGCTGACCCTGAAGTTCATCTGCACCACCGGCAAGCTGCCCGTGCCCTGGCCCACCCTGGTGACCACCCTGACCTACGGCGTGCAGTGCTTCAGCCGCTACCCCGATCACATGAAGCAGCACGATTTCTTCAAGAGCGCCATGCCCGAGGGCTACGTGCAGGAGCGCACCATCAGCTTCAAGGATGACGGCACCTACAAGACCCGCGCCGAGGTGAAGTTCGAGGGCGATACCCTGGTGAACCGCATCGAGCTGAAGGGCATCGATTTCAAGGAGGATGGCAACATCCTGGGCCACAAGCTGGAGTACAACTTCAACAGCCACAACGTGTACATCACCGCCGATAAGCAGAAGAACGGCATCAAGGCCAACTTCAAGATCCGCCACAATGTGGAGGATGGCTCCGTGCAGCTGGCCGATCACTACCAGCAGAACACCCCCATCGGCGACGGCCCAGTGCTGCTGCCCGATAACCACTACCTGAGCACCCAGAGCGTGCTGTCCAAGGACCCCAACGAGAAGCGCGATCACATGGTGCTGCTGGAGTTCGTGACCGCCGCCGGCATCACCCTGGGCATGGATGAGCTGTACAAGACTAGTCTCGAGAACGGTGGTTCAGAGCAGAAGCTGATTAGCGAGGAGGACCTGGGCGAGCAGAAGCTGATTTCGGAGGAGGACCTCGGTGAGCAGAAGCTGATTTCCGAAGAGGACCTGAGCTAA

**cytoGFP-Myc-PEST sequence:**

AAAATGGTGTCCAAGGGCGAGGAGCTGTTCACCGGCGTGGTGCCCATCCTGGTGGAGCTGGATGGCGACGTGAACGGCCACAAGTTCAGCGTGCGCGGCGAGGGCGAGGGCGACGCCACCAACGGCAAGCTGACCCTGAAGTTCATCTGCACCACCGGCAAGCTGCCCGTGCCCTGGCCCACCCTGGTGACCACCCTGACCTACGGCGTGCAGTGCTTCAGCCGCTACCCCGATCACATGAAGCAGCACGATTTCTTCAAGAGCGCCATGCCCGAGGGCTACGTGCAGGAGCGCACCATCAGCTTCAAGGATGACGGCACCTACAAGACCCGCGCCGAGGTGAAGTTCGAGGGCGATACCCTGGTGAACCGCATCGAGCTGAAGGGCATCGATTTCAAGGAGGATGGCAACATCCTGGGCCACAAGCTGGAGTACAACTTCAACAGCCACAACGTGTACATCACCGCCGATAAGCAGAAGAACGGCATCAAGGCCAACTTCAAGATCCGCCACAATGTGGAGGATGGCTCCGTGCAGCTGGCCGATCACTACCAGCAGAACACCCCCATCGGCGACGGCCCAGTGCTGCTGCCCGATAACCACTACCTGAGCACCCAGAGCGTGCTGTCCAAGGACCCCAACGAGAAGCGCGATCACATGGTGCTGCTGGAGTTCGTGACCGCCGCCGGCATCACCCTGGGCATGGATGAGCTGTACAAGACTAGTCTCGAGAACGGTGGTTCAGAGCAGAAGCTGATTAGCGAGGAGGACCTGGGCGAGCAGAAGCTGATTTCGGAGGAGGACCTCGGTGAGCAGAAGCTGATTTCCGAAGAGGACCTGAGCGGCTCCGGACACGGTTTCCCCCCAGAGGTGGAGGAGCAGGACGATGGTACCCTGCCCATGTCGTGCGCCCAGGAATCCGGCATGGACCGTCACCCAGCCGCCTGCGCCTCCGCTCGCATTAACGTCGGCTCCGGAACCGGTTCCGGTTAA

**nlsGFP-Myc-PEST sequence:**

AAAATGCCCAAGAAGAAGCGCAAGGTGAGATCCCGTAAGTACACCTCGTGGTACGTCGCCCTGAAGCGCGTGTCCAAGGGCGAGGAGCTGTTCACCGGCGTGGTGCCCATCCTGGTGGAGCTGGATGGCGACGTGAACGGCCACAAGTTCAGCGTGCGCGGCGAGGGCGAGGGCGACGCCACCAACGGCAAGCTGACCCTGAAGTTCATCTGCACCACCGGCAAGCTGCCCGTGCCCTGGCCCACCCTGGTGACCACCCTGACCTACGGCGTGCAGTGCTTCAGCCGCTACCCCGATCACATGAAGCAGCACGATTTCTTCAAGAGCGCCATGCCCGAGGGCTACGTGCAGGAGCGCACCATCAGCTTCAAGGATGACGGCACCTACAAGACCCGCGCCGAGGTGAAGTTCGAGGGCGATACCCTGGTGAACCGCATCGAGCTGAAGGGCATCGATTTCAAGGAGGATGGCAACATCCTGGGCCACAAGCTGGAGTACAACTTCAACAGCCACAACGTGTACATCACCGCCGATAAGCAGAAGAACGGCATCAAGGCCAACTTCAAGATCCGCCACAATGTGGAGGATGGCTCCGTGCAGCTGGCCGATCACTACCAGCAGAACACCCCCATCGGCGACGGCCCAGTGCTGCTGCCCGATAACCACTACCTGAGCACCCAGAGCGTGCTGTCCAAGGACCCCAACGAGAAGCGCGATCACATGGTGCTGCTGGAGTTCGTGACCGCCGCCGGCATCACCCTGGGCATGGATGAGCTGTACAAGACTAGTCTCGAGAACGGTGGTTCAGAGCAGAAGCTGATTAGCGAGGAGGACCTGGGCGAGCAGAAGCTGATTTCGGAGGAGGACCTCGGTGAGCAGAAGCTGATTTCCGAAGAGGACCTGAGCGGCTCCGGACACGGTTTCCCCCCAGAGGTGGAGGAGCAGGACGATGGTACCCTGCCCATGTCGTGCGCCCAGGAATCCGGCATGGACCGTCACCCAGCCGCCTGCGCCTCCGCTCGCATTAACGTCGGCTCCTAA

**nlsRFP-HA insert sequence:**

ATGCCCAAGAAGAAGCGCAAGGTGCGCTCCCGTAAGTACACCTCCTGGTACGTCGCCCTGAAGCGCGTGTCCAAGGGCGAGGAGCTGATCAAGGAGAACATGCACATGAAGCTGTACATGGAGGGCACCGTGAACAACCACCACTTCAAGTGCACCAGCGAGGGCGAGGGCAAGCCCTACGAGGGCACCCAGACCATGCGCATCAAGGTGGTGGAGGGCGGCCCACTGCCCTTCGCCTTCGATATCCTGGCCACCTCCTTCATGTACGGCAGCCGCACCTTCATCAACCACACCCAGGGCATCCCCGATTTCTTCAAGCAGAGCTTCCCCGAGGGCTTCACCTGGGAGCGCGTGACCACCTACGAGGATGGCGGCGTGCTGACCGCCACCCAGGATACCAGCCTGCAGGATGGCTGCCTGATCTACAACGTGAAGATCCGCGGCGTGAACTTCCCCAGCAACGGCCCCGTGATGCAGAAGAAGACCCTGGGCTGGGAGGCCAACACCGAGATGCTGTACCCCGCCGATGGCGGCCTGGAGGGCCGCACCGATATGGCCCTGAAGCTGGTCGGCGGCGGCCACCTGATCTGCAACTTCAAGACCACCTACCGCAGCAAGAAGCCCGCCAAGAACCTGAAGATGCCCGGCGTGTACTACGTGGATCACCGCCTGGAGCGCATTAAGGAGGCCGATAAGGAGACCTACGTCGAGCAGCACGAGGTGGCCGTGGCCCGCTACTGCGATCTGCCGTCCAAGCTGGGACACAAGCTGAACGGCATGGATGAGCTGTACAAGACTAGTCAGAGTGGAAGCTACCCCTACGATGTTCCCGATTACGCTGGCTACCCCTACGATGTCCCCGATTACGCCGGATCCTACCCCTATGATGTCCCCGACTACGCCTAAGAG

**His2A-GFP-Myc-PEST sequence:**

ATGTCTGGACGTGGAAAAGGTGGCAAAGTGAAGGGAAAGGCAAAGTCCCGCTCAAACCGTGCCGGTCTTCAATTCCCTGTGGGCCGTATTCACCGTTTGCTCCGGAAGGGAAACTACGCAGAGCGTGTTGGTGCAGGCGCTCCAGTTTACCTAGCTGCCGTAATGGAATATCTGGCCGCTGAGGTTCTCGAGTTGGCTGGCAATGCTGCTCGTGACAACAAGAAGACTAGAATTATTCCGCGTCATCTGCAACTGGCCATCCGCAACGACGAGGAGTTAAACAAGCTGCTCTCCGGCGTCACAATTGCACAAGGTGGCGTGTTGCCTAATATACAGGCTGTTCTGTTGCCCAAGAAGACCGAGAAGAAGGCCTACCCAGCTTTCTTGTACAAAGTGGTGATCAGTCCCGGAGGCGGAGGCGGACCCGTGTCCAAGGGCGAGGAGCTGTTCACCGGCGTGGTGCCCATCCTGGTGGAGCTGGATGGCGACGTGAACGGCCACAAGTTCAGCGTGCGCGGCGAGGGCGAGGGCGACGCCACCAACGGCAAGCTGACCCTGAAGTTCATCTGCACCACCGGCAAGCTGCCCGTGCCCTGGCCCACCCTGGTGACCACCCTGACCTACGGCGTGCAGTGCTTCAGCCGCTACCCCGATCACATGAAGCAGCACGATTTCTTCAAGAGCGCCATGCCCGAGGGCTACGTGCAGGAGCGCACCATCAGCTTCAAGGATGACGGCACCTACAAGACCCGCGCCGAGGTGAAGTTCGAGGGCGATACCCTGGTGAACCGCATCGAGCTGAAGGGCATCGATTTCAAGGAGGATGGCAACATCCTGGGCCACAAGCTGGAGTACAACTTCAACAGCCACAACGTGTACATCACCGCCGATAAGCAGAAGAACGGCATCAAGGCCAACTTCAAGATCCGCCACAATGTGGAGGATGGCTCCGTGCAGCTGGCCGATCACTACCAGCAGAACACCCCCATCGGCGACGGCCCAGTGCTGCTGCCCGATAACCACTACCTGAGCACCCAGAGCGTGCTGTCCAAGGACCCCAACGAGAAGCGCGATCACATGGTGCTGCTGGAGTTCGTGACCGCCGCCGGCATCACCCTGGGCATGGATGAGCTGTACAAGACTAGTCTCGAGAACGGTGGTTCAGAGCAGAAGCTGATTAGCGAGGAGGACCTGGGCGAGCAGAAGCTGATTTCGGAGGAGGACCTCGGTGAGCAGAAGCTGATTTCCGAAGAGGACCTGAGCGGCTCCGGACACGGTTTCCCCCCAGAGGTGGAGGAGCAGGACGATGGTACCCTGCCCATGTCGTGCGCCCAGGAATCCGGCATGGACCGTCACCCAGCCGCCTGCGCCTCCGCTCGCATTAACGTCGGCTCC

**mcd8-GFP-Myc-PEST sequence:**

ATGGCCTCACCGTTGACCCGCTTTCTGTCGCTGAACCTGCTGCTGCTGGGTGAGTCGATTATCCTGGGGAGTGGAGAAGCTAAGCCACAGGCACCCGGATCCGTGAGCCGATCCAGATCAGGAGGCGGTGCTAGCGGAGGCGGCAGCGGAGGTGGAGGCTCGGGAGGTGGTAGATCTGAACTCCGAATCTTTCCAAAGAAAATGGACGCCGAACTTGGTCAGAAGGTGGACCTGGTATGTGAAGTGTTGGGGTCCGTTTCGCAAGGATGCTCTTGGCTCTTCCAGAACTCCAGCTCCAAACTCCCCCAGCCCACCTTCGTTGTCTATATGGCTTCATCCCACAACAAGATAACGTGGGACGAGAAGCTGAATTCGTCGAAACTGTTTTCTGCCATGAGGGACACGAATAATAAGTACGTTCTCACCCTGAACAAGTTCAGCAAGGAAAACGAAGGCTACTATTTCTGCTCAGTCATCAGCAACTCGGTGATGTACTTCAGTTCTGTCGTGCCAGTCCTTCAGAAAGTGAACTCTACTACTACCAAGCCAGTGCTGCGAACTCCCTCACCTGTGCACCCTACCGGGACATCTCAGCCCCAGAGACCAGAAGATTGTCGGCCCCGTGGCTCAGTGAAGGGGACCGGATTGGACTTCGCCTGTGATATTTACATCTGGGCACCCTTGGCCGGAATCTGCGTGGCCCTTCTGCTGTCCTTGATCATCACTCTCATCTGCTACCACGTGTCCAAGGGCGAGGAGCTGTTCACCGGCGTGGTGCCCATCCTGGTGGAGCTGGATGGCGACGTGAACGGCCACAAGTTCAGCGTGCGCGGCGAGGGCGAGGGCGACGCCACCAACGGCAAGCTGACCCTGAAGTTCATCTGCACCACCGGCAAGCTGCCCGTGCCCTGGCCCACCCTGGTGACCACCCTGACCTACGGCGTGCAGTGCTTCAGCCGCTACCCCGATCACATGAAGCAGCACGATTTCTTCAAGAGCGCCATGCCCGAGGGCTACGTGCAGGAGCGCACCATCAGCTTCAAGGATGACGGCACCTACAAGACCCGCGCCGAGGTGAAGTTCGAGGGCGATACCCTGGTGAACCGCATCGAGCTGAAGGGCATCGATTTCAAGGAGGATGGCAACATCCTGGGCCACAAGCTGGAGTACAACTTCAACAGCCACAACGTGTACATCACCGCCGATAAGCAGAAGAACGGCATCAAGGCCAACTTCAAGATCCGCCACAATGTGGAGGATGGCTCCGTGCAGCTGGCCGATCACTACCAGCAGAACACCCCCATCGGCGACGGCCCAGTGCTGCTGCCCGATAACCACTACCTGAGCACCCAGAGCGTGCTGTCCAAGGACCCCAACGAGAAGCGCGATCACATGGTGCTGCTGGAGTTCGTGACCGCCGCCGGCATCACCCTGGGCATGGATGAGCTGTACAAGACTAGTCTCGAGAACGGTGGTTCAGAGCAGAAGCTGATTAGCGAGGAGGACCTGGGCGAGCAGAAGCTGATTTCGGAGGAGGACCTCGGTGAGCAGAAGCTGATTTCCGAAGAGGACCTGAGCGGCTCCGGACACGGTTTCCCCCCAGAGGTGGAGGAGCAGGACGATGGTACCCTGCCCATGTCGTGCGCCCAGGAATCCGGCATGGACCGTCACCCAGCCGCCTGCGCCTCCGCTCGCATTAACGTCGGCTCCTAA

**pCaSpeR-gypsy-HSPmini-IVS-syn21-nlsGFP-PEST-p10-gypsy-attB2**

AAGCTTGGGCTGCAGGTCGACCTCGAGGCCTCGAGTTAACGTTACGTTAACGTTAACGTTCGAGGTCGACTCTAGTTGGCCACGTAATAAGTGTGCGTTGAATTTATTCGCAAAAACATTGCATATTTTCGGCAAAGTAAAATTTTGTTGCATACCTTATCAAAAAATAAGTGCTGCATACTTTTTAGAGAAACCAAATAATTTTTTATTGCATACCCGTTTTTAATAAAATACATTGCATACCCTCTTTTAATAAAAAATATTGCATACTTTGACGAAACAAATTTTCGTTGCATACCCAATAAAAGATTATTATATTGCATACCCGTTTTTAATAAAATACATTGCATACCCTCTTTTAATAAARAATATTGCATACGTTGACGAAACAAATTTTCGTTGCATACCCAATAAAAGATTATTATATTGCATACCTTTTCTTGCCATACCATTTAGCCGATCAATTGTGCTCGGCAACAGTATATTTGTGGTGTGCCAACCAACAACTCTAGGATCCAGATCCACTAGGATCTAAAAGGTAGGTTCAACCACTGATGCCTAGGCACACCGAAACGACTAACCCTAATTCTTATCCTTTACTTCAGGCGGAACTTAAAAAAAAAAATCAAAATGCCCAAGAAGAAGCGCAAGGTGGTGTCCAAGGGCGAGGAGCTGTTCACCGGCGTGGTGCCCATCCTGGTGGAGCTGGATGGCGACGTGAACGGCCACAAGTTCAGCGTGCGCGGCGAGGGCGAGGGCGACGCCACCAACGGCAAGCTGACCCTGAAGTTCATCTGCACCACCGGCAAGCTGCCCGTGCCCTGGCCCACCCTGGTGACCACCCTGACCTACGGCGTGCAGTGCTTCAGCCGCTACCCCGATCACATGAAGCAGCACGATTTCTTCAAGAGCGCCATGCCCGAGGGCTACGTGCAGGAGCGCACCATCAGCTTCAAGGATGACGGCACCTACAAGACCCGCGCCGAGGTGAAGTTCGAGGGCGATACCCTGGTGAACCGCATCGAGCTGAAGGGCATCGATTTCAAGGAGGATGGCAACATCCTGGGCCACAAGCTGGAGTACAACTTCAACAGCCACAACGTGTACATCACCGCCGATAAGCAGAAGAACGGCATCAAGGCCAACTTCAAGATCCGCCACAATGTGGAGGATGGCTCCGTGCAGCTGGCCGATCACTACCAGCAGAACACCCCCATCGGCGACGGCCCAGTGCTGCTGCCCGATAACCACTACCTGAGCACCCAGAGCGTGCTGTCCAAGGACCCCAACGAGAAGCGCGATCACATGGTGCTGCTGGAGTTCGTGACCGCCGCCGGCATCACCCTGGGCATGGATGAGCTGTACAAGACTAGTTACAAGAAGCTTAGCCATGGCTTCCCGCCGGAGGTGGAGGAGCAGGATGATGGCACGCTGCCCATGTCTTGTGCCCAGGAGAGCGGGATGGACCGTCACCCTGCAGCCTGTGCTTCTGCTAGGATCAATGTGTAGATGCGCCTAGTTAATAGAATGAATCGTTTTTAAAATAACAAATCAATTGTTTTATAATATTCGTACGATTCTTTGATTATGTAATAAAATGTGATCATTAGGAAGATTACGAAAAATATAAAAAATATGAGTTCTGTGTGTATAACAAATGCTGTAAACGCCACAATTGTGTTTGTTGCAAATAAACCCATGATTATTTGATTAAAATTGTTGTTTTCTTTGTTCATAGACAATAGTGTGTTTTGCCTAAACGTGTACTGCATAAACTCCATGCGAGTGTATAGCGAGCTAGTGGCTAACGCTTGCCCCACCAAAGTAGATTCGTCAAAATCCTCAATTTCATCACCCTCCTCCAAGTTTAACATTTGGCCGTCGGAATTAACTTCTAAAGATGCCACATAATCTAATAAATGAAATAGAGATTCAAACGTGGCGTCATCGTCCGTTTCGACCATTTCCGAAAAGAACTCGGGCATAAACTCTATGATTTCTCTGGACGTGGTGTTGTCGAAACTCTCAAAGTACGCAGTCAGGAACGTGCGCGACATGTCGTCGGGAAACTCGCGCGGAAACATGTTGTTGTAACCGAACGGGTCCCATAGCGCCAAAACCAAATCTGCCAGCGTCAATAGAATGAGCACGATGCCGACAATGGAGCTGGCTTGGATAGCGATTCGAGTTAACGGCCGGCCGCTAGTTGGCCACGTAATAAGTGTGCGTTGAATTTATTCGCAAAAACATTGCATATTTTCGGCAAAGTAAAATTTTGTTGCATACCTTATCAAAAAATAAGTGCTGCATACTTTTTAGAGAAACCAAATAATTTTTTATTGCATACCCGTTTTTAATAAAATACATTGCATACCCTCTTTTAATAAAAAATATTGCATACTTTGACGAAACAAATTTTCGTTGCATACCCAATAAAAGATTATTATATTGCATACCCGTTTTTAATAAAATACATTGCATACCCTCTTTTAATAAARAATATTGCATACGTTGACGAAACAAATTTTCGTTGCATACCCAATAAAAGATTATTATATTGCATACCTTTTCTTGCCATACCATTTAGCCGATCAATTGTGCTCGGCAACAGTATATTTGTGGTGTGCCAACCAACAACTCTAGTGCTGCATCCAACGCGTTGGGAGCTCTCCGGATCAATTCGGCTTCAGGTACCGTCGACGATGTAGGTCACGGTCTCGAAGCCGCGGTGCGGGTGCCAGGGCGTGCCCTTGGGCTCCCCGGGCGCGTACTCCACCTCACCCATCTGGTCCATCATGATGAACGGGTCGAGGTGGCGGTAGTTGATCCCGGCGAACGCGCGGCGCACCGGGAAGCCCTCGCCCTCGAAACCGCTGGGCGCGGTGGTCACGGTGAGCACGGGACGTGCGACGGCGTCGGCGGGTGCGGATACGCGGGGCAGCGTCAGCGGGTTCTCGACGGTCACGGCGGGCATGTCGACAAGCCGAATTGATCCACTAGAAGGCCTAATTCGCGGCCGCGGATCTGATCCCGGGCGGGTACCGAATTCTAGTATGTATGTAAGTTAATAAAACCCTTTTTTGGAGAATGTAGATTTAAAAAAACATATTTTTTTTTTATTTTTTACTGCACTGGATATCATTGAACTTATCTGATCAGTTTTAAATTTACTTCGATCCAAGGGTATTTGAAGTACCAGGTTCTTTCGATTACCTCTCACTCAAAATGACATTCCACTCAAAGTCAGCGCTGTTTGCCTCCTTCTCTGTCCACAGAAATATCGCCGTCTCTTTCGCCGCTGCGTCCGCTATCTCTTTCGCCACCGTTTGTAGCGTTACCTAGCGTCAATGTCCGCCTTCAGTTGCACTTTGTCAGCGGTTTCGTGACGAAGCTCCAAGCGGTTTACGCCATCAATTAAACACAAAGTGCTGTGCCAAAACTCCTCTCGCTTCTTATTTTTGTTTGTTTTTTGAGTGATTGGGGTGGTGATTGGTTTTGGGTGGGTAAGCAGGGGAAAGTGTGAAAAATCCCGGCAATGGGCCAAGAGGATCAGGAGCTATTAATTCGCGGAGGCAGCAAACACCCATCTGCCGAGCATCTGAACAATGTGAGTAGTACATGTGCATACATCTTAAGTTCACTTGATCTATAGGAACTGCGATTGCAACATCAAATTGTCTGCGGCGTGAGAACTGCGACCCACAAAAATCCCAAACCGCAATCGCACAAACAAATAGTGACACGAAACAGATTATTCTGGTAGCTGTGCTCGCTATATAAGACAATTTTTAAGATCATATCATGATCAAGACATCTAAAGGCATTCATTTTCGACTACATTCTTTTTTACAAAAAATATAACAACCAGATATTTTAAGCTGATCCTAGATGCACAAAAAATAAATAAAAGTATAAACCTACTTCGTAGGATACTTCGTTTTGTTCGGGGTTAGATGAGCATAACGCTTGTAGTTGATATTTGAGATCCCCTATCATTGCAGGGTGACAGCGGACGCTTCGCAGAGCTGCATTAACCAGGGCTTCGGGCAGGCCAAAAACTACGGCACGCTCCTGCCACCCAGTCCGCCGGAGGACTCCGGTTCAGGGAGCGGCCAACTAGCCGAGAACCTCACCTATGCCTGGCACAATATGGACATCTTTGGGGCGGTCAATCAGCCGGGCTCCGGATGGCGGCAGCTGGTCAACCGGACACGCGGACTATTCTGCAACGAGCGACACATACCGGCGCCCAGGAAACATTTGCTCAAGAACGGTGAGTTTCTATTCGCAGTCGGCTGATCTGTGTGAAATCTTAATAAAGGGTCCAATTACCAATTTGAAACTCAGTTTGCGGCGTGGCCTATCCGGGCGAACTTTTGGCCGTGATGGGCAGTTCCGGTGCCGGAAAGACGACCCTGCTGAATGCCCTTGCCTTTCGATCGCCGCAGGGCATCCAAGTATCGCCATCCGGGATGCGACTGCTCAATGGCCAACCTGTGGACGCCAAGGAGATGCAGGCCAGGTGCGCCTATGTCCAGCAGGATGACCTCTTTATCGGCTCCCTAACGGCCAGGGAACACCTGATTTTCCAGGCCATGGTGCGGATGCCACGACATCTGACCTATCGGCAGCGAGTGGCCCGCGTGGATCAGGTGATCCAGGAGCTTTCGCTCAGCAAATGTCAGCACACGATCATCGGTGTGCCCGGCAGGGTGAAAGGTCTGTCCGGCGGAGAAAGGAAGCGTCTGGCATTCGCCTCCGAGGCACTAACCGATCCGCCGCTTCTGATCTGCGATGAGCCCACCTCCGGACTGGACTCATTTACCGCCCACAGCGTCGTCCAGGTGCTGAAGAAGCTGTCGCAGAAGGGCAAGACCGTCATCCTGACCATTCATCAGCCGTCTTCCGAGCTGTTTGAGCTCTTTGACAAGATCCTTCTGATGGCCGAGGGCAGGGTAGCTTTCTTGGGCACTCCCAGCGAAGCCGTCGACTTCTTTTCCTAGTGAGTTCGATGTGTTTATTAAGGGTATCTAGCATTACATTACATCTCAACTCCTATCCAGCGTGGGTGCCCAGTGTCCTACCAACTACAATCCGGCGGACTTTTACGTACAGGTGTTGGCCGTTGTGCCCGGACGGGAGATCGAGTCCCGTGATCGGATCGCCAAGATATGGCGACAATTTTGCTATTAGCAAAGTAGCCCGGGATATGGAGCAGTTGTTGGCCACCAAAAATTTGGAGAAGCCACTGGAGCAGCCGGAGAATGGGTACACCTACAAGGCCACCTGGTTCATGCAGTTCCGGGCGGTCCTGTGGCGATCCTGGCTGTCGGTGCTCAAGGAACCACTCCTCGTAAAAGTGCGACTTATTCAGACAACGGTGAGTGGTTCCAGTGGAAACAAATGATATAACGCTTACAATTCTTGGAAACAAATTCGCTAGATTTTAGTTAGAATTGCCTGATTCCACACCCTTCTTAGTTTTTTTCAATGAGATGTATAGTTTATAGTTTTGCAGAAAATAAATAAATTTCATTTAACTCGCGAACATGTTGAAGATATGAATATTAATGAGATGCGAGTAACATTTTAATTTGCAGATGGTTGCCATCTTGATTGGCCTCATCTTTTTGGGCCAACAACTCACGCAAGTGGGCGTGATGAATATCAACGGAGCCATCTTCCTCTTCCTGACCAACATGACCTTTCAAAACGTCTTTGCCACGATAAATGTAAGTCTTGTTTAGAATACATTTGCATATTAATAATTTACTAACTTTCTAATGAATCGATTCGATTTAGGTGTTCACCTCAGAGCTGCCAGTTTTTATGAGGGAGGCCCGAAGTCGACTTTATCGCTGTGACACATACTTTCTGGGCAAAACGATTGCCGAATTACCGCTTTTTCTCACAGTGCCACTGGTCTTCACGGCGATTGCCTATCCGATGATCGGACTGCGGGCCGGAGTGCTGCACTTCTTCAACTGCCTGGCGCTGGTCACTCTGGTGGCCAATGTGTCAACGTCCTTCGGATATCTAATATCCTGCGCCAGCTCCTCGACCTCGATGGCGCTGTCTGTGGGTCCGCCGGTTATCATACCATTCCTGCTCTTTGGCGGCTTCTTCTTGAACTCGGGCTCGGTGCCAGTATACCTCAAATGGTTGTCGTACCTCTCATGGTTCCGTTACGCCAACGAGGGTCTGCTGATTAACCAATGGGCGGACGTGGAGCCGGGCGAAATTAGCTGCACATCGTCGAACACCACGTGCCCCAGTTCGGGCAAGGTCATCCTGGAGACGCTTAACTTCTCCGCCGCCGATCTGCCGCTGGACTACGTGGGTCTGGCCATTCTCATCGTGAGCTTCCGGGTGCTCGCATATCTGGCTCTAAGACTTCGGGCCCGACGCAAGGAGTAGCCGACATATATCCGAAATAACTGCTTGTTTTTTTTTTTACCATTATTACCATCGTGTTTACTGTTTATTGCCCCCTCAAAAAGCTAATGTAATTATATTTGTGCCAATAAAAACAAGATATGACCTATAGAATACAAGTATTTCCCCTTCGAACATCCCCACAAGTAGACTTTGGATTTGTCTTCTAACCAAAAGACTTACACACCTGCATACCTTACATCAAAAACTCGTTTATCGCTACATAAAACACCGGGATATATTTTTTATATACATACTTTTCAAATCGCGCGCCCTCTTCATAATTCACCTCCACCACACCACGTTTCGTAGTTGCTCTTTCGCTGTCTCCCACCCGCTCTCCGCAACACATTCACCTTTTGTTCGACGACCTTGGAGCGACTGTCGTTAGTTCCGCGCGATTCGGTTCGCTCAAATGGTTCCGAGTGGTTCATTTCGTCTCAATAGAAATTAGTAATAAATATTTGTATGTACAATTTATTTGCTCCAATATATTTGTATATATTTCCCTCACAGCTATATTTATTCTAATTTAATATTATGACTTTTTAAGGTAATTTTTTGTGACCTGTTCGGAGTGATTAGCGTTACAATTTGAACTGAAAGTGACATCCAGTGTTTGTTCCTTGTGTAGATGCATCTCAAAAAAATGGTGGGCATAATAGTGTTGTTTATATATATCAAAAATAACAACTATAATAATAAGAATACATTTAATTTAGAAAATGCTTGGATTTCACTGGAACTAGAATTAATTCGGCTGCTGCTCTAAACGACGCATTTCGTACTCCAAAGTACGAATTTTTTCCCTCAAGCTCTTATTTTCATTAAACAATGAACAGGACCTAACGCACAGTCACGTTATTGTTTACATAAATGATTTTTTTTACTATTCAAACTTACTCTGTTTGTGTACTCCCACTGGTATAGCCTTCTTTTATCTTTTCTGGTTCAGGCTCTATCACTTTACTAGGTACGGCATCTGCGTTGAGTCGCCTCCTTTTAAATGTCTGACCTTTTGCAGGTGCAGCCTTCCACTGCGAATCTTTAAAGTGGGTATCACAAATTTGGGAGTTTTCACCAAGGCTGCACCCAAGGCTCTGCTCCCACAATTTTCTCTTAATAGCACACTTCGGCACGTGAATTAATTTTACTCCAGTCACAGCTTTGCAGCAAAATTTGCAATATTTCATTTTTTTTTATTCCACGTAAGGGTTAATGTTTTCAAAAAAAAATTCGTCCGCACACAACCTTTCCTCTCAACAAGCAAACGTGCACTGAATTTAAGTGTATACTTCGGTAAGCTTCGGCTATCGACGGGACCACCTTATGTTATTTCATCATGGGCCAGACCCACGTAGTCCAGCGGCAGATCGGCGGCGGAGAAGTTAAGCGTCTCCAGGATGACCTTGCCCGAACTGGGGCACGTGGTGTTCGACGATGTGCAGCTAATTTCGCCCGGCTCCACGTCCGCCCATTGGTTAATCAGCAGACCCTCGTTGGCGTAACGGAACCATGAGAGGTACGACAACCATTTGAGGTATACTGGCACCGAGCCCGAGTTCAAGAAGAAGGCGTTTTTCCATAGGCTCCGCCCCCCTGACGAGCATCACAAAAATCGACGCTCAAGTCAGAGGTGGCGAAACCCGACAGGACTATAAAGATACCAGGCGTTTCCCCCTGGAAGCTCCCTCGTGCGCTCTCCTGTTCCGACCCTGCCGCTTACCGGATACCTGTCCGCCTTTCTCCCTTCGGGAAGCGTGGCGCTTTCTCAATGCTCACGCTGTAGGTATCTCAGTTCGGTGTAGGTCGTTCGCTCCAAGCTGGGCTGTGTGCACGAACCCCCCGTTCAGCCCGACCGCTGCGCCTTATCCGGTAACTATCGTCTTGAGTCCAACCCGGTAAGACACGACTTATCGCCACTGGCAGCAGCCACTGGTAACAGGATTAGCAGAGCGAGGTATGTAGGCGGTGCTACAGAGTTCTTGAAGTGGTGGCCTAACTACGGCTACACTAGAAGGACAGTATTTGGTATCTGCGCTCTGCTGAAGCCAGTTACCTTCGGAAAAAGAGTTGGTAGCTCTTGATCCGGCAAACAAACCACCGCTGGTAGCGGTGGTTTTTTTGTTTGCAAGCAGCAGATTACGCGCAGAAAAAAAGGATCTCAAGAAGATCCTTTGATCTTTTCTACGGGGTCTGACGCTCAGTGGAACGAAAACTCACGTTAAGGGATTTTGGTCATGAGATTATCAAAAAGGATCTTCACCTAGATCCTTTTAAATTAAAAATGAAGTTTTAAATCAATCTAAAGTATATATGAGTAAACTTGGTCTGACAGTTACCAATGCTTAATCAGTGAGGCACCTATCTCAGCGATCTGTCTATTTCGTTCATCCATAGTTGCCTGACTCCCCGTCGTGTAGATAACTACGATACGGGAGGGCTTACCATCTGGCCCCAGTGCTGCAATGATACCGCGAGACCCACGCTCACCGGCTCCAGATTTATCAGCAATAAACCAGCCAGCCGGAAGGGCCGAGCGCAGAAGTGGTCCTGCAACTTTATCCGCCTCCATCCAGTCTATTAATTGTTGCCGGGAAGCTAGAGTAAGTAGTTCGCCAGTTAATAGTTTGCGCAACGTTGTTGCCATTGCTACAGGCATCGTGGTGTCACGCTCGTCGTTTGGTATGGCTTCATTCAGCTCCGGTTCCCAACGATCAAGGCGAGTTACATGATCCCCCATGTTGTGCAAAAAAGCGGTTAGCTCCTTCGGTCCTCCGATCGTTGTCAGAAGTAAGTTGGCCGCAGTGTTATCACTCATGGTTATGGCAGCACTGCATAATTCTCTTACTGTCATGCCATCCGTAAGATGCTTTTCTGTGACTGGTGAGTACTCAACCAAGTCATTCTGAGAATAGTGTATGCGGCGACCGAGTTGCTCTTGCCCGGCGTCAATACGGGATAATACCGCGCCACATAGCAGAACTTTAAAAGTGCTCATCATTGGAAAACGTTCTTCGGGGCGAAAACTCTCAAGGATCTTACCGCTGTTGAGATCCAGTTCGATGTAACCCACTCGTGCACCCAACTGATCTTCAGCATCTTTTACTTTCACCAGCGTTTCTGGGTGAGCAAAAACAGGAAGGCAAAATGCCGCAAAAAAGGGAATAAGGGCGACACGGAAATGTTGAATACTCATACTCTTCCTTTTTCAATATTATTGAAGCATTTATCAGGGTTATTGTCTCATGAGCGGATACATATTTGAATGTATTTAGAAAAATAAACAAATAGGGGTTCCGCGCACATTTCCCCGAAAAGTGCCACCTGACGTCTAAGAAACCATTATTATCATGACATTAACCTATAAAAATAGGCGTATCACGAGGCCCTTTCGTCTCGCGCGTTTCGGTGATGACGGTGAAAACCTCTGACACATGCAGCTCCCGGAGACGGTCACAGCTTGTCTGTAAGCGGATGCCGGGAGCAGACAAGCCCGTCAGGGCGCGTCAGCGGGTGTTGGCGGGTGTCGGGGCTGGCTTAACTATGCGGCATCAGAGCAGATTGTACTGAGAGTGCACCATATGCGGTGTGAAATACCGCACCGAATCGCGCGGAACTAACGACAGTCGCTCCAAGGTCGTCGAACAAAAGGTGAATGTGTTGCGGAGAGCGGGTGGGAGACAGCGAAAGAGCAACTACGAAACGTGGTGTGGTGGAGGTGAATTATGAAGAGGGCGCGCGATTTGAAAAGTATGTATATAAAAAATATATCCCGGTGTTTTATGTAGCGATAAACGAGTTTTTGATGTAAGGTATGCAGGTGTGTAAGTCTTTTGGTTAGAAGACAAATCCAAAGTCTACTTGTGGGGATGTTCGAAGGGGAAATACTTGTATTCTATAGGTCATATCTTGTTTTTATTGGCACAAATATAATTACATTAGCTTTTTGAGGGGGCAATAAACAGTAAACACGATGGTAATAATGGTAAAAAAAAAAACAAGCAGTTATTTCGGATATATGTCGGCTACTCCTTGCGTCGGGCCCGAAGTCTTAGAGCCAGATATGCGAGCACCCGGAAGCTCACGATGAGAATGGCCAGACCATGATGAAATAACATAAGGTGGTCCCGTCGGCAAGAGACATCCACTTAACGTATGCTTGCAATAAGTGCGAGTGAAAGGAATAGTATTCTGAGTGTCGTATTGAGTCTGAGTGAGACAGCGATATGATTGTTGATTAACCCTTAGCATGTCCGTGGGGTTTGAATTAACTCATAATATTAATTAGACGAAATTATTTTTAAAGTTTTATTTTTAATAATTTGCGAGTACGCA

**pCaSpeR-gypsy-HSPmini-****IVS-syn21-nlsRFP-p10-gypsy-attB2**

AAGCTTGGGCTGCAGGTCGACCTCGAGGCCTCGAGTTAACGTTACGTTAACGTTAACGTTCGAGGTCGACTCTAGTTGGCCACGTAATAAGTGTGCGTTGAATTTATTCGCAAAAACATTGCATATTTTCGGCAAAGTAAAATTTTGTTGCATACCTTATCAAAAAATAAGTGCTGCATACTTTTTAGAGAAACCAAATAATTTTTTATTGCATACCCGTTTTTAATAAAATACATTGCATACCCTCTTTTAATAAAAAATATTGCATACTTTGACGAAACAAATTTTCGTTGCATACCCAATAAAAGATTATTATATTGCATACCCGTTTTTAATAAAATACATTGCATACCCTCTTTTAATAAARAATATTGCATACGTTGACGAAACAAATTTTCGTTGCATACCCAATAAAAGATTATTATATTGCATACCTTTTCTTGCCATACCATTTAGCCGATCAATTGTGCTCGGCAACAGTATATTTGTGGTGTGCCAACCAACAACTCTAGAGGATCCAGATCCACTAGCGAGCGGAGACTCTAGCGCTAGCGACGTCGAGCGCCGGAGTATAAATAGAGGCGCTTCGTCTACGGAGCGACAATTCAATTCAAACAAGCAAAGTGAACACGTCGCTAAGCGAAAGCTAAGCAAATAAACAAGCGCAGCTGAACAAGCTAAACAATCTGCAGTAAAGTGCAAGTTAAAGTGAATCAATTAAAAGTAACCAGCAACCAAGTAAATCAACTGCAACTACTGAAATCTGCCAAGAAGTAATTATTGAATACAAGAAGAGAACTCTGAATAGCGGCCGCGGCTCGAGGGTACCAACTTAAAAAAAAAAATCAAAATGCCCAAGAAGAAGCGCAAGGTGGTGTCCAAGGGCGAGGAGCTGATCAAGGAGAACATGCACATGAAGCTGTACATGGAGGGCACCGTGAACAACCACCACTTCAAGTGCACCAGCGAGGGCGAGGGCAAGCCCTACGAGGGCACCCAGACCATGCGCATCAAGGTGGTGGAGGGCGGCCCACTGCCCTTCGCCTTCGATATCCTGGCCACCTCCTTCATGTACGGCAGCCGCACCTTCATCAACCACACCCAGGGCATCCCCGATTTCTTCAAGCAGAGCTTCCCCGAGGGCTTCACCTGGGAGCGCGTGACCACCTACGAGGATGGCGGCGTGCTGACCGCCACCCAGGATACCAGCCTGCAGGATGGCTGCCTGATCTACAACGTGAAGATCCGCGGCGTGAACTTCCCCAGCAACGGCCCCGTGATGCAGAAGAAGACCCTGGGCTGGGAGGCCAACACCGAGATGCTGTACCCCGCCGATGGCGGCCTGGAGGGCCGCACCGATATGGCCCTGAAGCTGGTCGGCGGCGGCCACCTGATCTGCAACTTCAAGACCACCTACCGCAGCAAGAAGCCCGCCAAGAACCTGAAGATGCCCGGCGTGTACTACGTGGATCACCGCCTGGAGCGCATTAAGGAGGCCGATAAGGAGACCTACGTCGAGCAGCACGAGGTGGCCGTGGCCCGCTACTGCGATCTGCCGTCCAAGCTGGGACACAAGCTGAACGGCATGGATGAGCTGTACAAGACTAGTTAAAATGAATCGTTTTTAAAATAACAAATCAATTGTTTTATAATATTCGTACGATTCTTTGATTATGTAATAAAATGTGATCATTAGGAAGATTACGAAAAATATAAAAAATATGAGTTCTGTGTGTATAACAAATGCTGTAAACGCCACAATTGTGTTTGTTGCAAATAAACCCATGATTATTTGATTAAAATTGTTGTTTTCTTTGTTCATAGACAATAGTGTGTTTTGCCTAAACGTGTACTGCATAAACTCCATGCGAGTGTATAGCGAGCTAGTGGCTAACGCTTGCCCCACCAAAGTAGATTCGTCAAAATCCTCAATTTCATCACCCTCCTCCAAGTTTAACATTTGGCCGTCGGAATTAACTTCTAAAGATGCCACATAATCTAATAAATGAAATAGAGATTCAAACGTGGCGTCATCGTCCGTTTCGACCATTTCCGAAAAGAACTCGGGCATAAACTCTATGATTTCTCTGGACGTGGTGTTGTCGAAACTCTCAAAGTACGCAGTCAGGAACGTGCGCGACATGTCGTCGGGAAACTCGCGCGGAAACATGTTGTTGTAACCGAACGGGTCCCATAGCGCCAAAACCAAATCTGCCAGCGTCAATAGAATGAGCACGATGCCGACAATGGAGCTGGCTTGGATAGCGATTCGAGTTAACGGCCGGCCGCTAGTTGGCCACGTAATAAGTGTGCGTTGAATTTATTCGCAAAAACATTGCATATTTTCGGCAAAGTAAAATTTTGTTGCATACCTTATCAAAAAATAAGTGCTGCATACTTTTTAGAGAAACCAAATAATTTTTTATTGCATACCCGTTTTTAATAAAATACATTGCATACCCTCTTTTAATAAAAAATATTGCATACTTTGACGAAACAAATTTTCGTTGCATACCCAATAAAAGATTATTATATTGCATACCCGTTTTTAATAAAATACATTGCATACCCTCTTTTAATAAARAATATTGCATACGTTGACGAAACAAATTTTCGTTGCATACCCAATAAAAGATTATTATATTGCATACCTTTTCTTGCCATACCATTTAGCCGATCAATTGTGCTCGGCAACAGTATATTTGTGGTGTGCCAACCAACAACTCTAGTGCTGCATCCAACGCGTTGGGAGCTCTCCGGATCAATTCGGCTTCAGGTACCGTCGACGATGTAGGTCACGGTCTCGAAGCCGCGGTGCGGGTGCCAGGGCGTGCCCTTGGGCTCCCCGGGCGCGTACTCCACCTCACCCATCTGGTCCATCATGATGAACGGGTCGAGGTGGCGGTAGTTGATCCCGGCGAACGCGCGGCGCACCGGGAAGCCCTCGCCCTCGAAACCGCTGGGCGCGGTGGTCACGGTGAGCACGGGACGTGCGACGGCGTCGGCGGGTGCGGATACGCGGGGCAGCGTCAGCGGGTTCTCGACGGTCACGGCGGGCATGTCGACAAGCCGAATTGATCCACTAGAAGGCCTAATTCGCGGCCGCGGATCTGATCCCGGGCGGGTACCGAATTCTAGTATGTATGTAAGTTAATAAAACCCTTTTTTGGAGAATGTAGATTTAAAAAAACATATTTTTTTTTTATTTTTTACTGCACTGGATATCATTGAACTTATCTGATCAGTTTTAAATTTACTTCGATCCAAGGGTATTTGAAGTACCAGGTTCTTTCGATTACCTCTCACTCAAAATGACATTCCACTCAAAGTCAGCGCTGTTTGCCTCCTTCTCTGTCCACAGAAATATCGCCGTCTCTTTCGCCGCTGCGTCCGCTATCTCTTTCGCCACCGTTTGTAGCGTTACCTAGCGTCAATGTCCGCCTTCAGTTGCACTTTGTCAGCGGTTTCGTGACGAAGCTCCAAGCGGTTTACGCCATCAATTAAACACAAAGTGCTGTGCCAAAACTCCTCTCGCTTCTTATTTTTGTTTGTTTTTTGAGTGATTGGGGTGGTGATTGGTTTTGGGTGGGTAAGCAGGGGAAAGTGTGAAAAATCCCGGCAATGGGCCAAGAGGATCAGGAGCTATTAATTCGCGGAGGCAGCAAACACCCATCTGCCGAGCATCTGAACAATGTGAGTAGTACATGTGCATACATCTTAAGTTCACTTGATCTATAGGAACTGCGATTGCAACATCAAATTGTCTGCGGCGTGAGAACTGCGACCCACAAAAATCCCAAACCGCAATCGCACAAACAAATAGTGACACGAAACAGATTATTCTGGTAGCTGTGCTCGCTATATAAGACAATTTTTAAGATCATATCATGATCAAGACATCTAAAGGCATTCATTTTCGACTACATTCTTTTTTACAAAAAATATAACAACCAGATATTTTAAGCTGATCCTAGATGCACAAAAAATAAATAAAAGTATAAACCTACTTCGTAGGATACTTCGTTTTGTTCGGGGTTAGATGAGCATAACGCTTGTAGTTGATATTTGAGATCCCCTATCATTGCAGGGTGACAGCGGACGCTTCGCAGAGCTGCATTAACCAGGGCTTCGGGCAGGCCAAAAACTACGGCACGCTCCTGCCACCCAGTCCGCCGGAGGACTCCGGTTCAGGGAGCGGCCAACTAGCCGAGAACCTCACCTATGCCTGGCACAATATGGACATCTTTGGGGCGGTCAATCAGCCGGGCTCCGGATGGCGGCAGCTGGTCAACCGGACACGCGGACTATTCTGCAACGAGCGACACATACCGGCGCCCAGGAAACATTTGCTCAAGAACGGTGAGTTTCTATTCGCAGTCGGCTGATCTGTGTGAAATCTTAATAAAGGGTCCAATTACCAATTTGAAACTCAGTTTGCGGCGTGGCCTATCCGGGCGAACTTTTGGCCGTGATGGGCAGTTCCGGTGCCGGAAAGACGACCCTGCTGAATGCCCTTGCCTTTCGATCGCCGCAGGGCATCCAAGTATCGCCATCCGGGATGCGACTGCTCAATGGCCAACCTGTGGACGCCAAGGAGATGCAGGCCAGGTGCGCCTATGTCCAGCAGGATGACCTCTTTATCGGCTCCCTAACGGCCAGGGAACACCTGATTTTCCAGGCCATGGTGCGGATGCCACGACATCTGACCTATCGGCAGCGAGTGGCCCGCGTGGATCAGGTGATCCAGGAGCTTTCGCTCAGCAAATGTCAGCACACGATCATCGGTGTGCCCGGCAGGGTGAAAGGTCTGTCCGGCGGAGAAAGGAAGCGTCTGGCATTCGCCTCCGAGGCACTAACCGATCCGCCGCTTCTGATCTGCGATGAGCCCACCTCCGGACTGGACTCATTTACCGCCCACAGCGTCGTCCAGGTGCTGAAGAAGCTGTCGCAGAAGGGCAAGACCGTCATCCTGACCATTCATCAGCCGTCTTCCGAGCTGTTTGAGCTCTTTGACAAGATCCTTCTGATGGCCGAGGGCAGGGTAGCTTTCTTGGGCACTCCCAGCGAAGCCGTCGACTTCTTTTCCTAGTGAGTTCGATGTGTTTATTAAGGGTATCTAGCATTACATTACATCTCAACTCCTATCCAGCGTGGGTGCCCAGTGTCCTACCAACTACAATCCGGCGGACTTTTACGTACAGGTGTTGGCCGTTGTGCCCGGACGGGAGATCGAGTCCCGTGATCGGATCGCCAAGATATGGCGACAATTTTGCTATTAGCAAAGTAGCCCGGGATATGGAGCAGTTGTTGGCCACCAAAAATTTGGAGAAGCCACTGGAGCAGCCGGAGAATGGGTACACCTACAAGGCCACCTGGTTCATGCAGTTCCGGGCGGTCCTGTGGCGATCCTGGCTGTCGGTGCTCAAGGAACCACTCCTCGTAAAAGTGCGACTTATTCAGACAACGGTGAGTGGTTCCAGTGGAAACAAATGATATAACGCTTACAATTCTTGGAAACAAATTCGCTAGATTTTAGTTAGAATTGCCTGATTCCACACCCTTCTTAGTTTTTTTCAATGAGATGTATAGTTTATAGTTTTGCAGAAAATAAATAAATTTCATTTAACTCGCGAACATGTTGAAGATATGAATATTAATGAGATGCGAGTAACATTTTAATTTGCAGATGGTTGCCATCTTGATTGGCCTCATCTTTTTGGGCCAACAACTCACGCAAGTGGGCGTGATGAATATCAACGGAGCCATCTTCCTCTTCCTGACCAACATGACCTTTCAAAACGTCTTTGCCACGATAAATGTAAGTCTTGTTTAGAATACATTTGCATATTAATAATTTACTAACTTTCTAATGAATCGATTCGATTTAGGTGTTCACCTCAGAGCTGCCAGTTTTTATGAGGGAGGCCCGAAGTCGACTTTATCGCTGTGACACATACTTTCTGGGCAAAACGATTGCCGAATTACCGCTTTTTCTCACAGTGCCACTGGTCTTCACGGCGATTGCCTATCCGATGATCGGACTGCGGGCCGGAGTGCTGCACTTCTTCAACTGCCTGGCGCTGGTCACTCTGGTGGCCAATGTGTCAACGTCCTTCGGATATCTAATATCCTGCGCCAGCTCCTCGACCTCGATGGCGCTGTCTGTGGGTCCGCCGGTTATCATACCATTCCTGCTCTTTGGCGGCTTCTTCTTGAACTCGGGCTCGGTGCCAGTATACCTCAAATGGTTGTCGTACCTCTCATGGTTCCGTTACGCCAACGAGGGTCTGCTGATTAACCAATGGGCGGACGTGGAGCCGGGCGAAATTAGCTGCACATCGTCGAACACCACGTGCCCCAGTTCGGGCAAGGTCATCCTGGAGACGCTTAACTTCTCCGCCGCCGATCTGCCGCTGGACTACGTGGGTCTGGCCATTCTCATCGTGAGCTTCCGGGTGCTCGCATATCTGGCTCTAAGACTTCGGGCCCGACGCAAGGAGTAGCCGACATATATCCGAAATAACTGCTTGTTTTTTTTTTTACCATTATTACCATCGTGTTTACTGTTTATTGCCCCCTCAAAAAGCTAATGTAATTATATTTGTGCCAATAAAAACAAGATATGACCTATAGAATACAAGTATTTCCCCTTCGAACATCCCCACAAGTAGACTTTGGATTTGTCTTCTAACCAAAAGACTTACACACCTGCATACCTTACATCAAAAACTCGTTTATCGCTACATAAAACACCGGGATATATTTTTTATATACATACTTTTCAAATCGCGCGCCCTCTTCATAATTCACCTCCACCACACCACGTTTCGTAGTTGCTCTTTCGCTGTCTCCCACCCGCTCTCCGCAACACATTCACCTTTTGTTCGACGACCTTGGAGCGACTGTCGTTAGTTCCGCGCGATTCGGTTCGCTCAAATGGTTCCGAGTGGTTCATTTCGTCTCAATAGAAATTAGTAATAAATATTTGTATGTACAATTTATTTGCTCCAATATATTTGTATATATTTCCCTCACAGCTATATTTATTCTAATTTAATATTATGACTTTTTAAGGTAATTTTTTGTGACCTGTTCGGAGTGATTAGCGTTACAATTTGAACTGAAAGTGACATCCAGTGTTTGTTCCTTGTGTAGATGCATCTCAAAAAAATGGTGGGCATAATAGTGTTGTTTATATATATCAAAAATAACAACTATAATAATAAGAATACATTTAATTTAGAAAATGCTTGGATTTCACTGGAACTAGAATTAATTCGGCTGCTGCTCTAAACGACGCATTTCGTACTCCAAAGTACGAATTTTTTCCCTCAAGCTCTTATTTTCATTAAACAATGAACAGGACCTAACGCACAGTCACGTTATTGTTTACATAAATGATTTTTTTTACTATTCAAACTTACTCTGTTTGTGTACTCCCACTGGTATAGCCTTCTTTTATCTTTTCTGGTTCAGGCTCTATCACTTTACTAGGTACGGCATCTGCGTTGAGTCGCCTCCTTTTAAATGTCTGACCTTTTGCAGGTGCAGCCTTCCACTGCGAATCTTTAAAGTGGGTATCACAAATTTGGGAGTTTTCACCAAGGCTGCACCCAAGGCTCTGCTCCCACAATTTTCTCTTAATAGCACACTTCGGCACGTGAATTAATTTTACTCCAGTCACAGCTTTGCAGCAAAATTTGCAATATTTCATTTTTTTTTATTCCACGTAAGGGTTAATGTTTTCAAAAAAAAATTCGTCCGCACACAACCTTTCCTCTCAACAAGCAAACGTGCACTGAATTTAAGTGTATACTTCGGTAAGCTTCGGCTATCGACGGGACCACCTTATGTTATTTCATCATGGGCCAGACCCACGTAGTCCAGCGGCAGATCGGCGGCGGAGAAGTTAAGCGTCTCCAGGATGACCTTGCCCGAACTGGGGCACGTGGTGTTCGACGATGTGCAGCTAATTTCGCCCGGCTCCACGTCCGCCCATTGGTTAATCAGCAGACCCTCGTTGGCGTAACGGAACCATGAGAGGTACGACAACCATTTGAGGTATACTGGCACCGAGCCCGAGTTCAAGAAGAAGGCGTTTTTCCATAGGCTCCGCCCCCCTGACGAGCATCACAAAAATCGACGCTCAAGTCAGAGGTGGCGAAACCCGACAGGACTATAAAGATACCAGGCGTTTCCCCCTGGAAGCTCCCTCGTGCGCTCTCCTGTTCCGACCCTGCCGCTTACCGGATACCTGTCCGCCTTTCTCCCTTCGGGAAGCGTGGCGCTTTCTCAATGCTCACGCTGTAGGTATCTCAGTTCGGTGTAGGTCGTTCGCTCCAAGCTGGGCTGTGTGCACGAACCCCCCGTTCAGCCCGACCGCTGCGCCTTATCCGGTAACTATCGTCTTGAGTCCAACCCGGTAAGACACGACTTATCGCCACTGGCAGCAGCCACTGGTAACAGGATTAGCAGAGCGAGGTATGTAGGCGGTGCTACAGAGTTCTTGAAGTGGTGGCCTAACTACGGCTACACTAGAAGGACAGTATTTGGTATCTGCGCTCTGCTGAAGCCAGTTACCTTCGGAAAAAGAGTTGGTAGCTCTTGATCCGGCAAACAAACCACCGCTGGTAGCGGTGGTTTTTTTGTTTGCAAGCAGCAGATTACGCGCAGAAAAAAAGGATCTCAAGAAGATCCTTTGATCTTTTCTACGGGGTCTGACGCTCAGTGGAACGAAAACTCACGTTAAGGGATTTTGGTCATGAGATTATCAAAAAGGATCTTCACCTAGATCCTTTTAAATTAAAAATGAAGTTTTAAATCAATCTAAAGTATATATGAGTAAACTTGGTCTGACAGTTACCAATGCTTAATCAGTGAGGCACCTATCTCAGCGATCTGTCTATTTCGTTCATCCATAGTTGCCTGACTCCCCGTCGTGTAGATAACTACGATACGGGAGGGCTTACCATCTGGCCCCAGTGCTGCAATGATACCGCGAGACCCACGCTCACCGGCTCCAGATTTATCAGCAATAAACCAGCCAGCCGGAAGGGCCGAGCGCAGAAGTGGTCCTGCAACTTTATCCGCCTCCATCCAGTCTATTAATTGTTGCCGGGAAGCTAGAGTAAGTAGTTCGCCAGTTAATAGTTTGCGCAACGTTGTTGCCATTGCTACAGGCATCGTGGTGTCACGCTCGTCGTTTGGTATGGCTTCATTCAGCTCCGGTTCCCAACGATCAAGGCGAGTTACATGATCCCCCATGTTGTGCAAAAAAGCGGTTAGCTCCTTCGGTCCTCCGATCGTTGTCAGAAGTAAGTTGGCCGCAGTGTTATCACTCATGGTTATGGCAGCACTGCATAATTCTCTTACTGTCATGCCATCCGTAAGATGCTTTTCTGTGACTGGTGAGTACTCAACCAAGTCATTCTGAGAATAGTGTATGCGGCGACCGAGTTGCTCTTGCCCGGCGTCAATACGGGATAATACCGCGCCACATAGCAGAACTTTAAAAGTGCTCATCATTGGAAAACGTTCTTCGGGGCGAAAACTCTCAAGGATCTTACCGCTGTTGAGATCCAGTTCGATGTAACCCACTCGTGCACCCAACTGATCTTCAGCATCTTTTACTTTCACCAGCGTTTCTGGGTGAGCAAAAACAGGAAGGCAAAATGCCGCAAAAAAGGGAATAAGGGCGACACGGAAATGTTGAATACTCATACTCTTCCTTTTTCAATATTATTGAAGCATTTATCAGGGTTATTGTCTCATGAGCGGATACATATTTGAATGTATTTAGAAAAATAAACAAATAGGGGTTCCGCGCACATTTCCCCGAAAAGTGCCACCTGACGTCTAAGAAACCATTATTATCATGACATTAACCTATAAAAATAGGCGTATCACGAGGCCCTTTCGTCTCGCGCGTTTCGGTGATGACGGTGAAAACCTCTGACACATGCAGCTCCCGGAGACGGTCACAGCTTGTCTGTAAGCGGATGCCGGGAGCAGACAAGCCCGTCAGGGCGCGTCAGCGGGTGTTGGCGGGTGTCGGGGCTGGCTTAACTATGCGGCATCAGAGCAGATTGTACTGAGAGTGCACCATATGCGGTGTGAAATACCGCACCGAATCGCGCGGAACTAACGACAGTCGCTCCAAGGTCGTCGAACAAAAGGTGAATGTGTTGCGGAGAGCGGGTGGGAGACAGCGAAAGAGCAACTACGAAACGTGGTGTGGTGGAGGTGAATTATGAAGAGGGCGCGCGATTTGAAAAGTATGTATATAAAAAATATATCCCGGTGTTTTATGTAGCGATAAACGAGTTTTTGATGTAAGGTATGCAGGTGTGTAAGTCTTTTGGTTAGAAGACAAATCCAAAGTCTACTTGTGGGGATGTTCGAAGGGGAAATACTTGTATTCTATAGGTCATATCTTGTTTTTATTGGCACAAATATAATTACATTAGCTTTTTGAGGGGGCAATAAACAGTAAACACGATGGTAATAATGGTAAAAAAAAAAACAAGCAGTTATTTCGGATATATGTCGGCTACTCCTTGCGTCGGGCCCGAAGTCTTAGAGCCAGATATGCGAGCACCCGGAAGCTCACGATGAGAATGGCCAGACCATGATGAAATAACATAAGGTGGTCCCGTCGGCAAGAGACATCCACTTAACGTATGCTTGCAATAAGTGCGAGTGAAAGGAATAGTATTCTGAGTGTCGTATTGAGTCTGAGTGAGACAGCGATATGATTGTTGATTAACCCTTAGCATGTCCGTGGGGTTTGAATTAACTCATAATATTAATTAGACGAAATTATTTTTAAAGTTTTATTTTTAATAATTTGCGAGTACGCA

**pCaSpeR-gypsy-HSPmini-nlsGFP-SV40-gypsy-attB2**

AAGCTTGGGCTGCAGGTCGACCTCGAGGCCTCGAGTTAACGTTACGTTAACGTTAACGTTCGAGGTCGACTCTAGTTGGCCACGTAATAAGTGTGCGTTGAATTTATTCGCAAAAACATTGCATATTTTCGGCAAAGTAAAATTTTGTTGCATACCTTATCAAAAAATAAGTGCTGCATACTTTTTAGAGAAACCAAATAATTTTTTATTGCATACCCGTTTTTAATAAAATACATTGCATACCCTCTTTTAATAAAAAATATTGCATACTTTGACGAAACAAATTTTCGTTGCATACCCAATAAAAGATTATTATATTGCATACCCGTTTTTAATAAAATACATTGCATACCCTCTTTTAATAAARAATATTGCATACGTTGACGAAACAAATTTTCGTTGCATACCCAATAAAAGATTATTATATTGCATACCTTTTCTTGCCATACCATTTAGCCGATCAATTGTGCTCGGCAACAGTATATTTGTGGTGTGCCAACCAACAACTCTAGAGGATCCAGATCCACTAGCGAGCGGAGACTCTAGCGCTAGCGACGTCGAGCGCCGGAGTATAAATAGAGGCGCTTCGTCTACGGAGCGACAATTCAATTCAAACAAGCAAAGTGAACACGTCGCTAAGCGAAAGCTAAGCAAATAAACAAGCGCAGCTGAACAAGCTAAACAATCTGCAGTAAAGTGCAAGTTAAAGTGAATCAATTAAAAGTAACCAGCAACCAAGTAAATCAACTGCAACTACTGAAATCTGCCAAGAAGTAATTATTGAATACAAGAAGAGAACTCTGAATAATGCCCAAGAAGAAGCGCAAGGTGGTGTCCAAGGGCGAGGAGCTGTTCACCGGCGTGGTGCCCATCCTGGTGGAGCTGGATGGCGACGTGAACGGCCACAAGTTCAGCGTGCGCGGCGAGGGCGAGGGCGACGCCACCAACGGCAAGCTGACCCTGAAGTTCATCTGCACCACCGGCAAGCTGCCCGTGCCCTGGCCCACCCTGGTGACCACCCTGACCTACGGCGTGCAGTGCTTCAGCCGCTACCCCGATCACATGAAGCAGCACGATTTCTTCAAGAGCGCCATGCCCGAGGGCTACGTGCAGGAGCGCACCATCAGCTTCAAGGATGACGGCACCTACAAGACCCGCGCCGAGGTGAAGTTCGAGGGCGATACCCTGGTGAACCGCATCGAGCTGAAGGGCATCGATTTCAAGGAGGATGGCAACATCCTGGGCCACAAGCTGGAGTACAACTTCAACAGCCACAACGTGTACATCACCGCCGATAAGCAGAAGAACGGCATCAAGGCCAACTTCAAGATCCGCCACAATGTGGAGGATGGCTCCGTGCAGCTGGCCGATCACTACCAGCAGAACACCCCCATCGGCGACGGCCCAGTGCTGCTGCCCGATAACCACTACCTGAGCACCCAGAGCGTGCTGTCCAAGGACCCCAACGAGAAGCGCGATCACATGGTGCTGCTGGAGTTCGTGACCGCCGCCGGCATCACCCTGGGCATGGATGAGCTGTACAAGACTAGTTAACGGGATCTTTGTGAAGGAACCTTACTTCTGTGGTGTGACATAATTGGACAAACTACCTACAGAGATTTAAAGCTCTAAGGTAAATATAAAATTTTTAAGTGTATAATGTGTTAAACTACTGATTCTAATTGTTTGTGTATTTTAGATTCCAACCTATGGAACTGATGAATGGGAGCAGTGGTGGAATGCCTTTAATGAGGAAAACCTGTTTTGCTCAGAAGAAATGCCATCTAGTGATGATGAGGCTACTGCTGACTCTCAACATTCTACTCCTCCAAAAAAGAAGAGAAAGGTAGAAGACCCCAAGGACTTTCCTTCAGAATTGCTAAGTTTTTTGAGTCATGCTGTGTTTAGTAATAGAACTCTTGCTTGCTTTGCTATTTACACCACAAAGGAAAAAGCTGCACTGCTATACAAGAAAATTATGGAAAAATATTTGATGTATAGTGCCTTGACTAGAGATCATAATCAGCCATACCACATTTGTAGAGGTTTTACTTGCTTTAAAAAACCTCCCACACCTCCCCCTGAACCTGAAACATAAAATGAATGGAATTGTTGTTGTTAACTTGTTTATTGCAGCTTATAATGGTTACAAATAAAGCAATAGCATCACAAATTTCACAAATAAAGCATTTTTTTCACTGCATTCTAGTTGTGGTTTGTCCAAACTCATCAATGTATCTTATCATGTCTGGTTCCAGAGCTAGTTGGCCACGTAATAAGTGTGCGTTGAATTTATTCGCAAAAACATTGCATATTTTCGGCAAAGTAAAATTTTGTTGCATACCTTATCAAAAAATAAGTGCTGCATACTTTTTAGAGAAACCAAATAATTTTTTATTGCATACCCGTTTTTAATAAAATACATTGCATACCCTCTTTTAATAAAAAATATTGCATACTTTGACGAAACAAATTTTCGTTGCATACCCAATAAAAGATTATTATATTGCATACCCGTTTTTAATAAAATACATTGCATACCCTCTTTTAATAAARAATATTGCATACGTTGACGAAACAAATTTTCGTTGCATACCCAATAAAAGATTATTATATTGCATACCTTTTCTTGCCATACCATTTAGCCGATCAATTGTGCTCGGCAACAGTATATTTGTGGTGTGCCAACCAACAACTCTAGTGCTGCATCCAACGCGTTGGGAGCTCTCCGGATCAATTCGGCTTCAGGTACCGTCGACGATGTAGGTCACGGTCTCGAAGCCGCGGTGCGGGTGCCAGGGCGTGCCCTTGGGCTCCCCGGGCGCGTACTCCACCTCACCCATCTGGTCCATCATGATGAACGGGTCGAGGTGGCGGTAGTTGATCCCGGCGAACGCGCGGCGCACCGGGAAGCCCTCGCCCTCGAAACCGCTGGGCGCGGTGGTCACGGTGAGCACGGGACGTGCGACGGCGTCGGCGGGTGCGGATACGCGGGGCAGCGTCAGCGGGTTCTCGACGGTCACGGCGGGCATGTCGACAAGCCGAATTGATCCACTAGAAGGCCTAATTCGCGGCCGCGGATCTGATCCCGGGCGGGTACCGAATTCTAGTATGTATGTAAGTTAATAAAACCCTTTTTTGGAGAATGTAGATTTAAAAAAACATATTTTTTTTTTATTTTTTACTGCACTGGATATCATTGAACTTATCTGATCAGTTTTAAATTTACTTCGATCCAAGGGTATTTGAAGTACCAGGTTCTTTCGATTACCTCTCACTCAAAATGACATTCCACTCAAAGTCAGCGCTGTTTGCCTCCTTCTCTGTCCACAGAAATATCGCCGTCTCTTTCGCCGCTGCGTCCGCTATCTCTTTCGCCACCGTTTGTAGCGTTACCTAGCGTCAATGTCCGCCTTCAGTTGCACTTTGTCAGCGGTTTCGTGACGAAGCTCCAAGCGGTTTACGCCATCAATTAAACACAAAGTGCTGTGCCAAAACTCCTCTCGCTTCTTATTTTTGTTTGTTTTTTGAGTGATTGGGGTGGTGATTGGTTTTGGGTGGGTAAGCAGGGGAAAGTGTGAAAAATCCCGGCAATGGGCCAAGAGGATCAGGAGCTATTAATTCGCGGAGGCAGCAAACACCCATCTGCCGAGCATCTGAACAATGTGAGTAGTACATGTGCATACATCTTAAGTTCACTTGATCTATAGGAACTGCGATTGCAACATCAAATTGTCTGCGGCGTGAGAACTGCGACCCACAAAAATCCCAAACCGCAATCGCACAAACAAATAGTGACACGAAACAGATTATTCTGGTAGCTGTGCTCGCTATATAAGACAATTTTTAAGATCATATCATGATCAAGACATCTAAAGGCATTCATTTTCGACTACATTCTTTTTTACAAAAAATATAACAACCAGATATTTTAAGCTGATCCTAGATGCACAAAAAATAAATAAAAGTATAAACCTACTTCGTAGGATACTTCGTTTTGTTCGGGGTTAGATGAGCATAACGCTTGTAGTTGATATTTGAGATCCCCTATCATTGCAGGGTGACAGCGGACGCTTCGCAGAGCTGCATTAACCAGGGCTTCGGGCAGGCCAAAAACTACGGCACGCTCCTGCCACCCAGTCCGCCGGAGGACTCCGGTTCAGGGAGCGGCCAACTAGCCGAGAACCTCACCTATGCCTGGCACAATATGGACATCTTTGGGGCGGTCAATCAGCCGGGCTCCGGATGGCGGCAGCTGGTCAACCGGACACGCGGACTATTCTGCAACGAGCGACACATACCGGCGCCCAGGAAACATTTGCTCAAGAACGGTGAGTTTCTATTCGCAGTCGGCTGATCTGTGTGAAATCTTAATAAAGGGTCCAATTACCAATTTGAAACTCAGTTTGCGGCGTGGCCTATCCGGGCGAACTTTTGGCCGTGATGGGCAGTTCCGGTGCCGGAAAGACGACCCTGCTGAATGCCCTTGCCTTTCGATCGCCGCAGGGCATCCAAGTATCGCCATCCGGGATGCGACTGCTCAATGGCCAACCTGTGGACGCCAAGGAGATGCAGGCCAGGTGCGCCTATGTCCAGCAGGATGACCTCTTTATCGGCTCCCTAACGGCCAGGGAACACCTGATTTTCCAGGCCATGGTGCGGATGCCACGACATCTGACCTATCGGCAGCGAGTGGCCCGCGTGGATCAGGTGATCCAGGAGCTTTCGCTCAGCAAATGTCAGCACACGATCATCGGTGTGCCCGGCAGGGTGAAAGGTCTGTCCGGCGGAGAAAGGAAGCGTCTGGCATTCGCCTCCGAGGCACTAACCGATCCGCCGCTTCTGATCTGCGATGAGCCCACCTCCGGACTGGACTCATTTACCGCCCACAGCGTCGTCCAGGTGCTGAAGAAGCTGTCGCAGAAGGGCAAGACCGTCATCCTGACCATTCATCAGCCGTCTTCCGAGCTGTTTGAGCTCTTTGACAAGATCCTTCTGATGGCCGAGGGCAGGGTAGCTTTCTTGGGCACTCCCAGCGAAGCCGTCGACTTCTTTTCCTAGTGAGTTCGATGTGTTTATTAAGGGTATCTAGCATTACATTACATCTCAACTCCTATCCAGCGTGGGTGCCCAGTGTCCTACCAACTACAATCCGGCGGACTTTTACGTACAGGTGTTGGCCGTTGTGCCCGGACGGGAGATCGAGTCCCGTGATCGGATCGCCAAGATATGGCGACAATTTTGCTATTAGCAAAGTAGCCCGGGATATGGAGCAGTTGTTGGCCACCAAAAATTTGGAGAAGCCACTGGAGCAGCCGGAGAATGGGTACACCTACAAGGCCACCTGGTTCATGCAGTTCCGGGCGGTCCTGTGGCGATCCTGGCTGTCGGTGCTCAAGGAACCACTCCTCGTAAAAGTGCGACTTATTCAGACAACGGTGAGTGGTTCCAGTGGAAACAAATGATATAACGCTTACAATTCTTGGAAACAAATTCGCTAGATTTTAGTTAGAATTGCCTGATTCCACACCCTTCTTAGTTTTTTTCAATGAGATGTATAGTTTATAGTTTTGCAGAAAATAAATAAATTTCATTTAACTCGCGAACATGTTGAAGATATGAATATTAATGAGATGCGAGTAACATTTTAATTTGCAGATGGTTGCCATCTTGATTGGCCTCATCTTTTTGGGCCAACAACTCACGCAAGTGGGCGTGATGAATATCAACGGAGCCATCTTCCTCTTCCTGACCAACATGACCTTTCAAAACGTCTTTGCCACGATAAATGTAAGTCTTGTTTAGAATACATTTGCATATTAATAATTTACTAACTTTCTAATGAATCGATTCGATTTAGGTGTTCACCTCAGAGCTGCCAGTTTTTATGAGGGAGGCCCGAAGTCGACTTTATCGCTGTGACACATACTTTCTGGGCAAAACGATTGCCGAATTACCGCTTTTTCTCACAGTGCCACTGGTCTTCACGGCGATTGCCTATCCGATGATCGGACTGCGGGCCGGAGTGCTGCACTTCTTCAACTGCCTGGCGCTGGTCACTCTGGTGGCCAATGTGTCAACGTCCTTCGGATATCTAATATCCTGCGCCAGCTCCTCGACCTCGATGGCGCTGTCTGTGGGTCCGCCGGTTATCATACCATTCCTGCTCTTTGGCGGCTTCTTCTTGAACTCGGGCTCGGTGCCAGTATACCTCAAATGGTTGTCGTACCTCTCATGGTTCCGTTACGCCAACGAGGGTCTGCTGATTAACCAATGGGCGGACGTGGAGCCGGGCGAAATTAGCTGCACATCGTCGAACACCACGTGCCCCAGTTCGGGCAAGGTCATCCTGGAGACGCTTAACTTCTCCGCCGCCGATCTGCCGCTGGACTACGTGGGTCTGGCCATTCTCATCGTGAGCTTCCGGGTGCTCGCATATCTGGCTCTAAGACTTCGGGCCCGACGCAAGGAGTAGCCGACATATATCCGAAATAACTGCTTGTTTTTTTTTTTACCATTATTACCATCGTGTTTACTGTTTATTGCCCCCTCAAAAAGCTAATGTAATTATATTTGTGCCAATAAAAACAAGATATGACCTATAGAATACAAGTATTTCCCCTTCGAACATCCCCACAAGTAGACTTTGGATTTGTCTTCTAACCAAAAGACTTACACACCTGCATACCTTACATCAAAAACTCGTTTATCGCTACATAAAACACCGGGATATATTTTTTATATACATACTTTTCAAATCGCGCGCCCTCTTCATAATTCACCTCCACCACACCACGTTTCGTAGTTGCTCTTTCGCTGTCTCCCACCCGCTCTCCGCAACACATTCACCTTTTGTTCGACGACCTTGGAGCGACTGTCGTTAGTTCCGCGCGATTCGGTTCGCTCAAATGGTTCCGAGTGGTTCATTTCGTCTCAATAGAAATTAGTAATAAATATTTGTATGTACAATTTATTTGCTCCAATATATTTGTATATATTTCCCTCACAGCTATATTTATTCTAATTTAATATTATGACTTTTTAAGGTAATTTTTTGTGACCTGTTCGGAGTGATTAGCGTTACAATTTGAACTGAAAGTGACATCCAGTGTTTGTTCCTTGTGTAGATGCATCTCAAAAAAATGGTGGGCATAATAGTGTTGTTTATATATATCAAAAATAACAACTATAATAATAAGAATACATTTAATTTAGAAAATGCTTGGATTTCACTGGAACTAGAATTAATTCGGCTGCTGCTCTAAACGACGCATTTCGTACTCCAAAGTACGAATTTTTTCCCTCAAGCTCTTATTTTCATTAAACAATGAACAGGACCTAACGCACAGTCACGTTATTGTTTACATAAATGATTTTTTTTACTATTCAAACTTACTCTGTTTGTGTACTCCCACTGGTATAGCCTTCTTTTATCTTTTCTGGTTCAGGCTCTATCACTTTACTAGGTACGGCATCTGCGTTGAGTCGCCTCCTTTTAAATGTCTGACCTTTTGCAGGTGCAGCCTTCCACTGCGAATCTTTAAAGTGGGTATCACAAATTTGGGAGTTTTCACCAAGGCTGCACCCAAGGCTCTGCTCCCACAATTTTCTCTTAATAGCACACTTCGGCACGTGAATTAATTTTACTCCAGTCACAGCTTTGCAGCAAAATTTGCAATATTTCATTTTTTTTTATTCCACGTAAGGGTTAATGTTTTCAAAAAAAAATTCGTCCGCACACAACCTTTCCTCTCAACAAGCAAACGTGCACTGAATTTAAGTGTATACTTCGGTAAGCTTCGGCTATCGACGGGACCACCTTATGTTATTTCATCATGGGCCAGACCCACGTAGTCCAGCGGCAGATCGGCGGCGGAGAAGTTAAGCGTCTCCAGGATGACCTTGCCCGAACTGGGGCACGTGGTGTTCGACGATGTGCAGCTAATTTCGCCCGGCTCCACGTCCGCCCATTGGTTAATCAGCAGACCCTCGTTGGCGTAACGGAACCATGAGAGGTACGACAACCATTTGAGGTATACTGGCACCGAGCCCGAGTTCAAGAAGAAGGCGTTTTTCCATAGGCTCCGCCCCCCTGACGAGCATCACAAAAATCGACGCTCAAGTCAGAGGTGGCGAAACCCGACAGGACTATAAAGATACCAGGCGTTTCCCCCTGGAAGCTCCCTCGTGCGCTCTCCTGTTCCGACCCTGCCGCTTACCGGATACCTGTCCGCCTTTCTCCCTTCGGGAAGCGTGGCGCTTTCTCAATGCTCACGCTGTAGGTATCTCAGTTCGGTGTAGGTCGTTCGCTCCAAGCTGGGCTGTGTGCACGAACCCCCCGTTCAGCCCGACCGCTGCGCCTTATCCGGTAACTATCGTCTTGAGTCCAACCCGGTAAGACACGACTTATCGCCACTGGCAGCAGCCACTGGTAACAGGATTAGCAGAGCGAGGTATGTAGGCGGTGCTACAGAGTTCTTGAAGTGGTGGCCTAACTACGGCTACACTAGAAGGACAGTATTTGGTATCTGCGCTCTGCTGAAGCCAGTTACCTTCGGAAAAAGAGTTGGTAGCTCTTGATCCGGCAAACAAACCACCGCTGGTAGCGGTGGTTTTTTTGTTTGCAAGCAGCAGATTACGCGCAGAAAAAAAGGATCTCAAGAAGATCCTTTGATCTTTTCTACGGGGTCTGACGCTCAGTGGAACGAAAACTCACGTTAAGGGATTTTGGTCATGAGATTATCAAAAAGGATCTTCACCTAGATCCTTTTAAATTAAAAATGAAGTTTTAAATCAATCTAAAGTATATATGAGTAAACTTGGTCTGACAGTTACCAATGCTTAATCAGTGAGGCACCTATCTCAGCGATCTGTCTATTTCGTTCATCCATAGTTGCCTGACTCCCCGTCGTGTAGATAACTACGATACGGGAGGGCTTACCATCTGGCCCCAGTGCTGCAATGATACCGCGAGACCCACGCTCACCGGCTCCAGATTTATCAGCAATAAACCAGCCAGCCGGAAGGGCCGAGCGCAGAAGTGGTCCTGCAACTTTATCCGCCTCCATCCAGTCTATTAATTGTTGCCGGGAAGCTAGAGTAAGTAGTTCGCCAGTTAATAGTTTGCGCAACGTTGTTGCCATTGCTACAGGCATCGTGGTGTCACGCTCGTCGTTTGGTATGGCTTCATTCAGCTCCGGTTCCCAACGATCAAGGCGAGTTACATGATCCCCCATGTTGTGCAAAAAAGCGGTTAGCTCCTTCGGTCCTCCGATCGTTGTCAGAAGTAAGTTGGCCGCAGTGTTATCACTCATGGTTATGGCAGCACTGCATAATTCTCTTACTGTCATGCCATCCGTAAGATGCTTTTCTGTGACTGGTGAGTACTCAACCAAGTCATTCTGAGAATAGTGTATGCGGCGACCGAGTTGCTCTTGCCCGGCGTCAATACGGGATAATACCGCGCCACATAGCAGAACTTTAAAAGTGCTCATCATTGGAAAACGTTCTTCGGGGCGAAAACTCTCAAGGATCTTACCGCTGTTGAGATCCAGTTCGATGTAACCCACTCGTGCACCCAACTGATCTTCAGCATCTTTTACTTTCACCAGCGTTTCTGGGTGAGCAAAAACAGGAAGGCAAAATGCCGCAAAAAAGGGAATAAGGGCGACACGGAAATGTTGAATACTCATACTCTTCCTTTTTCAATATTATTGAAGCATTTATCAGGGTTATTGTCTCATGAGCGGATACATATTTGAATGTATTTAGAAAAATAAACAAATAGGGGTTCCGCGCACATTTCCCCGAAAAGTGCCACCTGACGTCTAAGAAACCATTATTATCATGACATTAACCTATAAAAATAGGCGTATCACGAGGCCCTTTCGTCTCGCGCGTTTCGGTGATGACGGTGAAAACCTCTGACACATGCAGCTCCCGGAGACGGTCACAGCTTGTCTGTAAGCGGATGCCGGGAGCAGACAAGCCCGTCAGGGCGCGTCAGCGGGTGTTGGCGGGTGTCGGGGCTGGCTTAACTATGCGGCATCAGAGCAGATTGTACTGAGAGTGCACCATATGCGGTGTGAAATACCGCACCGAATCGCGCGGAACTAACGACAGTCGCTCCAAGGTCGTCGAACAAAAGGTGAATGTGTTGCGGAGAGCGGGTGGGAGACAGCGAAAGAGCAACTACGAAACGTGGTGTGGTGGAGGTGAATTATGAAGAGGGCGCGCGATTTGAAAAGTATGTATATAAAAAATATATCCCGGTGTTTTATGTAGCGATAAACGAGTTTTTGATGTAAGGTATGCAGGTGTGTAAGTCTTTTGGTTAGAAGACAAATCCAAAGTCTACTTGTGGGGATGTTCGAAGGGGAAATACTTGTATTCTATAGGTCATATCTTGTTTTTATTGGCACAAATATAATTACATTAGCTTTTTGAGGGGGCAATAAACAGTAAACACGATGGTAATAATGGTAAAAAAAAAAACAAGCAGTTATTTCGGATATATGTCGGCTACTCCTTGCGTCGGGCCCGAAGTCTTAGAGCCAGATATGCGAGCACCCGGAAGCTCACGATGAGAATGGCCAGACCATGATGAAATAACATAAGGTGGTCCCGTCGGCAAGAGACATCCACTTAACGTATGCTTGCAATAAGTGCGAGTGAAAGGAATAGTATTCTGAGTGTCGTATTGAGTCTGAGTGAGACAGCGATATGATTGTTGATTAACCCTTAGCATGTCCGTGGGGTTTGAATTAACTCATAATATTAATTAGACGAAATTATTTTTAAAGTTTTATTTTTAATAATTTGCGAGTACGCA

**pCaSepR-attB-gypsy-HSPmini-IVS-syn21-nlsGFP-PEST-2A-nlsRFP-p10-gypsy**

GGCTGCAGGTCGACCTCGAGGCCTCGAGTTAACGTTACGTTAACGTTAACGTTCGAGGTCGACTCTAGTTGGCCACGTAATAAGTGTGCGTTGAATTTATTCGCAAAAACATTGCATATTTTCGGCAAAGTAAAATTTTGTTGCATACCTTATCAAAAAATAAGTGCTGCATACTTTTTAGAGAAACCAAATAATTTTTTATTGCATACCCGTTTTTAATAAAATACATTGCATACCCTCTTTTAATAAAAAATATTGCATACTTTGACGAAACAAATTTTCGTTGCATACCCAATAAAAGATTATTATATTGCATACCCGTTTTTAATAAAATACATTGCATACCCTCTTTTAATAAARAATATTGCATACGTTGACGAAACAAATTTTCGTTGCATACCCAATAAAAGATTATTATATTGCATACCTTTTCTTGCCATACCATTTAGCCGATCAATTGTGCTCGGCAACAGTATATTTGTGGTGTGCCAACCAACAACTCTAGAGGATCCAGATCCACTAGCGAGCGGAGACTCTAGCGCTAGCGACGTCGAGCGCCGGAGTATAAATAGAGGCGCTTCGTCTACGGAGCGACAATTCAATTCAAACAAGCAAAGTGAACACGTCGCTAAGCGAAAGCTAAGCAAATAAACAAGCGCAGCTGAACAAGCTAAACAATCTGCAGTAAAGTGCAAGTTAAAGTGAATCAATTAAAAGTAACCAGCAACCAAGTAAATCAACTGCAACTACTGAAATCTGCCAAGAAGTAATTATTGAATACAAGAAGAGAACTCTGAATAGATCTAAAAGGTAGGTTCAACCACTGATGCCTAGGCACACCGAAACGACTAACCCTAATTCTTATCCTTTACTTCAGAACTTAAAAAAAAAAATCAAAATGCCCAAGAAGAAGCGCAAGGTGAGATCCCGTAAGTACACCTCGTGGTACGTCGCCCTGAAGCGCGTGTCCAAGGGCGAGGAGCTGTTCACCGGCGTGGTGCCCATCCTGGTGGAGCTGGATGGCGACGTGAACGGCCACAAGTTCAGCGTGCGCGGCGAGGGCGAGGGCGACGCCACCAACGGCAAGCTGACCCTGAAGTTCATCTGCACCACCGGCAAGCTGCCCGTGCCCTGGCCCACCCTGGTGACCACCCTGACCTACGGCGTGCAGTGCTTCAGCCGCTACCCCGATCACATGAAGCAGCACGATTTCTTCAAGAGCGCCATGCCCGAGGGCTACGTGCAGGAGCGCACCATCAGCTTCAAGGATGACGGCACCTACAAGACCCGCGCCGAGGTGAAGTTCGAGGGCGATACCCTGGTGAACCGCATCGAGCTGAAGGGCATCGATTTCAAGGAGGATGGCAACATCCTGGGCCACAAGCTGGAGTACAACTTCAACAGCCACAACGTGTACATCACCGCCGATAAGCAGAAGAACGGCATCAAGGCCAACTTCAAGATCCGCCACAATGTGGAGGATGGCTCCGTGCAGCTGGCCGATCACTACCAGCAGAACACCCCCATCGGCGACGGCCCAGTGCTGCTGCCCGATAACCACTACCTGAGCACCCAGAGCGTGCTGTCCAAGGACCCCAACGAGAAGCGCGATCACATGGTGCTGCTGGAGTTCGTGACCGCCGCCGGCATCACCCTGGGCATGGATGAGCTGTACAAGACTAGTTACAAGAAGCTTAGCCATGGCTTCCCGCCGGAGGTGGAGGAGCAGGATGATGGCACGCTGCCCATGTCTTGTGCCCAGGAGAGCGGGATGGACCGTCACCCTGCAGCCTGTGCTTCTGCTAGGATCAATGTGGAGGGCAGAGGAAGTCTTCTAACATGCGGTGACGTGGAGGAGAATCCCGGCCCTATGCCCAAGAAGAAGCGCAAGGTGCGCTCCCGTAAGTACACCTCCTGGTACGTCGCCCTGAAGCGCGTGTCCAAGGGCGAGGAGCTGATCAAGGAGAACATGCACATGAAGCTGTACATGGAGGGCACCGTGAACAACCACCACTTCAAGTGCACCAGCGAGGGCGAGGGCAAGCCCTACGAGGGCACCCAGACCATGCGCATCAAGGTGGTGGAGGGCGGCCCACTGCCCTTCGCCTTCGATATCCTGGCCACCTCCTTCATGTACGGCAGCCGCACCTTCATCAACCACACCCAGGGCATCCCCGATTTCTTCAAGCAGAGCTTCCCCGAGGGCTTCACCTGGGAGCGCGTGACCACCTACGAGGATGGCGGCGTGCTGACCGCCACCCAGGATACCAGCCTGCAGGATGGCTGCCTGATCTACAACGTGAAGATCCGCGGCGTGAACTTCCCCAGCAACGGCCCCGTGATGCAGAAGAAGACCCTGGGCTGGGAGGCCAACACCGAGATGCTGTACCCCGCCGATGGCGGCCTGGAGGGCCGCACCGATATGGCCCTGAAGCTGGTCGGCGGCGGCCACCTGATCTGCAACTTCAAGACCACCTACCGCAGCAAGAAGCCCGCCAAGAACCTGAAGATGCCCGGCGTGTACTACGTGGATCACCGCCTGGAGCGCATTAAGGAGGCCGATAAGGAGACCTACGTCGAGCAGCACGAGGTGGCCGTGGCCCGCTACTGCGATCTGCCGTCCAAGCTGGGACACAAGCTGAACGGCATGGATGAGCTGTACAAGACTAGTTAATAGAATGAATCGTTTTTAAAATAACAAATCAATTGTTTTATAATATTCGTACGATTCTTTGATTATGTAATAAAATGTGATCATTAGGAAGATTACGAAAAATATAAAAAATATGAGTTCTGTGTGTATAACAAATGCTGTAAACGCCACAATTGTGTTTGTTGCAAATAAACCCATGATTATTTGATTAAAATTGTTGTTTTCTTTGTTCATAGACAATAGTGTGTTTTGCCTAAACGTGTACTGCATAAACTCCATGCGAGTGTATAGCGAGCTAGTGGCTAACGCTTGCCCCACCAAAGTAGATTCGTCAAAATCCTCAATTTCATCACCCTCCTCCAAGTTTAACATTTGGCCGTCGGAATTAACTTCTAAAGATGCCACATAATCTAATAAATGAAATAGAGATTCAAACGTGGCGTCATCGTCCGTTTCGACCATTTCCGAAAAGAACTCGGGCATAAACTCTATGATTTCTCTGGACGTGGTGTTGTCGAAACTCTCAAAGTACGCAGTCAGGAACGTGCGCGACATGTCGTCGGGAAACTCGCGCGGAAACATGTTGTTGTAACCGAACGGGTCCCATAGCGCCAAAACCAAATCTGCCAGCGTCAATAGAATGAGCACGATGCCGACAATGGAGCTGGCTTGGATAGCGATTCGAGTTAACGGCCGGCCGAACTTGTTTATTGCAGCTTATAATGGTTACAAATAAAGCAATAGCATCACAAATTTCACAAATAAAGCATTTTTTTCACTGCATTCTAGTTGTGGTTTGTCCAAACTCATCAATGTATCTTATCATGTCTGGTTCCAGAGCTCTGGCCACGTAATAAGTGTGCGTTGAATTTATTCGCAAAAACATTGCATATTTTCGGCAAAGTAAAATTTTGTTGCATACCTTATCAAAAAATAAGTGCTGCATACTTTTTAGAGAAACCAAATAATTTTTTATTGCATACCCGTTTTTAATAAAATACATTGCATACCCTCTTTTAATAAAAAATATTGCATACTTTGACGAAACAAATTTTCGTTGCATACCCAATAAAAGATTATTATATTGCATACCCGTTTTTAATAAAATACATTGCATACCCTCTTTTAATAAAAAATATTGCATACGTTGACGAAACAAATTTTCGTTGCATACCCAATAAAAGATTATTATATTGCATACCTTTTCTTGCCATACCATTTAGCCGATCAATTGTGCTCGGCAACAGTATATTTGTGGTGTGCCAACCAACAACGAGCTCCAGCTTTTGTTCCCTTTAGTGAGGGTTAATTTCGAGCTTGGCGTAATCATGGTCATAGCTGTTTCCTGTGTGAAATTGTTATCCGCTCACAATTCCACACAACATACGAGCCGGAAGCATAAAGTGTAAAGCCTGGGGTGCCTAATGAGTGAGCTAACTCACATTAATTGCGTTGCGCTCACTGCCCGCTTTCCAGTCGGGAAACCTGTCGTGCCAGCTGCATTAATGAATCGGCCAACGCGCGGGGAGAGGCGGTTTGCGTATTGGGCGCTCTTCCGCTTCCTCGCTCACTGACTCGCTGCGCTCGGTCGTTCGGCTGCGGCGAGCGGTATCAGCTCACTCAAAGGCGGTAATACGGTTATCCACAGAATCAGGGGATAACGCAGGAAAGAACATGTGAGCAAAAGGCCAGCAAAAGGCCAGGAACCGTAAAAAGGCCGCGTTGCTGGCGTTTTTCCATAGGCTCCGCCCCCCTGACGAGCATCACAAAAATCGACGCTCAAGTCAGAGGTGGCGAAACCCGACAGGACTATAAAGATACCAGGCGTTTCCCCCTGGAAGCTCCCTCGTGCGCTCTCCTGTTCCGACCCTGCCGCTTACCGGATACCTGTCCGCCTTTCTCCCTTCGGGAAGCGTGGCGCTTTCTCATAGCTCACGCTGTAGGTATCTCAGTTCGGTGTAGGTCGTTCGCTCCAAGCTGGGCTGTGTGCACGAACCCCCCGTTCAGCCCGACCGCTGCGCCTTATCCGGTAACTATCGTCTTGAGTCCAACCCGGTAAGACACGACTTATCGCCACTGGCAGCAGCCACTGGTAACAGGATTAGCAGAGCGAGGTATGTAGGCGGTGCTACAGAGTTCTTGAAGTGGTGGCCTAACTACGGCTACACTAGAAGGACAGTATTTGGTATCTGCGCTCTGCTGAAGCCAGTTACCTTCGGAAAAAGAGTTGGTAGCTCTTGATCCGGCAAACAAACCACCGCTGGTAGCGGTGGTTTTTTTGTTTGCAAGCAGCAGATTACGCGCAGAAAAAAAGGATCTCAAGAAGATCCTTTGATCTTTTCTACGGGGTCTGACGCTCAGTGGAACGAAAACTCACGTTAAGGGATTTTGGTCATGAGATTATCAAAAAGGATCTTCACCTAGATCCTTTTAAATTAAAAATGAAGTTTTAAATCAATCTAAAGTATATATGAGTAAACTTGGTCTGACAGTTACCAATGCTTAATCAGTGAGGCACCTATCTCAGCGATCTGTCTATTTCGTTCATCCATAGTTGCCTGACTCCCCGTCGTGTAGATAACTACGATACGGGAGGGCTTACCATCTGGCCCCAGTGCTGCAATGATACCGCGAGACCCACGCTCACCGGCTCCAGATTTATCAGCAATAAACCAGCCAGCCGGAAGGGCCGAGCGCAGAAGTGGTCCTGCAACTTTATCCGCCTCCATCCAGTCTATTAATTGTTGCCGGGAAGCTAGAGTAAGTAGTTCGCCAGTTAATAGTTTGCGCAACGTTGTTGCCATTGCTACAGGCATCGTGGTGTCACGCTCGTCGTTTGGTATGGCTTCATTCAGCTCCGGTTCCCAACGATCAAGGCGAGTTACATGATCCCCCATGTTGTGCAAAAAAGCGGTTAGCTCCTTCGGTCCTCCGATCGTTGTCAGAAGTAAGTTGGCCGCAGTGTTATCACTCATGGTTATGGCAGCACTGCATAATTCTCTTACTGTCATGCCATCCGTAAGATGCTTTTCTGTGACTGGTGAGTACTCAACCAAGTCATTCTGAGAATAGTGTATGCGGCGACCGAGTTGCTCTTGCCCGGCGTCAATACGGGATAATACCGCGCCACATAGCAGAACTTTAAAAGTGCTCATCATTGGAAAACGTTCTTCGGGGCGAAAACTCTCAAGGATCTTACCGCTGTTGAGATCCAGTTCGATGTAACCCACTCGTGCACCCAACTGATCTTCAGCATCTTTTACTTTCACCAGCGTTTCTGGGTGAGCAAAAACAGGAAGGCAAAATGCCGCAAAAAAGGGAATAAGGGCGACACGGAAATGTTGAATACTCATACTCTTCCTTTTTCAATATTATTGAAGCATTTATCAGGGTTATTGTCTCATGAGCGGATACATATTTGAATGTATTTAGAAAAATAAACAAATAGGGGTTCCGCGCACATTTCCCCGAAAAGTGCCACCTAAATTGTAAGCGTTAATATTTTGTTAAAATTCGCGTTAAATTTTTGTTAAATCAGCTCATTTTTTAACCAATAGGCCGAAATCGGCAAAATCCCTTATAAATCAAAAGAATAGACCGAGATAGGGTTGAGTGTTGTTCCAGTTTGGAACAAGAGTCCACTATTAAAGAACGTGGACTCCAACGTCAAAGGGCGAAAAACCGTCTATCAGGGCGATGGCCCACTACGTGAACCATCACCCTAATCAAGTTTTTTGGGGTCGAGGTGCCGTAAAGCACTAAATCGGAACCCTAAAGGGAGCCCCCGATTTAGAGCTTGACGGGGAAAGCCGGCGAACGTGGCGAGAAAGGAAGGGAAGAAAGCGAAAGGAGCGGGCGCTAGGGCGCTGGCAAGTGTAGCGGTCACGCTGCGCGTAACCACCACACCCGCCGCGCTTAATGCGCCGCTACAGGGCGCGTCCCATTCGCCATTCAGGCTGCGCAACTGTTGGGAAGGGCGATCGGTGCGGGCCTCTTCGCTATTACGCCAGCTGGCGAAAGGGGGATGTGCTGCAAGGCGATTAAGTTGGGTAACGCCAGGGTTTTCCCAGTCACGACGTTGTAAAACGACGGCCAGTGAATTGTAATACGACTCACTATAGGGCGAATTGGGTACAAGCTTCACGACCTGAGGCGCGCCTCCAGTGAAATCCAAGCATTTTCTAAATTAAATGTATTCTTATTATTATAGTTGTTATTTTTGATATATATAAACAACACTATTATGCCCACCATTTTTTTGAGATGCATCTACACAAGGAACAAACACTGGATGTCACTTTCAGTTCAAATTGTAACGCTAATCACTCCGAACAGGTCACAAAAAATTACCTTAAAAAGTCATAATATTAAATTAGAATAAATATAGCTGTGAGGGAAATATATACAAATATATTGGAGCAAATAAATTGTACATACAAATATTTATTACTAATTTCTATTGAGACGAAATGAACCACTCGGAACCATTTGAGCGAACCGAATCGCGCGGAACTAACGACAGTCGCTCCAAGGTCGTCGAACAAAAGGTGAATGTGTTGCGGAGAGCGGGTGGGAGACAGCGAAAGAGCAACTACGAAACGTGGTGTGGTGGAGGTGAATTATGAAGAGGGCGCGCGATTTGAAAAGTATGTATATAAAAAATATATCCCGGTGTTTTATGTAGCGATAAACGAGTTTTTGATGTAAGGTATGCAGGTGTGTAAGTCTTTTGGTTAGAAGACAAATCCAAAGTCTACTTGTGGGGATGTTCGAAGGGGAAATACTTGTATTCTATAGGTCATATCTTGTTTTTATTGGCACAAATATAATTACATTAGCTTTTTGAGGGGGCAATAAACAGTAAACACGATGGTAATAATGGTAAAAAAAAAAACAAGCAGTTATTTCGGATATATGTCGGCTACTCCTTGCGTCGGGCCCGAAGTCTTAGAGCCAGATATGCGAGCACCCGGAAGCTCACGATGAGAATGGCCAGACCCACGTAGTCCAGCGGCAGATCGGCGGCGGAGAAGTTAAGCGTCTCCAGGATGACCTTGCCCGAACTGGGGCACGTGGTGTTCGACGATGTGCAGCTAATTTCGCCCGGCTCCACGTCCGCCCATTGGTTAATCAGCAGACCCTCGTTGGCGTAACGGAACCATGAGAGGTACGACAACCATTTGAGGTATACTGGCACCGAGCCCGAGTTCAAGAAGAAGCCGCCAAAGAGCAGGAATGGTATGATAACCGGCGGACCCACAGACAGCGCCATCGAGGTCGAGGAGCTGGCGCAGGATATTAGATATCCGAAGGACGTTGACACATTGGCCACCAGAGTGACCAGCGCCAGGCAGTTGAAGAAGTGCAGCACTCCGGCCCGCAGTCCGATCATCGGATAGGCAATCGCCGTGAAGACCAGTGGCACTGTGAGAAAAAGCGGTAATTCGGCAATCGTTTTGCCCAGAAAGTATGTGTCACAGCGATAAAGTCGACTTCGGGCCTCCCTCATAAAAACTGGCAGCTCTGAGGTGAACACCTAAATCGAATCGATTCATTAGAAAGTTAGTAAATTATTAATATGCAAATGTATTCTAAACAAGACTTACATTTATCGTGGCAAAGACGTTTTGAAAGGTCATGTTGGTCAGGAAGAGGAAGATGGCTCCGTTGATATTCATCACGCCCACTTGCGTGAGTTGTTGGCCCAAAAAGATGAGGCCAATCAAGATGGCAACCATCTGCAAATTAAAATGTTACTCGCATCTCATTAATATTCATATCTTCAACATGTTCGCGAGTTAAATGAAATTTATTTATTTTCTGCAAAACTATAAACTATACATCTCATTGAAAAAAACTAAGAAGGGTGTGGAATCAGGCAATTCTAACTAAAATCTAGCGAATTTGTTTCCAAGAATTGTAAGCGTTATATCATTTGTTTCCACTGGAACCACTCACCGTTGTCTGAATAAGTCGCACTTTTACGAGGAGTGGTTCCTTGAGCACCGACAGCCAGGATCGCCACAGGACCGCCCGGAACTGCATGAACCAGGTGGCCTTGTAGGTGTACCCATTCTCCGGCTGCTCCAGTGGCTTCTCCAAATTTTTGGTGGCCAACAACTGCTCCATATCCCGGGCTACTTTGCTAATAGCAAAATTGTCGCATATCTTGGCGATCCGATCACGGGACTCGATCTCCCGTCCGGGCACAACGGCCAACACCTGTACGTAAAAGTCCGCCGGATTGTAGTTGGTAGGACACTGGGCACCCACGCTGGATAGGAGTTGAGATGTAATGTAATGCTAGATACCCTTAATAAACACATCGAACTCACTAGGAAAAGAAGTCGACGGCTTCGCTGGGAGTGCCCAAGAAAGCTACCCTGCCCTCGGCCATCAGAAGGATCTTGTCAAAGAGCTCAAACAGCTCGGAAGACGGCTGATGAATGGTCAGGATGACGGTCTTGCCCTTCTGCGACAGCTTCTTCAGCACCTGGACGACGCTGTGGGCGGTAAATGAGTCCAGTCCGGAGGTGGGCTCATCGCAGATCAGAAGCGGCGGATCGGTTAGTGCCTCGGAGGCGAATGCCAGACGCTTCCTTTCTCCGCCGGACAGACCTTTCACCCTGCCGGGCACACCGATGATCGTGTGCTGACATTTGCTGAGCGAAAGCTCCTGGATCACCTGATCCACGCGGGCCACTCGCTGCCGATAGGTCAGATGTCGTGGCATCCGCACCATGGCCTGGAAAATCAGGTGTTCCCTGGCCGTTAGGGAGCCGATAAAGAGGTCATCCTGCTGGACATAGGCGCACCTGGCCTGCATCTCCTTGGCGTCCACAGGTTGGCCATTGAGCAGTCGCATCCCGGATGGCGATACTTGGATGCCCTGCGGCGATCGAAAGGCAAGGGCATTCAGCAGGGTCGTCTTTCCGGCACCGGAACTGCCCATCACGGCCAAAAGTTCGCCCGGATAGGCCACGCCGCAAACTGAGTTTCAAATTGGTAATTGGACCCTTTATTAAGATTTCACACAGATCAGCCGACTGCGAATAGAAACTCACCGTTCTTGAGCAAATGTTTCCTGGGCGCCGGTATGTGTCGCTCGTTGCAGAATAGTCCGCGTGTCCGGTTGACCAGCTGCCGCCATCCGGAGCCCGGCTGATTGACCGCCCCAAAGATGTCCATATTGTGCCAGGCATAGGTGAGGTTCTCGGCTAGTTGGCCGCTCCCTGAACCGGAGTCCTCCGGCGGACTGGGTGGCAGGAGCGTGCCGTAGTTTTTGGCCTGCCCGAAGCCCTGGTTAATGCAGCTCTGCGAAGCCGCTCCGCTGTCACCCTGCAATGATAGGGGATCTCAAATATCAACTACAAGCGTTATGCTCATCTAACCCCGAACAAAACGAAGTATCCTACGAAGTAGGTTTATACTTTTATTTATTTTTTGTGCATCTAGGATCAGCTTAAAATATCTGGTTGTTATATTTTTTGTAAAAAAGAATGTAGTCGAAAATGAATGCCTTTAGATGTCTTGATCATGATATGATCTTAAAAATTGTCTTATATAGCGAGCACAGCTACCAGAATAATCTGTTTCGTGTCACTATTTGTTTGTGCGATTGCGGTTTGGGATTTTTGTGGGTCGCAGTTCTCACGCCGCAGACAATTTGATGTTGCAATCGCAGTTCCTATAGATCAAGTGAACTTAAGATGTATGCACATGTACTACTCACATTGTTCAGATGCTCGGCAGATGGGTGTTTGCTGCCTCCGCGAATTAATAGCTCCTGATCCTCTTGGCCCATTGCCGGGATTTTTCACACTTTCCCCTGCTTACCCACCCAAAACCAATCACCACCCCAATCACTCAAAAAACAAACAAAAATAAGAAGCGAGAGGAGTTTTGGCACAGCACTTTGTGTTTAATTGATGGCGTAAACCGCTTGGAGCTTCGTCACGAAACCGCTGACAAAGTGCAACTGAAGGCGGACATTGACGCTAGGTAACGCTACAAACGGTGGCGAAAGAGATAGCGGACGCAGCGGCGAAAGAGACGGCGATATTTCTGTGGACAGAGAAGGAGGCAAACAGCGCTGACTTTGAGTGGAATGTCATTTTGAGTGAGAGGTAATCGAAAGAACCTGGTACTTCAAATACCCTTGGATCGAAGTAAATTTAAAACTGATCAGATAAGTTCAATGATATCCAGTGCAGTAAAAAATAAAAAAAAAAAATGTTTTTTTTTATCTACTTTCCGCAAAAATGGGTTTTATTAACTTACATACATGGCGCGCCAGATCGCAAGAAGCTTGATATCATCGATCTCGAGGCTGCATCCAACGCGTTGGGAGCTCTCCGGATCAATTCGGCTTCAGGTACCGTCGACGATGTAGGTCACGGTCTCGAAGCCGCGGTGCGGGTGCCAGGGCGTGCCCTTGGGCTCCCCGGGCGCGTACTCCACCTCACCCATCTGGTCCATCATGATGAACGGGTCGAGGTGGCGGTAGTTGATCCCGGCGAACGCGCGGCGCACCGGGAAGCCCTCGCCCTCGAAACCGCTGGGCGCGGTGGTCACGGTGAGCACGGGACGTGCGACGGCGTCGGCGGGTGCGGATACGCGGGGCAGCGTCAGCGGGTTCTCGACGGTCACGGCGGGCATGTCGACAAGCCGAATTGATCCACTAGAAGG

**pUAST-IVS-syn21-nlsGFP-PEST-2A-nlsRFP-p10:**

GCCTAATTCGGTACACTAGTTGGCCACGTAATAAGTGTGCGTTGAATTTATTCGCAAAAACATTGCATATTTTCGGCAAAGTAAAATTTTGTTGCATACCTTATCAAAAAATAAGTGCTGCATACTTTTTAGAGAAACCAAATAATTTTTTATTGCATACCCGTTTTTAATAAAATACATTGCATACCCTCTTTTAATAAAAAATATTGCATACTTTGACGAAACAAATTTTCGTTGCATACCCAATAAAAGATTATTATATTGCATACCCGTTTTTAATAAAATACATTGCATACCCTCTTTTAATAAAAAATATTGCATACGTTGACGAAACAAATTTTCGTTGCATACCCAATAAAAGATTATTATATTGCATACCTTTTCTTGCCATACCATTTAGCCGATCAATTGTGCTCGGCAACAGTATATTTGTGGTGTGCCAACCAACAACACTAGTAGTACCAGCTTATAACTTCGTATAATGTATGCTATACGAAGTTATCTGCAGGCAGGTCGGAGTACTGTCCTCCGAGCGGAGTACTGTCCTCCGAGCGGAGTACTGTCCTCCGAGCGGAGTACTGTCCTCCGAGCGGAGTACTGTCCTCCGAGCGGAGACTCCCATGGATAACTTCGTATAATGTATGCTATACGAAGTTATGGATCCGCAGGTCGGAGTACTGTCCTCCGAGCGGAGTACTGTCCTCCGAGCGGAGTACTGTCCTCCGAGCGGAGTACTGTCCTCCGAGCGGAGTACTGTCCTCCGAGCGGAGACTCGTCGACAGTCGAGCGCCGGAGTATAAATAGAGGCGCTTCGTCTACGGAGCGACAATTCAATTCAAACAAGCAAAGTGAACACGTCGCTAAGCGAAAGCTAAGCAAATAAACAAGCGCAGCTGAACAAGCTAAACAATCTGCAGTAAAGTGCAAGTTAAAGTGAATCAATTAAAAGTAACCAGCAACCAAGTAAATCAACTGCAACTACTGAAATCTGCCAAGAAGTAATTATTGAATACAAGAAGAGAACTCTGAATAGATCTAAAAGGTAGGTTCAACCACTGATGCCTAGGCACACCGAAACGACTAACCCTAATTCTTATCCTTTACTTCAGAACTTAAAAAAAAAAATCAAAATGCCCAAGAAGAAGCGCAAGGTGAGATCCCGTAAGTACACCTCGTGGTACGTCGCCCTGAAGCGCGTGTCCAAGGGCGAGGAGCTGTTCACCGGCGTGGTGCCCATCCTGGTGGAGCTGGATGGCGACGTGAACGGCCACAAGTTCAGCGTGCGCGGCGAGGGCGAGGGCGACGCCACCAACGGCAAGCTGACCCTGAAGTTCATCTGCACCACCGGCAAGCTGCCCGTGCCCTGGCCCACCCTGGTGACCACCCTGACCTACGGCGTGCAGTGCTTCAGCCGCTACCCCGATCACATGAAGCAGCACGATTTCTTCAAGAGCGCCATGCCCGAGGGCTACGTGCAGGAGCGCACCATCAGCTTCAAGGATGACGGCACCTACAAGACCCGCGCCGAGGTGAAGTTCGAGGGCGATACCCTGGTGAACCGCATCGAGCTGAAGGGCATCGATTTCAAGGAGGATGGCAACATCCTGGGCCACAAGCTGGAGTACAACTTCAACAGCCACAACGTGTACATCACCGCCGATAAGCAGAAGAACGGCATCAAGGCCAACTTCAAGATCCGCCACAATGTGGAGGATGGCTCCGTGCAGCTGGCCGATCACTACCAGCAGAACACCCCCATCGGCGACGGCCCAGTGCTGCTGCCCGATAACCACTACCTGAGCACCCAGAGCGTGCTGTCCAAGGACCCCAACGAGAAGCGCGATCACATGGTGCTGCTGGAGTTCGTGACCGCCGCCGGCATCACCCTGGGCATGGATGAGCTGTACAAGACTAGTTACAAGAAGCTTAGCCATGGCTTCCCGCCGGAGGTGGAGGAGCAGGATGATGGCACGCTGCCCATGTCTTGTGCCCAGGAGAGCGGGATGGACCGTCACCCTGCAGCCTGTGCTTCTGCTAGGATCAATGTGGAGGGCAGAGGAAGTCTTCTAACATGCGGTGACGTGGAGGAGAATCCCGGCCCTATGCCCAAGAAGAAGCGCAAGGTGCGCTCCCGTAAGTACACCTCCTGGTACGTCGCCCTGAAGCGCGTGTCCAAGGGCGAGGAGCTGATCAAGGAGAACATGCACATGAAGCTGTACATGGAGGGCACCGTGAACAACCACCACTTCAAGTGCACCAGCGAGGGCGAGGGCAAGCCCTACGAGGGCACCCAGACCATGCGCATCAAGGTGGTGGAGGGCGGCCCACTGCCCTTCGCCTTCGATATCCTGGCCACCTCCTTCATGTACGGCAGCCGCACCTTCATCAACCACACCCAGGGCATCCCCGATTTCTTCAAGCAGAGCTTCCCCGAGGGCTTCACCTGGGAGCGCGTGACCACCTACGAGGATGGCGGCGTGCTGACCGCCACCCAGGATACCAGCCTGCAGGATGGCTGCCTGATCTACAACGTGAAGATCCGCGGCGTGAACTTCCCCAGCAACGGCCCCGTGATGCAGAAGAAGACCCTGGGCTGGGAGGCCAACACCGAGATGCTGTACCCCGCCGATGGCGGCCTGGAGGGCCGCACCGATATGGCCCTGAAGCTGGTCGGCGGCGGCCACCTGATCTGCAACTTCAAGACCACCTACCGCAGCAAGAAGCCCGCCAAGAACCTGAAGATGCCCGGCGTGTACTACGTGGATCACCGCCTGGAGCGCATTAAGGAGGCCGATAAGGAGACCTACGTCGAGCAGCACGAGGTGGCCGTGGCCCGCTACTGCGATCTGCCGTCCAAGCTGGGACACAAGCTGAACGGCATGGATGAGCTGTACAAGACTAGTTAATAGAATGAATCGTTTTTAAAATAACAAATCAATTGTTTTATAATATTCGTACGATTCTTTGATTATGTAATAAAATGTGATCATTAGGAAGATTACGAAAAATATAAAAAATATGAGTTCTGTGTGTATAACAAATGCTGTAAACGCCACAATTGTGTTTGTTGCAAATAAACCCATGATTATTTGATTAAAATTGTTGTTTTCTTTGTTCATAGACAATAGTGTGTTTTGCCTAAACGTGTACTGCATAAACTCCATGCGAGTGTATAGCGAGCTAGTGGCTAACGCTTGCCCCACCAAAGTAGATTCGTCAAAATCCTCAATTTCATCACCCTCCTCCAAGTTTAACATTTGGCCGTCGGAATTAACTTCTAAAGATGCCACATAATCTAATAAATGAAATAGAGATTCAAACGTGGCGTCATCGTCCGTTTCGACCATTTCCGAAAAGAACTCGGGCATAAACTCTATGATTTCTCTGGACGTGGTGTTGTCGAAACTCTCAAAGTACGCAGTCAGGAACGTGCGCGACATGTCGTCGGGAAACTCGCGCGGAAACATGTTGTTGTAACCGAACGGGTCCCATAGCGCCAAAACCAAATCTGCCAGCGTCAATAGAATGAGCACGATGCCGACAATGGAGCTGGCTTGGATAGCGATTCGAGTTAACGGCCGGCCGAACTTGTTTATTGCAGCTTATAATGGTTACAAATAAAGCAATAGCATCACAAATTTCACAAATAAAGCATTTTTTTCACTGCATTCTAGTTGTGGTTTGTCCAAACTCATCAATGTATCTTATCATGTCTGGTTCCAGAGCTCTGGCCACGTAATAAGTGTGCGTTGAATTTATTCGCAAAAACATTGCATATTTTCGGCAAAGTAAAATTTTGTTGCATACCTTATCAAAAAATAAGTGCTGCATACTTTTTAGAGAAACCAAATAATTTTTTATTGCATACCCGTTTTTAATAAAATACATTGCATACCCTCTTTTAATAAAAAATATTGCATACTTTGACGAAACAAATTTTCGTTGCATACCCAATAAAAGATTATTATATTGCATACCCGTTTTTAATAAAATACATTGCATACCCTCTTTTAATAAAAAATATTGCATACGTTGACGAAACAAATTTTCGTTGCATACCCAATAAAAGATTATTATATTGCATACCTTTTCTTGCCATACCATTTAGCCGATCAATTGTGCTCGGCAACAGTATATTTGTGGTGTGCCAACCAACAACGAGCTCCAGCTTTTGTTCCCTTTAGTGAGGGTTAATTTCGAGCTTGGCGTAATCATGGTCATAGCTGTTTCCTGTGTGAAATTGTTATCCGCTCACAATTCCACACAACATACGAGCCGGAAGCATAAAGTGTAAAGCCTGGGGTGCCTAATGAGTGAGCTAACTCACATTAATTGCGTTGCGCTCACTGCCCGCTTTCCAGTCGGGAAACCTGTCGTGCCAGCTGCATTAATGAATCGGCCAACGCGCGGGGAGAGGCGGTTTGCGTATTGGGCGCTCTTCCGCTTCCTCGCTCACTGACTCGCTGCGCTCGGTCGTTCGGCTGCGGCGAGCGGTATCAGCTCACTCAAAGGCGGTAATACGGTTATCCACAGAATCAGGGGATAACGCAGGAAAGAACATGTGAGCAAAAGGCCAGCAAAAGGCCAGGAACCGTAAAAAGGCCGCGTTGCTGGCGTTTTTCCATAGGCTCCGCCCCCCTGACGAGCATCACAAAAATCGACGCTCAAGTCAGAGGTGGCGAAACCCGACAGGACTATAAAGATACCAGGCGTTTCCCCCTGGAAGCTCCCTCGTGCGCTCTCCTGTTCCGACCCTGCCGCTTACCGGATACCTGTCCGCCTTTCTCCCTTCGGGAAGCGTGGCGCTTTCTCATAGCTCACGCTGTAGGTATCTCAGTTCGGTGTAGGTCGTTCGCTCCAAGCTGGGCTGTGTGCACGAACCCCCCGTTCAGCCCGACCGCTGCGCCTTATCCGGTAACTATCGTCTTGAGTCCAACCCGGTAAGACACGACTTATCGCCACTGGCAGCAGCCACTGGTAACAGGATTAGCAGAGCGAGGTATGTAGGCGGTGCTACAGAGTTCTTGAAGTGGTGGCCTAACTACGGCTACACTAGAAGGACAGTATTTGGTATCTGCGCTCTGCTGAAGCCAGTTACCTTCGGAAAAAGAGTTGGTAGCTCTTGATCCGGCAAACAAACCACCGCTGGTAGCGGTGGTTTTTTTGTTTGCAAGCAGCAGATTACGCGCAGAAAAAAAGGATCTCAAGAAGATCCTTTGATCTTTTCTACGGGGTCTGACGCTCAGTGGAACGAAAACTCACGTTAAGGGATTTTGGTCATGAGATTATCAAAAAGGATCTTCACCTAGATCCTTTTAAATTAAAAATGAAGTTTTAAATCAATCTAAAGTATATATGAGTAAACTTGGTCTGACAGTTACCAATGCTTAATCAGTGAGGCACCTATCTCAGCGATCTGTCTATTTCGTTCATCCATAGTTGCCTGACTCCCCGTCGTGTAGATAACTACGATACGGGAGGGCTTACCATCTGGCCCCAGTGCTGCAATGATACCGCGAGACCCACGCTCACCGGCTCCAGATTTATCAGCAATAAACCAGCCAGCCGGAAGGGCCGAGCGCAGAAGTGGTCCTGCAACTTTATCCGCCTCCATCCAGTCTATTAATTGTTGCCGGGAAGCTAGAGTAAGTAGTTCGCCAGTTAATAGTTTGCGCAACGTTGTTGCCATTGCTACAGGCATCGTGGTGTCACGCTCGTCGTTTGGTATGGCTTCATTCAGCTCCGGTTCCCAACGATCAAGGCGAGTTACATGATCCCCCATGTTGTGCAAAAAAGCGGTTAGCTCCTTCGGTCCTCCGATCGTTGTCAGAAGTAAGTTGGCCGCAGTGTTATCACTCATGGTTATGGCAGCACTGCATAATTCTCTTACTGTCATGCCATCCGTAAGATGCTTTTCTGTGACTGGTGAGTACTCAACCAAGTCATTCTGAGAATAGTGTATGCGGCGACCGAGTTGCTCTTGCCCGGCGTCAATACGGGATAATACCGCGCCACATAGCAGAACTTTAAAAGTGCTCATCATTGGAAAACGTTCTTCGGGGCGAAAACTCTCAAGGATCTTACCGCTGTTGAGATCCAGTTCGATGTAACCCACTCGTGCACCCAACTGATCTTCAGCATCTTTTACTTTCACCAGCGTTTCTGGGTGAGCAAAAACAGGAAGGCAAAATGCCGCAAAAAAGGGAATAAGGGCGACACGGAAATGTTGAATACTCATACTCTTCCTTTTTCAATATTATTGAAGCATTTATCAGGGTTATTGTCTCATGAGCGGATACATATTTGAATGTATTTAGAAAAATAAACAAATAGGGGTTCCGCGCACATTTCCCCGAAAAGTGCCACCTAAATTGTAAGCGTTAATATTTTGTTAAAATTCGCGTTAAATTTTTGTTAAATCAGCTCATTTTTTAACCAATAGGCCGAAATCGGCAAAATCCCTTATAAATCAAAAGAATAGACCGAGATAGGGTTGAGTGTTGTTCCAGTTTGGAACAAGAGTCCACTATTAAAGAACGTGGACTCCAACGTCAAAGGGCGAAAAACCGTCTATCAGGGCGATGGCCCACTACGTGAACCATCACCCTAATCAAGTTTTTTGGGGTCGAGGTGCCGTAAAGCACTAAATCGGAACCCTAAAGGGAGCCCCCGATTTAGAGCTTGACGGGGAAAGCCGGCGAACGTGGCGAGAAAGGAAGGGAAGAAAGCGAAAGGAGCGGGCGCTAGGGCGCTGGCAAGTGTAGCGGTCACGCTGCGCGTAACCACCACACCCGCCGCGCTTAATGCGCCGCTACAGGGCGCGTCCCATTCGCCATTCAGGCTGCGCAACTGTTGGGAAGGGCGATCGGTGCGGGCCTCTTCGCTATTACGCCAGCTGGCGAAAGGGGGATGTGCTGCAAGGCGATTAAGTTGGGTAACGCCAGGGTTTTCCCAGTCACGACGTTGTAAAACGACGGCCAGTGAATTGTAATACGACTCACTATAGGGCGAATTGGGTACAAGCTTCACGACCTGAGGCGCGCCTCCAGTGAAATCCAAGCATTTTCTAAATTAAATGTATTCTTATTATTATAGTTGTTATTTTTGATATATATAAACAACACTATTATGCCCACCATTTTTTTGAGATGCATCTACACAAGGAACAAACACTGGATGTCACTTTCAGTTCAAATTGTAACGCTAATCACTCCGAACAGGTCACAAAAAATTACCTTAAAAAGTCATAATATTAAATTAGAATAAATATAGCTGTGAGGGAAATATATACAAATATATTGGAGCAAATAAATTGTACATACAAATATTTATTACTAATTTCTATTGAGACGAAATGAACCACTCGGAACCATTTGAGCGAACCGAATCGCGCGGAACTAACGACAGTCGCTCCAAGGTCGTCGAACAAAAGGTGAATGTGTTGCGGAGAGCGGGTGGGAGACAGCGAAAGAGCAACTACGAAACGTGGTGTGGTGGAGGTGAATTATGAAGAGGGCGCGCGATTTGAAAAGTATGTATATAAAAAATATATCCCGGTGTTTTATGTAGCGATAAACGAGTTTTTGATGTAAGGTATGCAGGTGTGTAAGTCTTTTGGTTAGAAGACAAATCCAAAGTCTACTTGTGGGGATGTTCGAAGGGGAAATACTTGTATTCTATAGGTCATATCTTGTTTTTATTGGCACAAATATAATTACATTAGCTTTTTGAGGGGGCAATAAACAGTAAACACGATGGTAATAATGGTAAAAAAAAAAACAAGCAGTTATTTCGGATATATGTCGGCTACTCCTTGCGTCGGGCCCGAAGTCTTAGAGCCAGATATGCGAGCACCCGGAAGCTCACGATGAGAATGGCCAGACCCACGTAGTCCAGCGGCAGATCGGCGGCGGAGAAGTTAAGCGTCTCCAGGATGACCTTGCCCGAACTGGGGCACGTGGTGTTCGACGATGTGCAGCTAATTTCGCCCGGCTCCACGTCCGCCCATTGGTTAATCAGCAGACCCTCGTTGGCGTAACGGAACCATGAGAGGTACGACAACCATTTGAGGTATACTGGCACCGAGCCCGAGTTCAAGAAGAAGCCGCCAAAGAGCAGGAATGGTATGATAACCGGCGGACCCACAGACAGCGCCATCGAGGTCGAGGAGCTGGCGCAGGATATTAGATATCCGAAGGACGTTGACACATTGGCCACCAGAGTGACCAGCGCCAGGCAGTTGAAGAAGTGCAGCACTCCGGCCCGCAGTCCGATCATCGGATAGGCAATCGCCGTGAAGACCAGTGGCACTGTGAGAAAAAGCGGTAATTCGGCAATCGTTTTGCCCAGAAAGTATGTGTCACAGCGATAAAGTCGACTTCGGGCCTCCCTCATAAAAACTGGCAGCTCTGAGGTGAACACCTAAATCGAATCGATTCATTAGAAAGTTAGTAAATTATTAATATGCAAATGTATTCTAAACAAGACTTACATTTATCGTGGCAAAGACGTTTTGAAAGGTCATGTTGGTCAGGAAGAGGAAGATGGCTCCGTTGATATTCATCACGCCCACTTGCGTGAGTTGTTGGCCCAAAAAGATGAGGCCAATCAAGATGGCAACCATCTGCAAATTAAAATGTTACTCGCATCTCATTAATATTCATATCTTCAACATGTTCGCGAGTTAAATGAAATTTATTTATTTTCTGCAAAACTATAAACTATACATCTCATTGAAAAAAACTAAGAAGGGTGTGGAATCAGGCAATTCTAACTAAAATCTAGCGAATTTGTTTCCAAGAATTGTAAGCGTTATATCATTTGTTTCCACTGGAACCACTCACCGTTGTCTGAATAAGTCGCACTTTTACGAGGAGTGGTTCCTTGAGCACCGACAGCCAGGATCGCCACAGGACCGCCCGGAACTGCATGAACCAGGTGGCCTTGTAGGTGTACCCATTCTCCGGCTGCTCCAGTGGCTTCTCCAAATTTTTGGTGGCCAACAACTGCTCCATATCCCGGGCTACTTTGCTAATAGCAAAATTGTCGCATATCTTGGCGATCCGATCACGGGACTCGATCTCCCGTCCGGGCACAACGGCCAACACCTGTACGTAAAAGTCCGCCGGATTGTAGTTGGTAGGACACTGGGCACCCACGCTGGATAGGAGTTGAGATGTAATGTAATGCTAGATACCCTTAATAAACACATCGAACTCACTAGGAAAAGAAGTCGACGGCTTCGCTGGGAGTGCCCAAGAAAGCTACCCTGCCCTCGGCCATCAGAAGGATCTTGTCAAAGAGCTCAAACAGCTCGGAAGACGGCTGATGAATGGTCAGGATGACGGTCTTGCCCTTCTGCGACAGCTTCTTCAGCACCTGGACGACGCTGTGGGCGGTAAATGAGTCCAGTCCGGAGGTGGGCTCATCGCAGATCAGAAGCGGCGGATCGGTTAGTGCCTCGGAGGCGAATGCCAGACGCTTCCTTTCTCCGCCGGACAGACCTTTCACCCTGCCGGGCACACCGATGATCGTGTGCTGACATTTGCTGAGCGAAAGCTCCTGGATCACCTGATCCACGCGGGCCACTCGCTGCCGATAGGTCAGATGTCGTGGCATCCGCACCATGGCCTGGAAAATCAGGTGTTCCCTGGCCGTTAGGGAGCCGATAAAGAGGTCATCCTGCTGGACATAGGCGCACCTGGCCTGCATCTCCTTGGCGTCCACAGGTTGGCCATTGAGCAGTCGCATCCCGGATGGCGATACTTGGATGCCCTGCGGCGATCGAAAGGCAAGGGCATTCAGCAGGGTCGTCTTTCCGGCACCGGAACTGCCCATCACGGCCAAAAGTTCGCCCGGATAGGCCACGCCGCAAACTGAGTTTCAAATTGGTAATTGGACCCTTTATTAAGATTTCACACAGATCAGCCGACTGCGAATAGAAACTCACCGTTCTTGAGCAAATGTTTCCTGGGCGCCGGTATGTGTCGCTCGTTGCAGAATAGTCCGCGTGTCCGGTTGACCAGCTGCCGCCATCCGGAGCCCGGCTGATTGACCGCCCCAAAGATGTCCATATTGTGCCAGGCATAGGTGAGGTTCTCGGCTAGTTGGCCGCTCCCTGAACCGGAGTCCTCCGGCGGACTGGGTGGCAGGAGCGTGCCGTAGTTTTTGGCCTGCCCGAAGCCCTGGTTAATGCAGCTCTGCGAAGCCGCTCCGCTGTCACCCTGCAATGATAGGGGATCTCAAATATCAACTACAAGCGTTATGCTCATCTAACCCCGAACAAAACGAAGTATCCTACGAAGTAGGTTTATACTTTTATTTATTTTTTGTGCATCTAGGATCAGCTTAAAATATCTGGTTGTTATATTTTTTGTAAAAAAGAATGTAGTCGAAAATGAATGCCTTTAGATGTCTTGATCATGATATGATCTTAAAAATTGTCTTATATAGCGAGCACAGCTACCAGAATAATCTGTTTCGTGTCACTATTTGTTTGTGCGATTGCGGTTTGGGATTTTTGTGGGTCGCAGTTCTCACGCCGCAGACAATTTGATGTTGCAATCGCAGTTCCTATAGATCAAGTGAACTTAAGATGTATGCACATGTACTACTCACATTGTTCAGATGCTCGGCAGATGGGTGTTTGCTGCCTCCGCGAATTAATAGCTCCTGATCCTCTTGGCCCATTGCCGGGATTTTTCACACTTTCCCCTGCTTACCCACCCAAAACCAATCACCACCCCAATCACTCAAAAAACAAACAAAAATAAGAAGCGAGAGGAGTTTTGGCACAGCACTTTGTGTTTAATTGATGGCGTAAACCGCTTGGAGCTTCGTCACGAAACCGCTGACAAAGTGCAACTGAAGGCGGACATTGACGCTAGGTAACGCTACAAACGGTGGCGAAAGAGATAGCGGACGCAGCGGCGAAAGAGACGGCGATATTTCTGTGGACAGAGAAGGAGGCAAACAGCGCTGACTTTGAGTGGAATGTCATTTTGAGTGAGAGGTAATCGAAAGAACCTGGTACTTCAAATACCCTTGGATCGAAGTAAATTTAAAACTGATCAGATAAGTTCAATGATATCCAGTGCAGTAAAAAATAAAAAAAAAAAATGTTTTTTTTTATCTACTTTCCGCAAAAATGGGTTTTATTAACTTACATACATGGCGCGCCAGATCGCAAGAAGCTTGATATCATCGATCTCGAGGCTGCATCCAACGCGTTGGGAGCTCTCCGGATCAATTCGGCTTCAGGTACCGTCGACGATGTAGGTCACGGTCTCGAAGCCGCGGTGCGGGTGCCAGGGCGTGCCCTTGGGCTCCCCGGGCGCGTACTCCACCTCACCCATCTGGTCCATCATGATGAACGGGTCGAGGTGGCGGTAGTTGATCCCGGCGAACGCGCGGCGCACCGGGAAGCCCTCGCCCTCGAAACCGCTGGGCGCGGTGGTCACGGTGAGCACGGGACGTGCGACGGCGTCGGCGGGTGCGGATACGCGGGGCAGCGTCAGCGGGTTCTCGACGGTCACGGCGGGCATGTCGACAAGCCGAATTGATCCACTAGAAGG

**pCaSepR4-DSCP:: ISV-syn21-nlsGFP-3XMyc-PEST-2A-nlsRFP-3XHA-p10**

CAAATAATGATTTTATTTTGACTGATAGTGACCTGTTCGTTGCAACACATTGATGAGCAATGCTTTTTTATAATGCCAACTTTGTACAAAAAAGCAGGCTTCAGCGGAGACTCTAGCGAGCTCGCCCGGGGATCGAGCGCAGCGGTATAAAAGGGCGCGGGGTGGCTGAGAGCATCAGTTGTGAATGAATGTTCGAGCCGAGCAGACGTGCCGCTGCCTTCGTTAATATCCTTTGAATAAGCCAACTTTGAATCACAAGACGCATACCAAACGAACTCGATGATCTAAAAGGTAGGTTCAACCACTGATGCCTAGGCACACCGAAACGACTAACCCTAATTCTTATCCTTTACTTCAGAACTTAAAAAAAAAAATCAAAATGCCCAAGAAGAAGCGCAAGGTGAGATCCCGTAAGTACACCTCGTGGTACGTCGCCCTGAAGCGCGTGTCCAAGGGCGAGGAGCTGTTCACCGGCGTGGTGCCCATCCTGGTGGAGCTGGATGGCGACGTGAACGGCCACAAGTTCAGCGTGCGCGGCGAGGGCGAGGGCGACGCCACCAACGGCAAGCTGACCCTGAAGTTCATCTGCACCACCGGCAAGCTGCCCGTGCCCTGGCCCACCCTGGTGACCACCCTGACCTACGGCGTGCAGTGCTTCAGCCGCTACCCCGATCACATGAAGCAGCACGATTTCTTCAAGAGCGCCATGCCCGAGGGCTACGTGCAGGAGCGCACCATCAGCTTCAAGGATGACGGCACCTACAAGACCCGCGCCGAGGTGAAGTTCGAGGGCGATACCCTGGTGAACCGCATCGAGCTGAAGGGCATCGATTTCAAGGAGGATGGCAACATCCTGGGCCACAAGCTGGAGTACAACTTCAACAGCCACAACGTGTACATCACCGCCGATAAGCAGAAGAACGGCATCAAGGCCAACTTCAAGATCCGCCACAATGTGGAGGATGGCTCCGTGCAGCTGGCCGATCACTACCAGCAGAACACCCCCATCGGCGACGGCCCAGTGCTGCTGCCCGATAACCACTACCTGAGCACCCAGAGCGTGCTGTCCAAGGACCCCAACGAGAAGCGCGATCACATGGTGCTGCTGGAGTTCGTGACCGCCGCCGGCATCACCCTGGGCATGGATGAGCTGTACAAGACTAGTCTCGAGAACGGTGGTTCAGAGCAGAAGCTGATTAGCGAGGAGGACCTGGGCGAGCAGAAGCTGATTTCGGAGGAGGACCTCGGTGAGCAGAAGCTGATTTCCGAAGAGGACCTGAGCGGCTCCGGACACGGTTTCCCCCCAGAGGTGGAGGAGCAGGACGATGGTACCCTGCCCATGTCGTGCGCCCAGGAATCCGGCATGGACCGTCACCCAGCCGCCTGCGCCTCCGCTCGCATTAACGTCGGCTCCGGAACCGGTTCCGGTGCCACTAACTTCAGCCTGTTGAAGCAGGCCGGCGACGTGGAGGAGAATCCAGGACCACGCTCGCCCAAGAAGAAGATGCCCAAGAAGAAGCGCAAGGTGGTGTCCAAGGGCGAGGAGCTGATCAAGGAGAACATGCACATGAAGCTGTACATGGAGGGCACCGTGAACAACCACCACTTCAAGTGCACCAGCGAGGGCGAGGGCAAGCCCTACGAGGGCACCCAGACCATGCGCATCAAGGTGGTGGAGGGCGGCCCACTGCCCTTCGCCTTCGATATCCTGGCCACCTCCTTCATGTACGGCAGCCGCACCTTCATCAACCACACCCAGGGCATCCCCGATTTCTTCAAGCAGAGCTTCCCCGAGGGCTTCACCTGGGAGCGCGTGACCACCTACGAGGATGGCGGCGTGCTGACCGCCACCCAGGATACCAGCCTGCAGGATGGCTGCCTGATCTACAACGTGAAGATCCGCGGCGTGAACTTCCCCAGCAACGGCCCCGTGATGCAGAAGAAGACCCTGGGCTGGGAGGCCAACACCGAGATGCTGTACCCCGCCGATGGCGGCCTGGAGGGCCGCACCGATATGGCCCTGAAGCTGGTCGGCGGCGGCCACCTGATCTGCAACTTCAAGACCACCTACCGCAGCAAGAAGCCCGCCAAGAACCTGAAGATGCCCGGCGTGTACTACGTGGATCACCGCCTGGAGCGCATTAAGGAGGCCGATAAGGAGACCTACGTCGAGCAGCACGAGGTGGCCGTGGCCCGCTACTGCGATCTGCCGTCCAAGCTGGGACACAAGCTGAACGGCATGGATGAGCTGTACAAGACTAGTCAGAGTGGAAGCTACCCCTACGATGTTCCCGATTACGCTGGCTACCCCTACGATGTCCCCGATTACGCCGGATCCTACCCCTATGATGTCCCCGACTACGCCTAAGAGCTGTACTAGTTAATAGAATGAATCGTTTTTAAAATAACAAATCAATTGTTTTATAATATTCGTACGATTCTTTGATTATGTAATAAAATGTGATCATTAGGAAGATTACGAAAAATATAAAAAATATGAGTTCTGTGTGTATAACAAATGCTGTAAACGCCACAATTGTGTTTGTTGCAAATAAACCCATGATTATTTGATTAAAATTGTTGTTTTCTTTGTTCATAGACAATAGTGTGTTTTGCCTAAACGTGTACTGCATAAACTCCATGCGAGTGTATAGCGAGCTAGTGGCTAACGCTTGCCCCACCAAAGTAGATTCGTCAAAATCCTCAATTTCATCACCCTCCTCCAAGTTTAACATTTGGCCGTCGGAATTAACTTCTAAAGATGCCACATAATCTAATAAATGAAATAGAGATTCAAACGTGGCGTCATCGTCCGTTTCGACCATTTCCGAAAAGAACTCGGGCATAAACTCTATGATTTCTCTGGACGTGGTGTTGTCGAAACTCTCAAAGTACGCAGTCAGGAACGTGCGCGACATGTCGTCGGGAAACTCGCGCGGAAACATGTTGTTGTAACCGAACGGGTCCCATAGCGCCAAAACCAAATCTGCCAGCGTCAATAGAATGAGCACGATGCCGACAATGGAGCTGGCTTGGATAGCGATTCGAGTTAACGGCCGGCCGATTCGAGTTAACGGCCGGCTAGTTGGCCACGTAAGATCCCACCCAGCTTTCTTGTACAAAGTGGTGAGCTCCGCCACCATGGAGCAAAAGCTCATTTCTGAAGAGGACTTGAATGAAATGGAGCAAAAGCTCATTTCTGAAGAGGACTTGAATGAAATGGAGCAAAAGCTCATTTCTGAAGAGGACTTGAATGAAATGGAGCAAAAGCTCATTTCTGAAGAGGACTTGAATGAAATGGAGCAAAAGCTCATTTCTGAAGAGGACTTGAATGAAATGGAGAGCTTGGGCGACCTCACCATGGAGCAAAAGCTCATTTCTGAAGAGGACTTGAATCACCGGTCCACGTGACGTAAGCTAGAGGATCTTTGTGAAGGAACCTTACTTCTGTGGTGTGACATAATTGGACATCGAGGCCTCGAGTTAACGTTACGTTAACGTTAACGTTCGAGGTCGACTCTAGAGGATCCAGATCCACTAGTGGCCTATGCGGCCGCGGATCTGATCCCGGGCGGGTACCGAATTCTAGTATGTATGTAAGTTAATAAAACCCTTTTTTGGAGAATGTAGATTTAAAAAAACATATTTTTTTTTTATTTTTTACTGCACTGGATATCATTGAACTTATCTGATCAGTTTTAAATTTACTTCGATCCAAGGGTATTTGAAGTACCAGGTTCTTTCGATTACCTCTCACTCAAAATGACATTCCACTCAAAGTCAGCGCTGTTTGCCTCCTTCTCTGTCCACAGAAATATCGCCGTCTCTTTCGCCGCTGCGTCCGCTATCTCTTTCGCCACCGTTTGTAGCGTTACCTAGCGTCAATGTCCGCCTTCAGTTGCACTTTGTCAGCGGTTTCGTGACGAAGCTCCAAGCGGTTTACGCCATCAATTAAACACAAAGTGCTGTGCCAAAACTCCTCTCGCTTCTTATTTTTGTTTGTTTTTTGAGTGATTGGGGTGGTGATTGGTTTTGGGTGGGTAAGCAGGGGAAAGTGTGAAAAATCCCGGCAATGGGCCAAGAGGATCAGGAGCTATTAATTCGCGGAGGCAGCAAACACCCATCTGCCGAGCATCTGAACAATGTGAGTAGTACATGTGCATACATCTTAAGTTCACTTGATCTATAGGAACTGCGATTGCAACATCAAATTGTCTGCGGCGTGAGAACTGCGACCCACAAAAATCCCAAACCGCAATCGCACAAACAAATAGTGACACGAAACAGATTATTCTGGTAGCTGTGCTCGCTATATAAGACAATTTTTAAGATCATATCATGATCAAGACATCTAAAGGCATTCATTTTCGACTACATTCTTTTTTACAAAAAATATAACAACCAGATATTTTAAGCTGATCCTAGATGCACAAAAAATAAATAAAAGTATAAACCTACTTCGTAGGATACTTCGTTTTGTTCGGGGTTAGATGAGCATAACGCTTGTAGTTGATATTTGAGATCCCCTATCATTGCAGGGTGACAGCGGACGCTTCGCAGAGCTGCATTAACCAGGGCTTCGGGCAGGCCAAAAACTACGGCACGCTCCTGCCACCCAGTCCGCCGGAGGACTCCGGTTCAGGGAGCGGCCAACTAGCCGAGAACCTCACCTATGCCTGGCACAATATGGACATCTTTGGGGCGGTCAATCAGCCGGGCTCCGGATGGCGGCAGCTGGTCAACCGGACACGCGGACTATTCTGCAACGAGCGACACATACCGGCGCCCAGGAAACATTTGCTCAAGAACGGTGAGTTTCTATTCGCAGTCGGCTGATCTGTGTGAAATCTTAATAAAGGGTCCAATTACCAATTTGAAACTCAGTTTGCGGCGTGGCCTATCCGGGCGAACTTTTGGCCGTGATGGGCAGTTCCGGTGCCGGAAAGACGACCCTGCTGAATGCCCTTGCCTTTCGATCGCCGCAGGGCATCCAAGTATCGCCATCCGGGATGCGACTGCTCAATGGCCAACCTGTGGACGCCAAGGAGATGCAGGCCAGGTGCGCCTATGTCCAGCAGGATGACCTCTTTATCGGCTCCCTAACGGCCAGGGAACACCTGATTTTCCAGGCCATGGTGCGGATGCCACGACATCTGACCTATCGGCAGCGAGTGGCCCGCGTGGATCAGGTGATCCAGGAGCTTTCGCTCAGCAAATGTCAGCACACGATCATCGGTGTGCCCGGCAGGGTGAAAGGTCTGTCCGGCGGAGAAAGGAAGCGTCTGGCATTCGCCTCCGAGGCACTAACCGATCCGCCGCTTCTGATCTGCGATGAGCCCACCTCCGGACTGGACTCATTTACCGCCCACAGCGTCGTCCAGGTGCTGAAGAAGCTGTCGCAGAAGGGCAAGACCGTCATCCTGACCATTCATCAGCCGTCTTCCGAGCTGTTTGAGCTCTTTGACAAGATCCTTCTGATGGCCGAGGGCAGGGTAGCTTTCTTGGGCACTCCCAGCGAAGCCGTCGACTTCTTTTCCTAGTGAGTTCGATGTGTTTATTAAGGGTATCTAGCATTACATTACATCTCAACTCCTATCCAGCGTGGGTGCCCAGTGTCCTACCAACTACAATCCGGCGGACTTTTACGTACAGGTGTTGGCCGTTGTGCCCGGACGGGAGATCGAGTCCCGTGATCGGATCGCCAAGATATGGCGACAATTTTGCTATTAGCAAAGTAGCCCGGGATATGGAGCAGTTGTTGGCCACCAAAAATTTGGAGAAGCCACTGGAGCAGCCGGAGAATGGGTACACCTACAAGGCCACCTGGTTCATGCAGTTCCGGGCGGTCCTGTGGCGATCCTGGCTGTCGGTGCTCAAGGAACCACTCCTCGTAAAAGTGCGACTTATTCAGACAACGGTGAGTGGTTCCAGTGGAAACAAATGATATAACGCTTACAATTCTTGGAAACAAATTCGCTAGATTTTAGTTAGAATTGCCTGATTCCACACCCTTCTTAGTTTTTTTCAATGAGATGTATAGTTTATAGTTTTGCAGAAAATAAATAAATTTCATTTAACTCGCGAACATGTTGAAGATATGAATATTAATGAGATGCGAGTAACATTTTAATTTGCAGATGGTTGCCATCTTGATTGGCCTCATCTTTTTGGGCCAACAACTCACGCAAGTGGGCGTGATGAATATCAACGGAGCCATCTTCCTCTTCCTGACCAACATGACCTTTCAAAACGTCTTTGCCACGATAAATGTAAGTCTTGTTTAGAATACATTTGCATATTAATAATTTACTAACTTTCTAATGAATCGATTCGATTTAGGTGTTCACCTCAGAGCTGCCAGTTTTTATGAGGGAGGCCCGAAGTCGACTTTATCGCTGTGACACATACTTTCTGGGCAAAACGATTGCCGAATTACCGCTTTTTCTCACAGTGCCACTGGTCTTCACGGCGATTGCCTATCCGATGATCGGACTGCGGGCCGGAGTGCTGCACTTCTTCAACTGCCTGGCGCTGGTCACTCTGGTGGCCAATGTGTCAACGTCCTTCGGATATCTAATATCCTGCGCCAGCTCCTCGACCTCGATGGCGCTGTCTGTGGGTCCGCCGGTTATCATACCATTCCTGCTCTTTGGCGGCTTCTTCTTGAACTCGGGCTCGGTGCCAGTATACCTCAAATGGTTGTCGTACCTCTCATGGTTCCGTTACGCCAACGAGGGTCTGCTGATTAACCAATGGGCGGACGTGGAGCCGGGCGAAATTAGCTGCACATCGTCGAACACCACGTGCCCCAGTTCGGGCAAGGTCATCCTGGAGACGCTTAACTTCTCCGCCGCCGATCTGCCGCTGGACTACGTGGGTCTGGCCATTCTCATCGTGAGCTTCCGGGTGCTCGCATATCTGGCTCTAAGACTTCGGGCCCGACGCAAGGAGTAGCCGACATATATCCGAAATAACTGCTTGTTTTTTTTTTTACCATTATTACCATCGTGTTTACTGTTTATTGCCCCCTCAAAAAGCTAATGTAATTATATTTGTGCCAATAAAAACAAGATATGACCTATAGAATACAAGTATTTCCCCTTCGAACATCCCCACAAGTAGACTTTGGATTTGTCTTCTAACCAAAAGACTTACACACCTGCATACCTTACATCAAAAACTCGTTTATCGCTACATAAAACACCGGGATATATTTTTTATATACATACTTTTCAAATCGCGCGCCCTCTTCATAATTCACCTCCACCACACCACGTTTCGTAGTTGCTCTTTCGCTGTCTCCCACCCGCTCTCCGCAACACATTCACCTTTTGTTCGACGACCTTGGAGCGACTGTCGTTAGTTCCGCGCGATTCGGTTCGCTCAAATGGTTCCGAGTGGTTCATTTCGTCTCAATAGAAATTAGTAATAAATATTTGTATGTACAATTTATTTGCTCCAATATATTTGTATATATTTCCCTCACAGCTATATTTATTCTAATTTAATATTATGACTTTTTAAGGTAATTTTTTGTGACCTGTTCGGAGTGATTAGCGTTACAATTTGAACTGAAAGTGACATCCAGTGTTTGTTCCTTGTGTAGATGCATCTCAAAAAAATGGTGGGCATAATAGTGTTGTTTATATATATCAAAAATAACAACTATAATAATAAGAATACATTTAATTTAGAAAATGCTTGGATTTCACTGGAACTAGAATTAATTCGGCTGCTGCTCTAAACGACGCATTTCGTACTCCAAAGTACGAATTTTTTCCCTCAAGCTCTTATTTTCATTAAACAATGAACAGGACCTAACGCACAGTCACGTTATTGTTTACATAAATGATTTTTTTTACTATTCAAACTTACTCTGTTTGTGTACTCCCACTGGTATAGCCTTCTTTTATCTTTTCTGGTTCAGGCTCTATCACTTTACTAGGTACGGCATCTGCGTTGAGTCGCCTCCTTTTAAATGTCTGACCTTTTGCAGGTGCAGCCTTCCACTGCGAATCTTTAAAGTGGGTATCACAAATTTGGGAGTTTTCACCAAGGCTGCACCCAAGGCTCTGCTCCCACAATTTTCTCTTAATAGCACACTTCGGCACGTGAATTAATTTTACTCCAGTCACAGCTTTGCAGCAAAATTTGCAATATTTCATTTTTTTTTATTCCACGTAAGGGTTAATGTTTTCAAAAAAAAATTCGTCCGCACACAACCTTTCCTCTCAACAAGCAAACGTGCACTGAATTTAAGTGTATACTTCGGTAAGCTTCGGCTATCGACGGGACCACCTTATGTTATTTCATCATGGGCCAGACCCACGTAGTCCAGCGGCAGATCGGCGGCGGAGAAGTTAAGCGTCTCCAGGATGACCTTGCCCGAACTGGGGCACGTGGTGTTCGACGATGTGCAGCTAATTTCGCCCGGCTCCACGTCCGCCCATTGGTTAATCAGCAGACCCTCGTTGGCGTAACGGAACCATGAGAGGTACGACAACCATTTGAGGTATACTGGCACCGAGCCCGAGTTCAAGAAGAAGGCGTTTTTCCATAGGCTCCGCCCCCCTGACGAGCATCACAAAAATCGACGCTCAAGTCAGAGGTGGCGAAACCCGACAGGACTATAAAGATACCAGGCGTTTCCCCCTGGAAGCTCCCTCGTGCGCTCTCCTGTTCCGACCCTGCCGCTTACCGGATACCTGTCCGCCTTTCTCCCTTCGGGAAGCGTGGCGCTTTCTCAATGCTCACGCTGTAGGTATCTCAGTTCGGTGTAGGTCGTTCGCTCCAAGCTGGGCTGTGTGCACGAACCCCCCGTTCAGCCCGACCGCTGCGCCTTATCCGGTAACTATCGTCTTGAGTCCAACCCGGTAAGACACGACTTATCGCCACTGGCAGCAGCCACTGGTAACAGGATTAGCAGAGCGAGGTATGTAGGCGGTGCTACAGAGTTCTTGAAGTGGTGGCCTAACTACGGCTACACTAGAAGGACAGTATTTGGTATCTGCGCTCTGCTGAAGCCAGTTACCTTCGGAAAAAGAGTTGGTAGCTCTTGATCCGGCAAACAAACCACCGCTGGTAGCGGTGGTTTTTTTGTTTGCAAGCAGCAGATTACGCGCAGAAAAAAAGGATCTCAAGAAGATCCTTTGATCTTTTCTACGGGGTCTGACGCTCAGTGGAACGAAAACTCACGTTAAGGGATTTTGGTCATGAGATTATCAAAAAGGATCTTCACCTAGATCCTTTTAAATTAAAAATGAAGTTTTAAATCAATCTAAAGTATATATGAGTAAACTTGGTCTGACAGTTACCAATGCTTAATCAGTGAGGCACCTATCTCAGCGATCTGTCTATTTCGTTCATCCATAGTTGCCTGACTCCCCGTCGTGTAGATAACTACGATACGGGAGGGCTTACCATCTGGCCCCAGTGCTGCAATGATACCGCGAGACCCACGCTCACCGGCTCCAGATTTATCAGCAATAAACCAGCCAGCCGGAAGGGCCGAGCGCAGAAGTGGTCCTGCAACTTTATCCGCCTCCATCCAGTCTATTAATTGTTGCCGGGAAGCTAGAGTAAGTAGTTCGCCAGTTAATAGTTTGCGCAACGTTGTTGCCATTGCTACAGGCATCGTGGTGTCACGCTCGTCGTTTGGTATGGCTTCATTCAGCTCCGGTTCCCAACGATCAAGGCGAGTTACATGATCCCCCATGTTGTGCAAAAAAGCGGTTAGCTCCTTCGGTCCTCCGATCGTTGTCAGAAGTAAGTTGGCCGCAGTGTTATCACTCATGGTTATGGCAGCACTGCATAATTCTCTTACTGTCATGCCATCCGTAAGATGCTTTTCTGTGACTGGTGAGTACTCAACCAAGTCATTCTGAGAATAGTGTATGCGGCGACCGAGTTGCTCTTGCCCGGCGTCAATACGGGATAATACCGCGCCACATAGCAGAACTTTAAAAGTGCTCATCATTGGAAAACGTTCTTCGGGGCGAAAACTCTCAAGGATCTTACCGCTGTTGAGATCCAGTTCGATGTAACCCACTCGTGCACCCAACTGATCTTCAGCATCTTTTACTTTCACCAGCGTTTCTGGGTGAGCAAAAACAGGAAGGCAAAATGCCGCAAAAAAGGGAATAAGGGCGACACGGAAATGTTGAATACTCATACTCTTCCTTTTTCAATATTATTGAAGCATTTATCAGGGTTATTGTCTCATGAGCGGATACATATTTGAATGTATTTAGAAAAATAAACAAATAGGGGTTCCGCGCACATTTCCCCGAAAAGTGCCACCTGACGTCTAAGAAACCATTATTATCATGACATTAACCTATAAAAATAGGCGTATCACGAGGCCCTTTCGTCTCGCGCGTTTCGGTGATGACGGTGAAAACCTCTGACACATGCAGCTCCCGGAGACGGTCACAGCTTGTCTGTAAGCGGATGCCGGGAGCAGACAAGCCCGTCAGGGCGCGTCAGCGGGTGTTGGCGGGTGTCGGGGCTGGCTTAACTATGCGGCATCAGAGCAGATTGTACTGAGAGTGCACCATATGCGGTGTGAAATACCGCACCGAATCGCGCGGAACTAACGACAGTCGCTCCAAGGTCGTCGAACAAAAGGTGAATGTGTTGCGGAGAGCGGGTGGGAGACAGCGAAAGAGCAACTACGAAACGTGGTGTGGTGGAGGTGAATTATGAAGAGGGCGCGCGATTTGAAAAGTATGTATATAAAAAATATATCCCGGTGTTTTATGTAGCGATAAACGAGTTTTTGATGTAAGGTATGCAGGTGTGTAAGTCTTTTGGTTAGAAGACAAATCCAAAGTCTACTTGTGGGGATGTTCGAAGGGGAAATACTTGTATTCTATAGGTCATATCTTGTTTTTATTGGCACAAATATAATTACATTAGCTTTTTGAGGGGGCAATAAACAGTAAACACGATGGTAATAATGGTAAAAAAAAAAACAAGCAGTTATTTCGGATATATGTCGGCTACTCCTTGCGTCGGGCCCGAAGTCTTAGAGCCAGATATGCGAGCACCCGGAAGCTCACGATGAGAATGGCCAGACCATGATGAAATAACATAAGGTGGTCCCGTCGGCAAGAGACATCCACTTAACGTATGCTTGCAATAAGTGCGAGTGAAAGGAATAGTATTCTGAGTGTCGTATTGAGTCTGAGTGAGACAGCGATATGATTGTTGATTAACCCTTAGCATGTCCGTGGGGTTTGAATTAACTCATAATATTAATTAGACGAAATTATTTTTAAAGTTTTATTTTTAATAATTTGCGAGTACGCA

**pCaSepR4-DSCP:: ISV-syn21-nlsGFP-3XMyc-PEST-2A-lexA-p10 insert:**

CAAATAATGATTTTATTTTGACTGATAGTGACCTGTTCGTTGCAACACATTGATGAGCAATGCTTTTTTATAATGCCAACTTTGTACAAAAAAGCAGGCTTCAGCGGAGACTCTCGAGCGCCGGAGTATAAATAGAGGCGCTTCGTCTACGGAGCGACAATTCAATTCAAACAAGCAAAGTGAACACGTCGCTAAGCGAAAGCTAAGCAAATAAACAAGCGCAGCTGAACAAGCTAAACAATCTGCAGTAAAGTGCAAGTTAAAGTGAATCAATTAAAAGTAACCAGCAACCAAGTAAATCAACTGCAACTACTGAAATCTGCCAAGAAGTAATTATTGAATACAAGAAGAGAACTCTGAATGATGATCTAAAAGGTAGGTTCAACCACTGATGCCTAGGCACACCGAAACGACTAACCCTAATTCTTATCCTTTACTTCAGAACTTAAAAAAAAAAATCAAAATGCCCAAGAAGAAGCGCAAGGTGAGATCCCGTAAGTACACCTCGTGGTACGTCGCCCTGAAGCGCGTGTCCAAGGGCGAGGAGCTGTTCACCGGCGTGGTGCCCATCCTGGTGGAGCTGGATGGCGACGTGAACGGCCACAAGTTCAGCGTGCGCGGCGAGGGCGAGGGCGACGCCACCAACGGCAAGCTGACCCTGAAGTTCATCTGCACCACCGGCAAGCTGCCCGTGCCCTGGCCCACCCTGGTGACCACCCTGACCTACGGCGTGCAGTGCTTCAGCCGCTACCCCGATCACATGAAGCAGCACGATTTCTTCAAGAGCGCCATGCCCGAGGGCTACGTGCAGGAGCGCACCATCAGCTTCAAGGATGACGGCACCTACAAGACCCGCGCCGAGGTGAAGTTCGAGGGCGATACCCTGGTGAACCGCATCGAGCTGAAGGGCATCGATTTCAAGGAGGATGGCAACATCCTGGGCCACAAGCTGGAGTACAACTTCAACAGCCACAACGTGTACATCACCGCCGATAAGCAGAAGAACGGCATCAAGGCCAACTTCAAGATCCGCCACAATGTGGAGGATGGCTCCGTGCAGCTGGCCGATCACTACCAGCAGAACACCCCCATCGGCGACGGCCCAGTGCTGCTGCCCGATAACCACTACCTGAGCACCCAGAGCGTGCTGTCCAAGGACCCCAACGAGAAGCGCGATCACATGGTGCTGCTGGAGTTCGTGACCGCCGCCGGCATCACCCTGGGCATGGATGAGCTGTACAAGACTAGTCTCGAGAACGGTGGTTCAGAGCAGAAGCTGATTAGCGAGGAGGACCTGGGCGAGCAGAAGCTGATTTCGGAGGAGGACCTCGGTGAGCAGAAGCTGATTTCCGAAGAGGACCTGAGCGGCTCCGGACACGGTTTCCCCCCAGAGGTGGAGGAGCAGGACGATGGTACCCTGCCCATGTCGTGCGCCCAGGAATCCGGCATGGACCGTCACCCAGCCGCCTGCGCCTCCGCTCGCATTAACGTCGGCTCCGGAACCGGTTCCGGTGCCACTAACTTCAGCCTGTTGAAGCAGGCCGGCGACGTGGAGGAGAATCCAGGACCAATGAAAGCGTTAACGGCCAGGCAACAAGAGGTGTTTGATCTCATCCGTGATCACATCAGCCAGACAGGTATGCCGCCGACGCGTGCGGAAATCGCGCAGCGTTTGGGGTTCCGTTCCCCAAACGCGGCTGAAGAACATCTGAAGGCGCTGGCACGCAAAGGCGTTATTGAAATTGTTTCCGGCGCATCACGCGGGATTCGTCTGTTGCAGGAAGAGGAAGAAGGGTTGCCGCTGGTAGGTCGTGTGGCTGCCGGTGAACCACTTCTGGCGCAACAGCATATTGAAGGTCATTATCAGGTCGATCCTTCCTTATTCAAGCCGAATGCTGATTTCCTGCTGCGCGTCAGCGGGATGTCGATGAAAGATATCGGCATTATGGATGGTGACTTGCTGGCAGTGCATAAAACTCAGGATGTACGTAACGGTCAGGTCGTTGTCGCACGTATTGATGACGAGGTTACCGTTAAGCGCCTGAAAAAACAGGGCAATAAAGTCGAACTGTTGCCAGAAAATAGCGAGTTTAAACCAATTGTCGTAGATCTTCGTCAGCAGAGCTTCACCATTGAAGGGCTGGCGGTTGGGGTTATTCGCAACGGCGACTGGCTGGGATCCCCCGTACAAGATAATGTGAATAAAGATGCCGTCACAGATAGATTGGCTTCAGTGGAGACTGATATGCCTCTAACATTGAGACAGCATAGAATAAGTGCGACATCATCATCGGAAGAGAGTAGTAACAAAGGTCAAAGACAGTTGACTGTATCGATTGACTCGGCAGCTCATCATGATAACTCCACAATTCCGTTGGATTTTATGCCCAGGGATGCTCTTCATGGATTTGATTGGTCTGAAGAGGATGACATGTCGGATGGCTTGCCCTTCCTGAAAACGGACCCCAACAATAATGGGTTCTTTGGCGACGGTTCTCTCTTATGTATTCTTCGATCTATTGGCTTTAAACCGGAAAATTACACGAACTCTAACGTTAACAGGCTCCCGACCATGATTACGGATAGATACACGTTGGCTTCTAGATCCACAACATCCCGTTTACTTCAAAGTTATCTCAATAATTTTCACCCCTACTGCCCTATCGTGCACTCACCGACGCTAATGATGTTGTATAATAACCAGATTGAAATCGCGTCGAAGGATCAATGGCAAATCCTTTTTAACTGCATATTAGCCATTGGAGCCTGGTGTATAGAGGGGGAATCTACTGATATAGATGTTTTTTACTATCAAAATGCTAAATCTCATTTGACGAGCAAGGTCTTCGAGTCAGGTTCCATAATTTTGGTGACAGCCCTACATCTTCTGTCGCGATATACACAGTGGAGGCAGAAAACAAATACTAGCTATAATTTTCACAGCTTTTCCATAAGAATGGCCATATCATTGGGCTTGAATAGGGACCTCCCCTCGTCCTTCAGTGATAGCAGCATTCTGGAACAAAGACGCCGAATTTGGTGGTCTGTCTACTCTTGGGAGATCCAATTGTCCCTGCTTTATGGTCGATCCATCCAGCTTTCTCAGAATACAATCTCCTTCCCTTCTTCTGTCGACGATGTGCAGCGTACCACAACAGGTCCCACCATATATCATGGCATCATTGAAACAGCAAGGCTCTTACAAGTTTTCACAAAAATCTATGAACTAGACAAAACAGTAACTGCAGAAAAAAGTCCTATATGTGCAAAAAAATGCTTGATGATTTGTAATGAGATTGAGGAGGTTTCGAGACAGGCACCAAAGTTTTTACAAATGGATATTTCCACCACCGCTCTAACCAATTTGTTGAAGGAACACCCTTGGCTATCCTTTACAAGATTCGAACTGAAGTGGAAACAGTTGTCTCTTATCATTTATGTATTAAGAGATTTTTTCACTAATTTTACCCAGAAAAAGTCACAACTAGAACAGGATCAAAATGATCATCAAAGTTATGAAGTTAAACGATGCTCCATCATGTTAAGCGATGCAGCACAAAGAACTGTTATGTCTGTAAGTAGCTATATGGACAATCATAATGTCACCCCATATTTTGCCTGGAATTGTTCTTATTACTTGTTCAATGCAGTCCTAGTACCCATAAAGACTCTACTCTCAAACTCAAAATCGAATGCTGAGAATAACGAGACCGCACAATTATTACAACAAATTAACACTGTTCTGATGCTATTAAAAAAACTGGCCACTTTTAAAATCCAGACTTGTGAAAAATACATTCAAGTACTGGAAGAGGTATGTGCGCCGTTTCTGTTATCACAGTGTGCAATCCCATTACCGCATATCAGTTATAACAATAGTAATGGTAGCGCCATTAAAAATATTGTCGGTTCTGCAACTATCGCCCAATACCCTACTCTTCCGGAGGAAAATGTCAACAATATCAGTGTTAAATATGTTTCTCCTGGCTCAGTAGGGCCTTCACCTGTGCCATTGAAATCAGGAGCAAGTTTCAGTGATCTAGTCAAGCTGTTATCTAACCGTCCACCCTCTCGTAACTCTCCAGTGACAATACCAAGAAGCACACCTTCGCATCGCTCAGTCACGCCTTTTCTAGGGCAACAGCAACAGCTGCAATCATTAGTGCCACTGACCCCGTCTGCTTTGTTTGGTGGCGGATCTCCGGCCGACGCCCTGGACGACTTCGACCTGGACATGCTGCCTGCTGATGCTCTCGATGATTTCGATCTGGATATGCTCCCGGGTAACAGCTACCCCTACGATGTTCCCGATTACGCTGGCTACCCCTACGATGTCCCCGATTACGCCGGATCCTACCCCTATGATGTCCCCGACTACGCCTAAGAGCTGTACTAGTTAATAGAATGAATCGTTTTTAAAATAACAAATCAATTGTTTTATAATATTCGTACGATTCTTTGATTATGTAATAAAATGTGATCATTAGGAAGATTACGAAAAATATAAAAAATATGAGTTCTGTGTGTATAACAAATGCTGTAAACGCCACAATTGTGTTTGTTGCAAATAAACCCATGATTATTTGATTAAAATTGTTGTTTTCTTTGTTCATAGACAATAGTGTGTTTTGCCTAAACGTGTACTGCATAAACTCCATGCGAGTGTATAGCGAGCTAGTGGCTAACGCTTGCCCCACCAAAGTAGATTCGTCAAAATCCTCAATTTCATCACCCTCCTCCAAGTTTAACATTTGGCCGTCGGAATTAACTTCTAAAGATGCCACATAATCTAATAAATGAAATAGAGATTCAAACGTGGCGTCATCGTCCGTTTCGACCATTTCCGAAAAGAACTCGGGCATAAACTCTATGATTTCTCTGGACGTGGTGTTGTCGAAACTCTCAAAGTACGCAGTCAGGAACGTGCGCGACATGTCGTCGGGAAACTCGCGCGGAAACATGTTGTTGTAACCGAACGGGTCCCATAGCGCCAAAACCAAATCTGCCAGCGTCAATAGAATGAGCACGATGCCGACAATGGAGCTGGCTTGGATAGCGATTCGAGTTAACGGCCGGCTAGTTGGCCACGTAAGATCCCACCCAGCTTTCTTGTACAAAGTGGTGAGCTCCGCCACCATGGAGCAAAAGCTCATTTCTGAAGAGGACTTGAATGAAATGGAGCAAAAGCTCATTTCTGAAGAGGACTTGAATGAAATGGAGCAAAAGCTCATTTCTGAAGAGGACTTGAATGAAATGGAGCAAAAGCTCATTTCTGAAGAGGACTTGAATGAAATGGAGCAAAAGCTCATTTCTGAAGAGGACTTGAATGAAATGGAGAGCTTGGGCGACCTCACCATGGAGCAAAAGCTCATTTCTGAAGAGGACTTGAATCACCGGTCCACGTGACGTAAGCTAGAGGATCTTTGTGAAGGAACCTTACTTCTGTGGTGTGACATAATTGGACATCGAGGCCTCGAGTTAACGTTACGTTAACGTTAACGTTCGAGGTCGACTCTAGAGGATCCAGATCCACTAGTGGCCTATGCGGCCGCGGATCTGATCCCGGGCGGGTACCGAATTCTAGTATGTATGTAAGTTAATAAAACCCTTTTTTGGAGAATGTAGATTTAAAAAAACATATTTTTTTTTTATTTTTTACTGCACTGGATATCATTGAACTTATCTGATCAGTTTTAAATTTACTTCGATCCAAGGGTATTTGAAGTACCAGGTTCTTTCGATTACCTCTCACTCAAAATGACATTCCACTCAAAGTCAGCGCTGTTTGCCTCCTTCTCTGTCCACAGAAATATCGCCGTCTCTTTCGCCGCTGCGTCCGCTATCTCTTTCGCCACCGTTTGTAGCGTTACCTAGCGTCAATGTCCGCCTTCAGTTGCACTTTGTCAGCGGTTTCGTGACGAAGCTCCAAGCGGTTTACGCCATCAATTAAACACAAAGTGCTGTGCCAAAACTCCTCTCGCTTCTTATTTTTGTTTGTTTTTTGAGTGATTGGGGTGGTGATTGGTTTTGGGTGGGTAAGCAGGGGAAAGTGTGAAAAATCCCGGCAATGGGCCAAGAGGATCAGGAGCTATTAATTCGCGGAGGCAGCAAACACCCATCTGCCGAGCATCTGAACAATGTGAGTAGTACATGTGCATACATCTTAAGTTCACTTGATCTATAGGAACTGCGATTGCAACATCAAATTGTCTGCGGCGTGAGAACTGCGACCCACAAAAATCCCAAACCGCAATCGCACAAACAAATAGTGACACGAAACAGATTATTCTGGTAGCTGTGCTCGCTATATAAGACAATTTTTAAGATCATATCATGATCAAGACATCTAAAGGCATTCATTTTCGACTACATTCTTTTTTACAAAAAATATAACAACCAGATATTTTAAGCTGATCCTAGATGCACAAAAAATAAATAAAAGTATAAACCTACTTCGTAGGATACTTCGTTTTGTTCGGGGTTAGATGAGCATAACGCTTGTAGTTGATATTTGAGATCCCCTATCATTGCAGGGTGACAGCGGACGCTTCGCAGAGCTGCATTAACCAGGGCTTCGGGCAGGCCAAAAACTACGGCACGCTCCTGCCACCCAGTCCGCCGGAGGACTCCGGTTCAGGGAGCGGCCAACTAGCCGAGAACCTCACCTATGCCTGGCACAATATGGACATCTTTGGGGCGGTCAATCAGCCGGGCTCCGGATGGCGGCAGCTGGTCAACCGGACACGCGGACTATTCTGCAACGAGCGACACATACCGGCGCCCAGGAAACATTTGCTCAAGAACGGTGAGTTTCTATTCGCAGTCGGCTGATCTGTGTGAAATCTTAATAAAGGGTCCAATTACCAATTTGAAACTCAGTTTGCGGCGTGGCCTATCCGGGCGAACTTTTGGCCGTGATGGGCAGTTCCGGTGCCGGAAAGACGACCCTGCTGAATGCCCTTGCCTTTCGATCGCCGCAGGGCATCCAAGTATCGCCATCCGGGATGCGACTGCTCAATGGCCAACCTGTGGACGCCAAGGAGATGCAGGCCAGGTGCGCCTATGTCCAGCAGGATGACCTCTTTATCGGCTCCCTAACGGCCAGGGAACACCTGATTTTCCAGGCCATGGTGCGGATGCCACGACATCTGACCTATCGGCAGCGAGTGGCCCGCGTGGATCAGGTGATCCAGGAGCTTTCGCTCAGCAAATGTCAGCACACGATCATCGGTGTGCCCGGCAGGGTGAAAGGTCTGTCCGGCGGAGAAAGGAAGCGTCTGGCATTCGCCTCCGAGGCACTAACCGATCCGCCGCTTCTGATCTGCGATGAGCCCACCTCCGGACTGGACTCATTTACCGCCCACAGCGTCGTCCAGGTGCTGAAGAAGCTGTCGCAGAAGGGCAAGACCGTCATCCTGACCATTCATCAGCCGTCTTCCGAGCTGTTTGAGCTCTTTGACAAGATCCTTCTGATGGCCGAGGGCAGGGTAGCTTTCTTGGGCACTCCCAGCGAAGCCGTCGACTTCTTTTCCTAGTGAGTTCGATGTGTTTATTAAGGGTATCTAGCATTACATTACATCTCAACTCCTATCCAGCGTGGGTGCCCAGTGTCCTACCAACTACAATCCGGCGGACTTTTACGTACAGGTGTTGGCCGTTGTGCCCGGACGGGAGATCGAGTCCCGTGATCGGATCGCCAAGATATGGCGACAATTTTGCTATTAGCAAAGTAGCCCGGGATATGGAGCAGTTGTTGGCCACCAAAAATTTGGAGAAGCCACTGGAGCAGCCGGAGAATGGGTACACCTACAAGGCCACCTGGTTCATGCAGTTCCGGGCGGTCCTGTGGCGATCCTGGCTGTCGGTGCTCAAGGAACCACTCCTCGTAAAAGTGCGACTTATTCAGACAACGGTGAGTGGTTCCAGTGGAAACAAATGATATAACGCTTACAATTCTTGGAAACAAATTCGCTAGATTTTAGTTAGAATTGCCTGATTCCACACCCTTCTTAGTTTTTTTCAATGAGATGTATAGTTTATAGTTTTGCAGAAAATAAATAAATTTCATTTAACTCGCGAACATGTTGAAGATATGAATATTAATGAGATGCGAGTAACATTTTAATTTGCAGATGGTTGCCATCTTGATTGGCCTCATCTTTTTGGGCCAACAACTCACGCAAGTGGGCGTGATGAATATCAACGGAGCCATCTTCCTCTTCCTGACCAACATGACCTTTCAAAACGTCTTTGCCACGATAAATGTAAGTCTTGTTTAGAATACATTTGCATATTAATAATTTACTAACTTTCTAATGAATCGATTCGATTTAGGTGTTCACCTCAGAGCTGCCAGTTTTTATGAGGGAGGCCCGAAGTCGACTTTATCGCTGTGACACATACTTTCTGGGCAAAACGATTGCCGAATTACCGCTTTTTCTCACAGTGCCACTGGTCTTCACGGCGATTGCCTATCCGATGATCGGACTGCGGGCCGGAGTGCTGCACTTCTTCAACTGCCTGGCGCTGGTCACTCTGGTGGCCAATGTGTCAACGTCCTTCGGATATCTAATATCCTGCGCCAGCTCCTCGACCTCGATGGCGCTGTCTGTGGGTCCGCCGGTTATCATACCATTCCTGCTCTTTGGCGGCTTCTTCTTGAACTCGGGCTCGGTGCCAGTATACCTCAAATGGTTGTCGTACCTCTCATGGTTCCGTTACGCCAACGAGGGTCTGCTGATTAACCAATGGGCGGACGTGGAGCCGGGCGAAATTAGCTGCACATCGTCGAACACCACGTGCCCCAGTTCGGGCAAGGTCATCCTGGAGACGCTTAACTTCTCCGCCGCCGATCTGCCGCTGGACTACGTGGGTCTGGCCATTCTCATCGTGAGCTTCCGGGTGCTCGCATATCTGGCTCTAAGACTTCGGGCCCGACGCAAGGAGTAGCCGACATATATCCGAAATAACTGCTTGTTTTTTTTTTTACCATTATTACCATCGTGTTTACTGTTTATTGCCCCCTCAAAAAGCTAATGTAATTATATTTGTGCCAATAAAAACAAGATATGACCTATAGAATACAAGTATTTCCCCTTCGAACATCCCCACAAGTAGACTTTGGATTTGTCTTCTAACCAAAAGACTTACACACCTGCATACCTTACATCAAAAACTCGTTTATCGCTACATAAAACACCGGGATATATTTTTTATATACATACTTTTCAAATCGCGCGCCCTCTTCATAATTCACCTCCACCACACCACGTTTCGTAGTTGCTCTTTCGCTGTCTCCCACCCGCTCTCCGCAACACATTCACCTTTTGTTCGACGACCTTGGAGCGACTGTCGTTAGTTCCGCGCGATTCGGTTCGCTCAAATGGTTCCGAGTGGTTCATTTCGTCTCAATAGAAATTAGTAATAAATATTTGTATGTACAATTTATTTGCTCCAATATATTTGTATATATTTCCCTCACAGCTATATTTATTCTAATTTAATATTATGACTTTTTAAGGTAATTTTTTGTGACCTGTTCGGAGTGATTAGCGTTACAATTTGAACTGAAAGTGACATCCAGTGTTTGTTCCTTGTGTAGATGCATCTCAAAAAAATGGTGGGCATAATAGTGTTGTTTATATATATCAAAAATAACAACTATAATAATAAGAATACATTTAATTTAGAAAATGCTTGGATTTCACTGGAACTAGAATTAATTCGGCTGCTGCTCTAAACGACGCATTTCGTACTCCAAAGTACGAATTTTTTCCCTCAAGCTCTTATTTTCATTAAACAATGAACAGGACCTAACGCACAGTCACGTTATTGTTTACATAAATGATTTTTTTTACTATTCAAACTTACTCTGTTTGTGTACTCCCACTGGTATAGCCTTCTTTTATCTTTTCTGGTTCAGGCTCTATCACTTTACTAGGTACGGCATCTGCGTTGAGTCGCCTCCTTTTAAATGTCTGACCTTTTGCAGGTGCAGCCTTCCACTGCGAATCTTTAAAGTGGGTATCACAAATTTGGGAGTTTTCACCAAGGCTGCACCCAAGGCTCTGCTCCCACAATTTTCTCTTAATAGCACACTTCGGCACGTGAATTAATTTTACTCCAGTCACAGCTTTGCAGCAAAATTTGCAATATTTCATTTTTTTTTATTCCACGTAAGGGTTAATGTTTTCAAAAAAAAATTCGTCCGCACACAACCTTTCCTCTCAACAAGCAAACGTGCACTGAATTTAAGTGTATACTTCGGTAAGCTTCGGCTATCGACGGGACCACCTTATGTTATTTCATCATGGGCCAGACCCACGTAGTCCAGCGGCAGATCGGCGGCGGAGAAGTTAAGCGTCTCCAGGATGACCTTGCCCGAACTGGGGCACGTGGTGTTCGACGATGTGCAGCTAATTTCGCCCGGCTCCACGTCCGCCCATTGGTTAATCAGCAGACCCTCGTTGGCGTAACGGAACCATGAGAGGTACGACAACCATTTGAGGTATACTGGCACCGAGCCCGAGTTCAAGAAGAAGGCGTTTTTCCATAGGCTCCGCCCCCCTGACGAGCATCACAAAAATCGACGCTCAAGTCAGAGGTGGCGAAACCCGACAGGACTATAAAGATACCAGGCGTTTCCCCCTGGAAGCTCCCTCGTGCGCTCTCCTGTTCCGACCCTGCCGCTTACCGGATACCTGTCCGCCTTTCTCCCTTCGGGAAGCGTGGCGCTTTCTCAATGCTCACGCTGTAGGTATCTCAGTTCGGTGTAGGTCGTTCGCTCCAAGCTGGGCTGTGTGCACGAACCCCCCGTTCAGCCCGACCGCTGCGCCTTATCCGGTAACTATCGTCTTGAGTCCAACCCGGTAAGACACGACTTATCGCCACTGGCAGCAGCCACTGGTAACAGGATTAGCAGAGCGAGGTATGTAGGCGGTGCTACAGAGTTCTTGAAGTGGTGGCCTAACTACGGCTACACTAGAAGGACAGTATTTGGTATCTGCGCTCTGCTGAAGCCAGTTACCTTCGGAAAAAGAGTTGGTAGCTCTTGATCCGGCAAACAAACCACCGCTGGTAGCGGTGGTTTTTTTGTTTGCAAGCAGCAGATTACGCGCAGAAAAAAAGGATCTCAAGAAGATCCTTTGATCTTTTCTACGGGGTCTGACGCTCAGTGGAACGAAAACTCACGTTAAGGGATTTTGGTCATGAGATTATCAAAAAGGATCTTCACCTAGATCCTTTTAAATTAAAAATGAAGTTTTAAATCAATCTAAAGTATATATGAGTAAACTTGGTCTGACAGTTACCAATGCTTAATCAGTGAGGCACCTATCTCAGCGATCTGTCTATTTCGTTCATCCATAGTTGCCTGACTCCCCGTCGTGTAGATAACTACGATACGGGAGGGCTTACCATCTGGCCCCAGTGCTGCAATGATACCGCGAGACCCACGCTCACCGGCTCCAGATTTATCAGCAATAAACCAGCCAGCCGGAAGGGCCGAGCGCAGAAGTGGTCCTGCAACTTTATCCGCCTCCATCCAGTCTATTAATTGTTGCCGGGAAGCTAGAGTAAGTAGTTCGCCAGTTAATAGTTTGCGCAACGTTGTTGCCATTGCTACAGGCATCGTGGTGTCACGCTCGTCGTTTGGTATGGCTTCATTCAGCTCCGGTTCCCAACGATCAAGGCGAGTTACATGATCCCCCATGTTGTGCAAAAAAGCGGTTAGCTCCTTCGGTCCTCCGATCGTTGTCAGAAGTAAGTTGGCCGCAGTGTTATCACTCATGGTTATGGCAGCACTGCATAATTCTCTTACTGTCATGCCATCCGTAAGATGCTTTTCTGTGACTGGTGAGTACTCAACCAAGTCATTCTGAGAATAGTGTATGCGGCGACCGAGTTGCTCTTGCCCGGCGTCAATACGGGATAATACCGCGCCACATAGCAGAACTTTAAAAGTGCTCATCATTGGAAAACGTTCTTCGGGGCGAAAACTCTCAAGGATCTTACCGCTGTTGAGATCCAGTTCGATGTAACCCACTCGTGCACCCAACTGATCTTCAGCATCTTTTACTTTCACCAGCGTTTCTGGGTGAGCAAAAACAGGAAGGCAAAATGCCGCAAAAAAGGGAATAAGGGCGACACGGAAATGTTGAATACTCATACTCTTCCTTTTTCAATATTATTGAAGCATTTATCAGGGTTATTGTCTCATGAGCGGATACATATTTGAATGTATTTAGAAAAATAAACAAATAGGGGTTCCGCGCACATTTCCCCGAAAAGTGCCACCTGACGTCTAAGAAACCATTATTATCATGACATTAACCTATAAAAATAGGCGTATCACGAGGCCCTTTCGTCTCGCGCGTTTCGGTGATGACGGTGAAAACCTCTGACACATGCAGCTCCCGGAGACGGTCACAGCTTGTCTGTAAGCGGATGCCGGGAGCAGACAAGCCCGTCAGGGCGCGTCAGCGGGTGTTGGCGGGTGTCGGGGCTGGCTTAACTATGCGGCATCAGAGCAGATTGTACTGAGAGTGCACCATATGCGGTGTGAAATACCGCACCGAATCGCGCGGAACTAACGACAGTCGCTCCAAGGTCGTCGAACAAAAGGTGAATGTGTTGCGGAGAGCGGGTGGGAGACAGCGAAAGAGCAACTACGAAACGTGGTGTGGTGGAGGTGAATTATGAAGAGGGCGCGCGATTTGAAAAGTATGTATATAAAAAATATATCCCGGTGTTTTATGTAGCGATAAACGAGTTTTTGATGTAAGGTATGCAGGTGTGTAAGTCTTTTGGTTAGAAGACAAATCCAAAGTCTACTTGTGGGGATGTTCGAAGGGGAAATACTTGTATTCTATAGGTCATATCTTGTTTTTATTGGCACAAATATAATTACATTAGCTTTTTGAGGGGGCAATAAACAGTAAACACGATGGTAATAATGGTAAAAAAAAAAACAAGCAGTTATTTCGGATATATGTCGGCTACTCCTTGCGTCGGGCCCGAAGTCTTAGAGCCAGATATGCGAGCACCCGGAAGCTCACGATGAGAATGGCCAGACCATGATGAAATAACATAAGGTGGTCCCGTCGGCAAGAGACATCCACTTAACGTATGCTTGCAATAAGTGCGAGTGAAAGGAATAGTATTCTGAGTGTCGTATTGAGTCTGAGTGAGACAGCGATATGATTGTTGATTAACCCTTAGCATGTCCGTGGGGTTTGAATTAACTCATAATATTAATTAGACGAAATTATTTTTAAAGTTTTATTTTTAATAATTTGCGAGTACGCA
