# Supplementary material for: In vivo study of gene expression with an enhanced dual-color fluorescent transcriptional timer
Source: eLife. 2019 May 29;8:e46181. doi: 10.7554/eLife.46181 (PMC6660218; doi:10.7554/eLife.46181)
Supplement: Supplementary file 5. [file elife-46181-supp5.docx]

**Supplementary File 5.** **Mapping Results of enhancer trap lines by splinkerette PCR.**

**Inx2 5’ site sequence:**

"ACNTTNGGGNNCGNTTTCCTCATACCGCGCACTCTGCACCAACTTTCAACTCGGACTCTGCCATATTGGAGCTGCATTTGCGTTGGTATTTGTGTGTGTGTAGCAAGTTCTTGGAACCCGTTGTGTTCTTACTTGTGTACATGTGGCTGCTGGAGTGGATTTCAATTTCGAGATGGGAGCTGCTGCACCACCGACCAAAAAGAACAAGCAAAGCCAGTCAAAAAGCGATGAGTAAAAGAGAGAACACGAAGTAGATGGAGAGGTGGGAAAACATACTGGAACTCTCTGCTTTCACTGCATTTCTATACCTGTGGACCCCTTGTGTACCTGCCAACTTCCAACAATCGCCGGCGTACGCAAATCGAATATACCTGCATTCCAGGTATAGCAAAATAATATTCATCGGGATTTTTCATGCCCTTTTTCGTGGAGAGAACAGAGATCCCACTAGTGTCGACACCAGTCTCNTTTCAGCCACAAN"

**Inx2 3’ site sequence:**

"AACTTCGGTAGCTTCGGCTATCGACGGGACCACCTTATGTTATTTCATCATGAACGGGGGGTTAAATGCATCGGCAGACTCGTGAGCGTCGGCGTTGCCAGATGACATTAAAACTAAATCATAAATCTTGGGTGTGCCAAACAGGCTTAACCCCAGAAGGTATGCAACACTGTGTGGTAGGTTTAAAAATTCCAAAATAATTTGAAATTGCCATAAATAAAATTAAAGTAAAATAGGTTTCGGAAAGCCAAAAAAGAGGGAAATTGATTTAAAATGAAGTCAAGTTAGTTCTTGGTTTGTTTCTTTTTATCTTTAAAAATCGTGCATGGCCGTAATGAAACTAATCTAGGGTTAATGACGGATAAGGCTGCCAGATGTTCGATGAAAACGCAGTTGGTTCACTTTAGGCGTAAAATGCAACGGGATACGAGGCATGTTTTAAGCATATTCAAAATTAAGGTTAATAATTAAGGCGGTCTTTTTTGTTTTAATTCAGTTTTGAGCAGCATCACTGAGTCACTCTCGCCCCCTCTGCTTCACTCCGGGATTGGGAAATGTTACCCTTTTGGTCCATGCCAGTTCAGTCCAGTCCAAATTCAGCCATTCCAAGTCGCGAGAGCCGGGAGAGGATTGAAAAACGAAACAAAAATAAATGTAAAGTAAGCACTCCATTGTTAAACAAGATCCCACTAGTGTCGACACCAGTCCTTTTCCANCCACA"

**Lk6 5’ site sequence:**

"GGTNNCCGGGNGAGTNNCCGTGTTCNCCNGATTCATTCGNACTTTGAGATGGATCGGTAGCTGGGAAGGAAACTGTNAAGCGAAGTACGCGCAGAANGACGGCTTTTGTCCGCTATTCAGCGATTTTTTTTGTGTTGTNATCAGCAGAGGAAATTTTAACGANCAACTCCGNCGCNACACCAGNCATCTCCAGCNGCCCCGGANNATGNAGTAGAACTAANTNAACGCCNCGATCACTACAACNGACCATCTCACCAACNACTACTNGAGCAACAACCNCAGNNNCNGCNCTACTGCNCCNAGCCCNCNGAGAAGAGGTGAAACGNAATAATCGGCAATACCCGAAGAANANNNCAAAANNGTATCGCANATAACCGAAAAAAGCGGTGCNATANATNNNCCCNATTTTTTGCTTGAGCTTTTTTCGCCTGTGTGANGAGAGAAATCNNNNGCAGCCNTCNNTTACAACANCAACNGCAGCCNCNCCNNCGACGACTCNACCACCAAAGCGAAGAATAAATAANCTGCGGANNGCGATGGATNGNGAGAGAGAGAGAGAGGAGCATCNGCANCAGANTTGANTCAGTTTTTTCATCGNCCGCCGCNTTGTATTAAAANCNANAGGAGANNANNNACGANTANCACACNNNTCCCATAGGCGGCCACCATTTTGATTTTGAANGANCTGNNCGAGANGAGAGGANNGAGCNCGAGAGACCGAGAAAAACTGCTGCTCATTTGTTGCACCGTAAACCCTTTTTCCNGCTNGNNNGCACTCTGCCTGTNTCACTCTANTCGCGTNCAGGAGCNCCGTGACAGTNACNACCTGCAGNCACTGNNTGNTGGAGCCCAANTCNGGGACAGCTGCTTCTGCCGCTGCTGCNANAGCNAGCTNCNTCATTGACAGNANCCNCCCCCGTGGATCCCACTAGTGTCGACTCAGNANNTTTNCCCCCCCAAANTTTTTGGCCCCTNCNATNGGNGCCTGNGTNGNCNCGNCGGNTGNCCATTTANGCNATTTCNGNTGGCGTTCNCTTTGNTTGGTTTCCNCCTCNTGCCTTTNGCCGGGTTTGCCATTGAGANTGANACTCNACGNGCGCGNTCTTNGCATGNTGCGGT"

**Lk6 3’ site sequence:**

"AGCCCGGGAGAATTCGTCGTCATTCGTCGGAATCATTCGAACTTTGAAAATGGATCGGTAGCTGGGAAGGAAACTTAAAGCGAAATACGCAAAGAAAACGGCTTTTGTCCGCTATTCAGCGATTTTTTTTGTGTTGTAATCAGCAGAGGAAATTTTAACGACCAACTCCACCGCCACACCAGCCATCTCCAGCAGCCCCGGAAAATAAAATAGAACTAAATTAACGCCACCATCACTACAACAACCATCTCACCAACAACTACAAGAGCAACAACCACAGCAACAGCACTACTGCACCAAGCCCACAAAGAAGAGGTGAAACGCAATAATCGCAATACCCGAAGAAAAAAACAAAAAAATATCGCAGATAACCGAAAAAAGCGGTGCAATAGATAAACCCCATTTTTTGCTTGAGCTTTTTTCGCCTGTGTGATGAGAGAAATCAGCAGCAGCCATCGATTACAACAACAACAGCAGCCACACCAACGACGACTCAACCACCAAACGAAGAATAAATAAACAGCGGAGAGCGATAGATAGAGAGAGAGAGAGAGAGGAGCATCTGCAGCAGAATTGAATCAGTTTTTTCATCAACCGCCGCATTGTATTAAAAACTAGAGGAGAACAAAAACGAATAACACAAAAATCCCATAGGCGGCCACCATTTTGATTTTGAATGAACTGAACGAGAAGAGAGGAAAGAGCACGAGAGACCGAGAAAAACTGCTGCTCATTTGTTGCACCGTAAACCCTTTTTCCAGCTTGTAAGCACTCTGCCTGTCTCACTCTAATCGCGTCCAGGAGCACCGTACAGTAACAACAACAGCAACAACATGGTGGAGCCCAAGTCCGGGACAGCTGCTTCTGCCGCTGCTGCGAAAGCAAGCAACAACAATAACAACAACCACCCCCGTGGATCCCACTAGTGTCGACACNGTNCTTTTTTTNCNCCCCAAANNN"

**Schizo 5’ sequence:**

"CTTCGGTAGCTCGGCTATCGACGGGACCACCTTATGTTATTTCATCATGGTTCGACGTTTGGAAACAGTGTGGACGTGCTGGGGGGCACCGCAGTGTGACCATAGAGCATGCAACTCAAAATACGCTATCGTATTCATAAAATATACCGCATCAAATGTGGCATTTTAAGACGACCGTTTTATTTTAAAATATATTGTTGTTTTCATCTTATTTAAGAAGGGCATAAACAATCTTAAGTTTATGCTCATTATTATACATAAGCATTTTCTTTATTTGATAAATTTAAAACTCATTAAATCTAAATATGTGTGCATTAATTAAGATTTATTTTGGAACTGCCAATGATCCATTCCTGGTTGATCTAGTTCACCACAAAAATANTCTATAAAATTGGTATTGTAATAATAAGCCGACATGAAGAACCAAAAAAATATAACCATTATACTAACTGGGCCACCAGGTGCAAATTTAGCTGGGAAAATATTTGCTATTTGTTTATTATGTATGTAATCATGATTTTCAGGAGTGGGTAAAACTACACTGGTCCACAAAATATGCTCGGCTCTGCAGGATAGAGGACGCATTCTTCAAGGATTCTACACAGAGGAAATGCGAGGTGAGGGCACCAGCCAAAGAATCGGCTTCGACGTGGTCACGTTAGCCGGAAAACGGGCAATCCTATCTCGCAAGAATCCCGGGGACCAGCTGCGACGACCCAAAGTGGGAGAGTATTCGGTGTTTGTGCAGGACTTCGACAGTCTCGCTCTGCCAGTACTTGGCACACAGGATTCCCAACCAGAGCCGGATCCCACTAGTGTCGACACCAGTCCNTTCCNNCCCCCAA"

**Schizo 3’ sequence:**

"GGNTTTTTCGCTCGCGCGNTGAATTGGAAAATCCGNTAAATTCCGCAGAAATATTCGNTAATCCCCCTCCGTATCCGCACCANGTGTTNGCCTCGCGACATCNNAGGTGCATGAGCTCTGCATTTTGAGGCAGAGTATAATATACGAAGGTCATTCAATTACAATACGAGTGAAGTTTCAACTCGNAGCAAGCTGCATTTTCAGTTCTTATTGTGAGATTCAAAGTGGTTCTGCCTGTTTCGCTGTTTTATCACCCTCTCCACCACCTTCCCTCATTCGCGCGAGTGCGTGTGCGTGGCGGGGCTATAATGTTTTGGCCACAAAAGCACATCGATTAGGAGTGCAGCCGATCCCCNNTNNGGNNANANNCNNANCATGNCCNAGNACGATCTGANNAATCTATCCC"

**Qsm 3’ site sequence:**

"AACCTTNGGTAGCTTCGGCTATCGACGGGACCACCTTATGGTTATTTCATCATGGCTTGGTANGATTTTGCTCCCACAATTTCTCAGATTTTTGTCATGGGACAAAAAAAAAATTATACAAACTTACCGGCTGATTGTTATCTCCGGCAGAGAGCGGAAAAGAGCGGTAGTGAGAGCGATCCTTTTACCGTATTTTACAGCCTCTCGGTTAAGCTAACTTGCTAAAGTCGCTCTCTTTTTCGCCGTGCGACAGAGATGCTCCGTGCGAGAGAGCGACTTACAGTGGGTCATTTTAATTGTGCTCTGCTTATCTAAATGTATTTAAGTATATCCATAAATTGTAATGCAGCACTTTCGTAATTGCCGGCTTTATTTATGCACTGTGAACAGCCAGGGGCTAGATGGCGCCTTAGCATTTTTCCCCACTGTCCACGCTCTAATGCCTAAGAGCGTTGGTTCACTAGCTAGTTTATAGCTAACTTTTGAAAACTCCGTTTGTTAAATAATTATTCAACACTATCTTATTCAAAATTCTTAAAATATTTTCTAAGGATTTTTTTTTTTACCAATGAAAGCATAATATAATAAAAATGCATATAGATTTATGTTGCAATAATTCGGATAAAACGGCACACTTATCTTCTTATATACATACGTACTTATTACATTTCTTTTGGGTAATTAACCAATTGGTTAACATTGAGTGCTTTCACTATAGTTTTGCACTCTCTGCCATATAAGAACGACTTCTGCTCTGAAACTGAGTCAGCATCTCTATACGCCGGCTCTCGATTCCCGAGGTAATGAAGTTGCAGCCGAGCTGAGCCTCAGTTGCCTGGCGACTAGCGAACAGCTTGGACGTGTGGTGTGCTCTTCGGTGTGGNTTCGAGTCTCGAGTTCCGAGTTTTCCCCATATTGCAGCCGTCANGTAAGACCCGCTCCGAGTTTTGTGNGANTTGNGNGANNGNGNGNGGTTCGCGGCTCGTTCGTTTTTATTTGCNGTTGNCATNGGTTTTGATTGTTGAGCGGNCCCNCANGCCATTGAGGGATTCNCTTCNGGCTTGGTTCNAAAACCCNCTGGNGGGTTGCAAGGCGGNCANAAAAGGTTANGGACNACAAANGAATGGGNNTGGGAAACNCCAAAAGNCNGCCANAANAAAAAAAAAAAAAAGTGGCCCCTGGGANAAGGCCNTTCTCGCTCGTCNTCAANGCTGNAAAAAGCCTTGGCTNCNTTCNCCCANATNAACCCAGGCACNNNNTNTTNNCCNAA"

**Tsp42Ea 5’ site sequence:**

"AAGANTNTNCTTCGGTAGCTTCNGCTATCGACGGGACCACCTTATGTTATTTCATCATGGGTTGAGGCTGCTTGGCGCAGCTTTTGACCGATCAGNTTANTTCAGTTAGCATTGTCAACTGCTCACGAACNGTTCGAAAAGCGGANCNAGCGTAAAATCATTCTGTAAATCATTCAAAAGGCGGAAAACTCAAGGTGTGTGTGTGGGTTTTGTTTTCAAATTCCGTGTGCTTGTGTGTGTGAGAGAGGGCGAGAGCGACAGTGTCGTAGCAGGCAGTGTTGTAAGGTGCCGAAACAGCGATCCCACTAGTGTCGACACCAGTCTCATTCAGCCACAANTGANGCAAANNTCNTTNTCNNGGTNGTACNNANCCNGAATTGCNAACNNNCACANTTTGACTNCNTCNTCNCANCANCTCATGATTCNTNNATNNCCNCATNGACNNNTNACATNCTCANNTCCNNGTNGCNACCNTGTGNNTCCNATGANNANANNCTCATC"

**Tsp42Ea 3’ site sequence:**

"NGCGCAACTTCACACGAAGCATAATTCGGTTTTGATTTTGGTTTGATTTCGTCACCCCCTCCCAACGGATAATTACTGATAACCTTGGTTNTTTNCGCCTCNTCTGTTACCAAGTCNCGATTNAGTGTTATATTANGCAANTNTGANATGNTCACGGGAATTACGTACANATTTGTTTAACTTTTACTAACAATAAATGTTTTATTTATGCAAATGCACTANTGCCTGGCCAATAGGAGAATTAACAANAGAAAAATCGG"

**Sd 5’ site sequence:**

"TCTTCGGTAGCTTCGGCTATCGACGGGACCACCTTATGTTATTTCATCATGGTTTATGCCATCGCTTGCGTCTGTCTGTGTTATTTCGATTGCGAGAGCTAGTTAACATTAGTTAAAATTAAAACAAACGCCAGCCATAAGATATAATCGAATTGCGGTTTTTTCTTCTCGTACCGAATTTCCATCCACATCCGCATCCACCATCCGCCATCCGCCAAACCCATCCCGTTCCGTTTGTTCGTCGCTATCCGTCGGATATGTTGTTCTATCGGTGTACCGTACAAGTACAAGTTCGTACCCAGTCCAAAGCCAAACAGATTGCGCCGTGTGCCGGTAAAAATAAGCGCAAAGTTTGAGAAAAACCATCAACATCAGATGATGTGCCCTGGAGGAAAAAACGTCGCGGATTTAATGAAATCTTAAATAATGGGTTCGCTTTAGCACAATTCGACGTTGCATTCGGCCAGCGGTGTGTTGTGTAAACAAAGTTGTAGTTATAGTTTTAGTTTAATTAGCTTCGCATAAGCTGCAACCGGATCCCACTAGTGTCGACACCAGTCTCTTTCCAGCCACAAN"

**Sd 3’ site sequence:**

"GNCACGACTCCACTAAGAATTATCTCANAATGTATTTCAGGNAAAAAGTAAAACAGTAAAAGCTCTTCTCATTGAGCATTCGCATTCGCACTCGGCTGCTTCCATCGAAAAATTTTTCGAAACTGAAAGCAGAAATATGTTTTTCTGGCCATTCNTATTCNACACGAAACTCTCTCGCATCTCTTCCTCGCTCTCTTTGTGCGCGGTTCCAATCGGAACCAATTGTGGAACCTAAGAGAGCGTTAATTCTAAGATTTTACATAAAGAGNATCCGTTTTTCTCTTTNATTTTCATACATTTTTATGTTTTCTCTTCCCCTAAATTATGTTAAAAGCTTTAATACCAAAGCTTCACTTTATTTGACAGCTGATTCAGCTGAAGGTGATTGCCGCCCTTTTTTGAACTCCACTAAAAAATGCCTATTTTGGTATATTTGATTAGGTTATCTGTTGATTACAATATATTTAAGCTGTTTGTCCAATTAGCGAAGATATTTAAAAAGTTTATTTCACTTTTTGATATGCTTTTAATTTAACTTTTAAATTAGACTGTGTTCCTGTTCGGAATACGGTCACACCGGAACAAAAACATAAACAGACCTTTGAATGCATTCCACTGTGCAGTCAAAAGTAATAAATATAAGCTCCTCAGTTTTATACGTCAAACTATCGCTTAAGCAATGTATAAAATTAGTTTATAAGTTGGTTACATTTTAGTTTATTCTCTTTTAATGGCTCTAAGTGACTATTAAATCCGTGTTGTGGTAGCTCTTTGGTTGGCTTGAATAGCCAGCAGTAACTAANTATAAAAATAAAGAANGTAATTGAGAGACTTGGAGGGAAGANCAGCGCATACGCGAGAGTGGTCGAGTGAATGNTTACNGCGCATGAATTAGGTTTTGGNGGGTTTATCTGANTACCCTCTAGCCCCATGACTAANGCTTAAAGATGGGGCTGACCTNGGGGNGTAATCCCCTGCCTCTACNGGTTNNTTATCCAACNCGGGGCANNGTTGGANTTAACTGGCTTAGCNTTNAAAGCCCAATNNAGGGCAANAGGNGTGGGAAAAGCTNCNTNTTTNCCNGCNCNGGGGGGCATTGGACCNCCCNNNCCNATTNGAN"

**DopEcR 3’ site sequence:**

"CCGNCGCCGNGCCGANCTCANAACNCACATTAGGGNAAAATTTAGCACAAAANAACGAAGCATTTCAGTTGGAAAGCTCAATGCAAAGAATGGCAGCATTCGCTTGGGGATTAGCCAAAGCCAAAAGACTTTGCGCAAATACTGAAAAGCTCGGCCTGAGCGCATCATTTTCCACGCAAATTTCGTTTGCGAAAATTTCAATTTATGTGTGGCAAACAGGATTTGCAGTATATGTCTGCATATGTATGTATGTATCTGTAACTCGGTCACACGTTACACGCTTCTCCGTTTTTTTTTTTTCCCCACCATTTTATTGACTTTGTCGTTTTATAGGCAATTTGNCTTCNGGCANCCGTCGATAGCTTTTGCTGNGTCATAAAATTACNCGCCAAGCGAANGCCCCGNATCCTTATNGGTATCCTTATCCTTATCGGCAAGGACCGGGTTTCGAGCTNGGACTCTTTCNCTGCCAAGTTGTTTATATTGNCGNAAATTTGCCTTCAAGGACGCGACAACACCGCACANACNGCCGCCTCCCACCGCCCCCTACCCGCTCACATGGCCATTTTATCGATCACACAANGGGAAATTTCACACGTTGTCCCAAAGGGCTGGNAAATGGGTTGGGAAAGGTACACNGGTCNCNCCNGGCTTTAATTAATTCGAATCACTAGGATATGGATAGACCCACTGCAACAAATTGGGGATATAATTACANAGAACTGTATCTCCGCGTATAGATCATATAACGTATTAGAAATACATAAGTAAAAAATTACCATTCAATCTATATTTTANATCCCACTAGTGTCGACACCAGTCCATTNCNNC"

**CrebA 5’ site sequence:**

"GGNGGGTCTTCGGGTAGCTTCGGCTATCGACGGGACCACCTTATGTTATTTCATCATGGTTCGGTTCGCATTCACAAGAATTTCACCGACATTTAGTTTAGTACCGTTTTTGGCTGCAGTCGCACTACGGACAGTTTATTCTAATTGTGTGCTACCATCCCATACATCCAAACATACAAATATTATTGCTAAAGTTAACGATATTAACTACACATATTAAATGCTATTATACACGGGCCGAGCAAAGCCATTTCTAAAGCGGTTTACTGCCCATTCACACACAAAACACATCAAAAAATTCAATTAAATCAATTGAAATCGTAATCGAGTCCAAAACACAGTACAGCGAAAAGAAAAATCAAATAAACTAAAATTATATGGTTATCAACGTTGAATCTAAACATACATATAATAACTATTTTCTATGAAAATCGAGTGTTTCGTTTCCGAATCGGGAAAAAAGTTAATAATTCAATTAATGTTAACGCCAAATATAATCCGACGAGAAAAGCGCATGCAACAAGTTAAATCCAGCCGACAGTTGTAAACCAAGTTAGATCCCACTAGTGTCGACACCAGTCTCATTCAGCCACAA"

**CrebA 3’ site sequence:**

"GTCGATCCTCCNACAGANGATCGGCCGGCCGANCGNCTCAGATCGCCATCATCTTTTCCGCATACAACGCCTTTGCTCGTCNTCCTTCCGCACGATAATAGCGCATCTCTTTCCGATCATATCATCTTTGTCTCATCATCGTCATCGTGCGCGTTATAATGCCGGNAATTTNAGAATTTCATACAGAAAAACNCGAACAATTGATACTTTTGTAAAAAAAAAAATAACAAATTTAAATTAAGGATATGATGAATTGATGTTTCCTCATTAAAATGAGAAGAGAAAAGGTACAATAAATGGCCTAAATATACTCTTTCAATTTGAAAAGATCCCACTAGTGTCGACACCAGTCTCATTCAGCCACAN"

**Gαo 5’ site sequence:**

"NNCTTGGCTTCGACGGGACCACCTCATGATGNAATAACATGAGGCCGCATAGGCCACTAGCTGGATCCCACTAGTGTCGACACCAGTCTCATTCAGCCACAAACTTGCGGCGGCGCCACTGGAAAGTGTGTGAGCCACAATNACAAGTCTGCGCAGGACGAAAACAGCGAGTGGTCNNGAGACAAGGACAACCCCAAAATCAACGAGTGCGCCCACGCGATATTCCTTTTTCCTCGCTGGTTTTCTTGTTTTTTTTTTTTTTTGGANANNTTNAAGNGGTTTTNNTNTTANNNNGGNGANNGCCATNGGNNCCNCNTCCCNGCNCACNNTTANTNNAATGNTTTNNNAANTTTNCNCCCTTNANGGGCNNAGCCAAANNNNATTACCNCAAANCAGTNGGCCTGCACCNGGANCCCCATAGGGTGNNACNCCANTTCTCATNTCANCC"

**CG6650 or endos 5’ site sequence:**

"ANGAGTNAGTTTGAGCACTGCTAGTTTCGATAAACGATATGGTTCCGAAATAACGCCTTCTGCTATCGAAGACAGTCTTGCGTCTGTAAAGTGATAAAATAATTTTACAATTGAGTCGCCTTGGGAATTTCAGTCACTGTCCCCACACCAAATACATAACTCGTGTAACAGCCTTATTTAGTCGAACAGCACATCATTTGCCGGAATCGTTAGCGAATAGCAGCACTCTCCGCTAAGTCGGCACGCTAATTGGCAAAACCGATTCGATTCCAGGGAAGGAGACACACTGATAGACGGGTGAAGAACAAAAGGAGAAGGAAAACAACAGTTAATCGTCAAATTTTACAGGCAAAAGGTGAGCAACTACGCCTTGCACACAAACAACCGCATCGACGAGCAAACGCACGCACACACAAGCCTTATCGCCTGCGTAGTGGTTGGTGTATATAAAAAATTTAAAACAATATTTGCACATCCGATTATTACTATAATATTTCGCGTCCACATATATTAGCAAGTGTGCGTGCGCCTGCCCTGGATTTACGTGCCAGTGTTTGTGCGAGCCTAGCGCGTTTGTGTCTGTGTGTGTGTGTAATGAAATGCAATTTTCTCTTGGCATTTTGCGCGCCTCCGATTTTCGCTGCCCCTCTGCAATTCCGCCGCCTCCCCCTCCTTTTGCCATTTAAGTTCTCCCGTGTCGCCATTGTCGCACCTTGGCTAATCCCAGTACTATCACCCGTAGGCAGCACACAATGAGCTCCGCGGAAGAAAACAGCAACAGCCCGGCCACCACGCCCCAGGACAACGAGACCACCGAGCAGGCTAACCTCACGGATCCCACTAGTGTCGACACCATNTTTTTTNNCCCCCCAAAA"

**CG6650 or endos 3’ site sequence:**

"AACCTTCGGTAGCTTCGGCTATCGACGGGACCACCTTATGTTATTTCATCATGGTGNAGANTNTNCATAANTGGGNNGNAGANCTTTCGGAGANGACCNCGANTCAAGCCGCGGATCTTACANNNGTGAGCTNTCATGNGGACCGAGGTGGACGTCCGGGGANNTGTTANAGCTACATANNCNGTCTACGATGTGNGNAANTCGNATNGGCTCNTGAGGATTGGATATTGAGGNNGGGTGNAGTTCCTATNNGGTNTCNCGGGTANNACTCNGTGAGGTGGNTNTTCGGGCNNCCCNGNTNAATNNCTNGATGGATATTTTGTCNCGAACTGAATGTATCCGAATGGCTTNCTTCTGANCCTGTNGNCCGCACTNGCNNTGTGAACNATNATCNGCTAATTNTNTCATNACTCATGGGCTTCACACTACACAGGCTTCCCCNCNANAGATCCCACTAAGAGNCTACGCCATATCCCGATCGCCCACATGTCACATACAAAATAGTTTGGAAAGCCCAAACTGAGGCGAACCAATCACACAACAATCCACGCCCACAATGACCGCCCTGCGATACATGGGATGGATCACCGGCTGTTCCGTCCTAACGGCCTTTGTTTCCATAATTTGGCAGGCATTCATCGCCCTGCAGACGCTTAACAGGACGACTGTCCTGCTCACCGGACTCCTGGCAATCGAGGGGTGAGTTCATTCGGCTACACTGCAGCACTAGCTATATATGCATTTGTATTTCAGATCCCACTAGTGTCGACACCAGTCTCATTNCAGCCACAA"

**Atg18b or CG8677 5’ site sequence:**

"ATNGNANCTTTCGGGGTAGCCTTCGGGCNNATCGGACGGGGGACCACCNTTTATGTTTATTTCATCATGGNTTCTATCTATCAACAAATTCCCCCATACACACAACACACACACACATAAATCATCACTCATTGGAACTGGGTTCGGGAACTGAACCAGCCGNGTTCACCAAATGCCATATATACCGTTAAATCATTTGTGTAACGGAAAATGTAAAATTGTTATTAAAAATTGAAATGGACAATATATATACATGTATCCGCGTATNTTTGCATTGGANAAATCATANAA"

**Atg18b or CG8677 3 site sequence:**

"GGNNTTNANANANGGTCGCAGCAAACAACAAAGGACGACGNCAAAACAGGTCGAATTTAGGTTTTCAATATCAAGCTGAAATCATTTCGCTTTGCTCATCTGCCATCGTCGATCAATATCTTGTGTACTCAAACGACGGGACTCTGCAACTGAATATCGCTGAAAAAAAGCATCTAAAGACCAGTGAAAGTGTAACTATATAAAAAAAATACAATCACCAAAGCACAAAGCTTCAGCAGCAATGACCACATATCAAATGAACTTCAACCAGGACTTTACGTAAGTAGCGACATCTGTGCTTAAATCCTTAAATCCCGGAAGGTCACCAANGCGANNAGTGGNNAACTATTGAGACACANANNTCNA"

**CG32795 5’ site sequence:**

"NTNTNATGTGAGCTTCCGGGTGCTCGCATATCTGGCTCTAAGACTTCGGGCCCGACGCAAGGAGTAGCCGACATATATCCGAAATAACTGCTTGTTTTTTTTTTTTACCATTATTACCATCGTGTTTACTGTTTATTGCCCCCTCAAAAAGCTAATGTAATTATATTTGTGCCAATAAAAACAAGATATGACCTATAGAATACAAGTATTTCCCCTTCGAACATCCCCACAAGTAGACTTTGGATTTGTCTTCTAACCAAAAGACTTACACACCTGCATACCTTACATCAAAAACTCGTTTATCGCTACATAAAACACCGGGATATATTTTTTATATACATACTTTTCAAATCGCGCGCCCTCTTCATAATTCACCTCCACCACACCACGTTTCGTAGTTGCTCTTTCGCTGTCTCCCACCCGCTCTCCGCAACACATTCACCTTTTGTTCGACGACCTTGGAGCGACTGTCGTTAGTTCCGCGCGATTCGGTGCGGTATTTCACACCGCATATGGTGCACTCTCAGTACAATCTGCTCTGATGCCGCATAGTTAAGCCAGCCCCGACACCCGCCAACACCCGCTGACGCGCCCTGACGGGCTTGTCTGCTCCCGGCATCCGCTTACAGACAAGCTGTGACCGTCTCCGGGAGCTGCATGTGTCAGAGGTTTTCACCGTCATCACCGAAACGCGCGAGACGAAAGGGCCTCGTGATACNCCTATTTTTATAGGTTAATGTCATGATAATAATGGTTTCTTAGACGTCAGGTGGCACTTTTCGGGGAAATGTGCGCGGAACCCCTATTTGTTTATTTTTCTAAATACATTCAAATATGTATCCGCTCATGAGACAATAACCCTGATAAATGCTTCAATAATATTGAAAAAGGAAGAGTATGAGTATTCAACATTTCCGTGTCGCCCTTATTCCCTTTTTGGCGGCATTTTGCCTTCCTGGTTTTGCTCACCCAGAAACGCTGGGGAAAGTAAAAGATGCTGAAGATCAGTTGGGTGCACGAGNGGGTTACATCGAACT"

**CG32795 3’ site sequence:**

"AATGTNCTTTNGGGTAGCCTTCGGCTATCGGACGGGACCACCCTTATGTTATTTCATCATGGCGCCAGNTGTGATATAGCCATTANGAGAATATGCATGAAGAAGGGACATGATAAGAAGATCCCACTTAGATGTTCGACACCAGTTCTCATTCAGCCACATCGACGATGTGCAGCTAATTTCNCCCGGCTCCACGTCCGCCCATTGGTTAATCAGCAGACCCTCGTTGGCGTAACGGAACCATGAGAGGTACGACAACCATTTGAGGTATACTGGCACCGAGCCCGAGTTCAAGAAGAAGGCGTTTTTCCATAGGCTCCGCCCCCCTGACGAGCATCACAAAAATCGACGCTCAAGTCAGAGGTGGCGAAACCCGACAGGACTATAAAGATACCAGGCGTTTCCCCCTGGAAGCTCCCTCGTGCGCTCTCCTGTTCCGACCCTGCCGCTTACCGGATACCTGTCCGCCTTTCTCCCTTCGGGAAGCGTGGCGCTTTCTCATAGCTCACGCTGTAGGTATCTCAGTTCGGTGTAGGTCGTTCGCTCCAAGCTGGGCTGTGTGCACGAACCCCCCGTTCAGCCCGACCGCTGCGCCTTATCCGGTAACTATCGTCTTGAGTCCAACCCGGTAAGACACGACTTATCGCCACTGGCAGCAGCCACTGGTAACAGGATTAGCAGAGCGAGGTATGTAGGCGGTGCTACAGAGTTCTTGAAGTGGTGGCCTAACTACGGCTACACTAGAAGAACAGTATTTGGTATCTGCGCTCTGCTGAAGCCAGTTACCTTCGGAAAAAGAGTTGGTAGCTCTTGATCCGGCAAACAAACCACCGCTGGTAGCGGTGGTTTTTTTGTTTGCAAGCAGCAGATTACGCGCAGAAAAAAAGGATCCCACTAGTGTCGACACCAGTTANTTTTNNNCCCNAA"

**Ttk 3’ site sequence:**
"ANCTTCGGTAGCTTCGGCTATCGACGGGACCACCTTATGTTATTTCATCATGCTTCGGTGCACACACAACATCGGCAAATCCTCCTCTCTCGAAAGGAACGCTCTTGCTCACACACACACGCACACAAACAGGCAATAGGGCTGCGGTTCGTGGAGACTTTTCGCTTGGTCCCCAAGTCTCCAAAAATGAGAGAGCCCGAACGCTAGAGCAAGAGAGCTGCTGTTGCTCTCTAGCTTGCGCCTTTGACTTTTTGAATTTTTAATTTCCAGCAAAACGGGGTTAAAAAGAAAAACACGCTGAAAAAAAGCTAGGAAGCCACCGACAACAACAACAGCAGCAACGGTTCGGGTGTGCAACAGTTGATGATTTGTTGTATCCCCCGCATTTTCCCGCCGCTGTAGAAGTGCATTGTAGGTTTTGTTGTTGTAAGGGCAACTTCTGGGCTGGGCTCTTTATTGAAACCATTTGGGGTTGGGCTGCAGCTTTGCTGGGGTTTTCCGGACCTCTGAAATATCGGAAAGCATGTGGAAAAGCGAGAGCCAGTAAAGAAGGCGTGGTGGGATGTACAGTGGATACTCAAATAAATTGACTGAAGTTTAAATGTTGAATCTTTTTTTTTACAAACCTTAGACATTTGTAAAACATTCACAACATAAATCGAAATAACAAAGTTTGGCTCAAAAAGGCACTTCACAAAAAATATGACGAGGATCATAAAAATATGTAATTATTCACAACAACAAAATTAATGGGTGTTTCACTGCATGGCTTTGTGCTGGCTGGTTGGCAGGAGCGTTTTTCTGAGGTTTTTTTTTTTAGGAGAGGGATTACGTAAGCGGGCTGTAAATTCTACAAGTGGATTGACTCAACAGCCATTGGGGATCGGATCCCACTAGTGTCGACACCATNNNTTTTTNCNCCCCAAA"

**Larp 3’ site sequence:**

GACTCGGTAGCTTCGGCTATCGACGGGACCACCTTATGTTATTTCATCATGGGTTGGGGTTGCCAGGCGCCGGCAAAGTATCAACATGCGTCGTGACGCGTCGCGACGTTTGTGAAGCGTTTGGAACGGTGAAATGGCACACTTTCGATCTTTCAAAATGTCAAATATTTTAGGGTGCTTTAAAATACATAGTATAGTTTGAGTTAATTTATTTATTTATATTAACAAATTGCACTATACTTTCGTGTAATAAAGCTGAAGATTTACAGATTTAAATCTTAACACATTCGCTGTTTTGGAACATAGAATATATATACATAGTTTACACTTATAGCAACAATTTATTACTTCAATATCAATTCATCCCAAAGCTAATGATATTCTTTGTTGTATAGTATTGATCTTAGTATTTATTAATTAGTACAACGATTGCGATGAAATTTGTATTTAACAGTTAATATAATATGTTAAAAGCGATATTATTAACGATAAATATATTTTGACTAACAAGCAGACCGAACATTAAGAAATTTAGCACAAGCCACCTTCGTCCTCGGCGTCGTTGGTTTCCTGCGTGAGAGAAAGAGAGAAAATACGAAATTCTTGTTAGCTATTTAGTTTACTGCTTCGCACTGTCCCAAGTCTTCTATCGACAACAAAGGCAGATTCAATCGCCTATCGATAACATCATAATAAGAGCCAAATTTACAAAACAAAAATTGCAAATTCCATTTTTAGAATAAAAATACTCACAGCAGCAGTGTCGCCAGCCTTCTTCTCCATCTGTATACCCTGAGGTAGTGGAATGTTTTTAGATAGAATAGCGTACTCAACATCCATATGTCGTATGAATGGGGATGCCAGCATCCTAGCAGCTAACTCGGGATCCCCNCTAGTGTCGACACACCNTTTTTNCCCCC

**CaM 5’ site sequence:**

"AGCCTGCGCNNCTTACCGTTTCGACGCTCTCTCGCATAACCGTTTCACACACTTTCTCGTCTCTCTCTATGTGCAATTATTTCGTTTGCGTTATCAGTTCCGTTTTGTGCTGCGTCTCTCTCTTTCCGTTTCTTTCAGCATTTCATTTTTTTTTTGCGCCTTGCGCTTGTCACTTTTATCGGCGCCTGCCTTTTCTTGTTTTCTTCCATAACCGTTTCATTTGCCGCATTGCGGTTATTTTGCTGTTTCGCCTTCGCCTTGGTTCGTCCGCTTCGGTCGTTCATTCGTTCGGCTCCGCGTATTCGTATTCGTGTGCGTGTGAGTGAGCGTATGGGTGTGTGTGTTGGCGGCTGCCTGTTCCACTTTGCCTTTTCGCGCAAGAATTTTATTGTAACGGCACAGGCGAACGATGGGGGAGGAGGAGCGCTGCAAATGAAGAAAAATTCCTGCTAGACGCCGCCGCTTAACCACATAATCCAATTTTCTTGCCGCCCTCTTTGCGCTTTCTTCTCTCGTTCGTTTGCTTTCCTCTGCGCTTTTTGATAAGAAAGCCCCACGCCCACTGCGATGCCCACACCCGCGCCACACCTTCTTTCAGCCTCATCTGGTGTCGCCTAATTTAATTAGCGATTTTACAGCTCACGCAGAATGCAAACAGCTTTTAATTAAAATATATCGCATCGCTTTCTACGTGGCCAATTTGTTTCAGTGATTCAACCAACGCTGCTAACTTGGCCCAGAAGCAAACATTTGCCAAGGGTTTTACTGACTGATCTTCAAAGTTTGTTGATCTTTAGCTCGGCTAATTATGAGATACGTTTGTCTGTATATCTTATTTGTTTCCGTTGAAAATGAGCCAACATTCATATAAATTTCCATGAGTCACTGNGTTCCTGGGCTGACTTCATCATATGATCATATGGNCNATGANGAANGCCCGAAGANAGGGNGGGNGNCANGTAANAAGGNAAAAGACTGGCTANGGCTCAGTANCACAGTTGNTTGGANCCACTAGGNCAAACACNNNTTTTANCCAAAAAN"

**CaM 3’ site sequence:**

"AGTNCCTTCGGTAGCTTCGGCTATCGACGGGACCACCTTATGTTATTTCATCATGAGCAGGAGAAATGCCTCTAAAGAATTTTTTCACCATTTCCATGGGGCCGTGTCTAACCTCAATTTTTGCGGCCAAGTGTGCGGTAAATAAAAAATACAACCAGAAAGCAAAGAACAAGCGAACAACGAACGGCGGGGCAAAGAGGAGAGGCCAATAATGCCGGGACCGGCGACTTTGTTGTTCATCATCAATATATATTCACACATTTTCCACTGATAAGCAAAATGGAGGGCGCTTTTCTATTATTTGCGGGGATCCCACTAGTGTCGACACCAGTCCTTTTCAGCCACAA"
